# Connecting the food and agriculture sector to nutrition interventions for improved health outcomes

**DOI:** 10.1007/s12571-022-01262-3

**Published:** 2022-02-01

**Authors:** E. Duncan, L. Ashton, A. R. Abdulai, T. Sawadogo-Lewis, S. E. King, E. D. G. Fraser, S. Vosti, J. Haines, F. Knight, T. Roberton

**Affiliations:** University of Guelph, Guelph, ON Canada

**Keywords:** Nutrition-sensitive agriculture, Food value chain, Food security, Nutrition security

## Abstract

To achieve the Sustainable Development Goal of zero hunger, multi-sectoral strategies to improve nutrition are necessary. Building towards this goal, the food and agriculture sector must be considered when designing nutritional interventions. Nevertheless, most frameworks designed to guide nutritional interventions do not adequately capture opportunities for integrating nutrition interventions within the food and agriculture sector. This paper aims to highlight how deeply connected the food and agriculture sector is to underlying causes of malnutrition and identify opportunities to better integrate the food and agriculture sector and nutrition in low and middle income countries. In particular, this paper: (1) expands on the UNICEF conceptual framework for undernutrition to integrate the food and agriculture sector and nutrition outcomes, (2) identifies how nutritional outcomes and agriculture are linked in six important ways by defining evidence-based food and agriculture system components within these pathways: as a source of food, as a source of income, through food prices, women’s empowerment, women’s utilization of time, and women’s health and nutritional status, and (3) shows that the food and agriculture sector facilitates interventions through production, processing and consumption, as well as through farmer practices and behavior. Current frameworks used to guide nutrition interventions are designed from a health sector paradigm, leaving agricultural aspects not sufficiently leveraged. This paper concludes by proposing intervention opportunities to rectify the missed opportunities generated by this approach. Program design should consider the ways that the food and agriculture sector is linked to other critical sectors to comprehensively address malnutrition. This framework is designed to help the user to begin to identify intervention sites that may be considered when planning and implementing multi-sectoral nutrition programs.

**Supplementary Information:**

The online version contains supplementary material available at 10.1007/s12571-022-01262-3.

## Introduction

Reducing malnutrition is a key priority identified in the Sustainable Development Goals and a necessary step in moving towards a world with zero hunger (United Nations Economic and Social Council, 2019). However, we are not currently on track to meet the goal of zero hunger by 2030, as approximately 750 million people globally are exposed to severe levels of food insecurity and this statistic is trending upwards (FAO et al., 2020). Additionally, with the ongoing impacts of the COVID-19 pandemic and the potential long-term, detrimental impacts on global food security and malnutrition (Roberton et al., 2020), new approaches are needed to address food insecurity and malnutrition.

The problem of malnutrition is multi-faceted and requires action through a multisectoral approach that includes healthcare, education, water and sanitation, social protection, and food and agriculture (World Health Organization, 2018; Gillespie et al, 2013; Reinhardt & Fanzo, 2014; The World Bank, 2013; Garrett et al., 2011; Banking on Nutrition Partnership, 2017; Bezanson & Isenman, 2010; FAO et al., 2021; King et al., 2020). Each of these sectors is a unique complex system and plays a role in addressing the underlying causes of malnutrition. To create a multi-sectoral approach to address malnutrition, we must be clear on what each sector can contribute to the issue. For the food and agriculture sector, it is primarily responsible for the provision and acquisitions of food.

However, capacity to innovate and address global food security and malnutrition faces other ongoing barriers in the food and agriculture sector, such as the decline in public investment in agriculture (FAO et al., 2018). Worldwide, there has been a 37 percent decline in the ratio of government spending on agriculture as compared with the sector’s contribution to total economy, with the ratio decreasing from 0.42 in 2001 to 0.26 in 2017 (United Nations Economic and Social Council, 2019). In addition to the financial challenges, current approaches to nutrition are narrowly focused on what a person eats and need to account for the broader environmental and economic conditions affecting the food and agriculture sector (Garnett et al., 2014; Willett et al., 2019). This is reflected by the fact that in countries with multi-sectoral policies for nutrition, the health sector is primarily responsible for implementing these policies, with the agriculture sector trailing behind (WHO, 2018). The gap between health and agriculture and other critical sectors requires attention and innovative approaches to co-design multi-sectoral interventions to address the issue of malnutrition (including both undernutrition, and overweight and obesity). Understanding how the food and agriculture system influences nutrition and its relationship to other sectors can help facilitate intervention and coordination within a multi-sectoral approach.

The UNICEF conceptual framework for undernutrition continues to serve as the base framework for the international nutrition community (UNICEF, 1998). However, this framework leaves a gap in our understanding of the underlying health factors and how to intervene upon the factors causing malnutrition. To address these limitations, we present a framework to expand on the current UNICEF framework that presents more upstream detailed factors affecting nutrition using a multi-sectoral framing, and focuses on implementation to guide governments and program planners from the food and agriculture sector. In order to create a framework that connects the food and agriculture sector to improved nutrition outcomes, we draw on literature from the agricultural studies, nutrition, and public health. Through interweaving this evidence, we aim to show how researchers can take a multi-sectoral lens when developing tools for nutrition intervention program developers, practitioners, and policy makers.

This paper will present first present the methodological approach to identifying and describing these food and agriculture sector components that ultimately comprise a detailed framework of how the food and agriculture sector impacts nutrition outcomes. We explicitly take a food *and* agriculture approach, emphasizing the importance of food systems from production to consumption, rather than solely focusing on the agricultural sector. Secondly, we present our results which include overview of the framework and a more detailed explanation of the three specific pathways for nutrition-sensitive agriculture. Next, our results elaborate on three specific interventions – fortification, home production, and nutrition-sensitive value chains – that have been established as crucial interventions for malnutrition (Fiorella et al., 2016; Girard et al., 2012; Hawkes & Ruel, 2008; Masset et al., 2012; Saltzman et al., 2013). Finally, we discuss the importance of multi-sectoral approaches, the key role of farmer behavior in affecting nutrition outcomes, and the importance of developing a framework to capture the complexity of how the food and agriculture sector impacts nutrition.

## Methods

To create the food and agriculture sector framework, we followed a multi-step process. 1) We reviewed past frameworks, conceptual diagrams, and nutrition sensitive agriculture (NSA) pathways. 2) Using this data, we identified the key food and agricultural system components. 3) We compiled a database of literature for each component. 4) We designed the first iteration of the framework. 5) The framework underwent a review process from experts until we arrived at a consensus about the components of the framework. This process began in March 2019 and was completed in September 2020 by an interdisciplinary research team with expertise in public health, nutrition, food security, agriculture, and international development.

The first step was reviewing literature on agriculture-nutrition interlinkages and compiling a database of conceptual frameworks that outline pathways for agricultural activities to affect nutrition outcomes. The inclusion criteria for this review involved papers that provide conceptual frameworks of components within the food and agriculture sector or that spoke broadly about these components, their interconnectedness, and impact on nutrition outcomes. Another inclusion criteria was that papers focused on low- and middle-income countries (LMICs). In this initial step, we identified 18 peer-reviewed papers that feature conceptual frameworks for food systems and nutrition. Also, a key focus of the review was the literature around NSA pathways within the food and agriculture sector.

When referring to nutrition-sensitive interventions, we adopt the definition by Ruel and Alderman (2013, p.537), which states, “nutrition-sensitive interventions or programs are those that address the underlying determinants of fetal and child nutrition and development— food security; adequate caregiving resources at the maternal, household and community levels; and access to health services and a safe and hygienic environment—and incorporate specific nutrition goals and actions”. To do this, we identify and describe the food and agriculture sector components that support the delivery of NSA interventions.

**Fig. 1:**
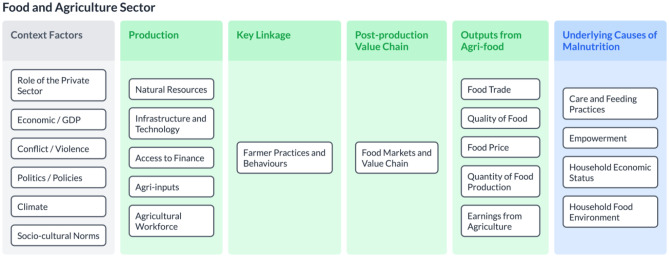
Visualization of food and agriculture system components for nutrition interventions framework

The starting point of data analysis and synthesis was the well-established malnutrition framework—the UNICEF conceptual framework for undernutrition (UNICEF, 1998). In Fig. 1, we highlight our key contribution to expanding this model. In our model, we provide more detail the basic causes rather than focusing on potential and actual resources. Building on Harris and Nisbett’s (2020) work that unpacks the black box of ‘context’ to elaborate on resources, structure, ideas, and power, we explicitly include climate, as well as more specific contextual factors such as the role of conflict/violence and the role of the private sector. In between the contextual factors and underlying causes, we found there was an important gap that did not address the role of various sectors that are involved in multi-sectoral approaches to nutrition interventions. Therefore, in this paper we focus specifically on the development of the food and agriculture sector for nutrition outcomes. Future research requires that each sector should continue to be further elaborated to provide the detail necessary for effective nutrition interventions (See King et al., 2020 for a description of connecting the health sector to nutrition interventions; Zavala et al., 2021 for WASH and nutrition; and Xu et al., 2021 for the education sector).

From there this analysis of the UNICEF model, we noted that there were many high-level frameworks of nutrition-sensitive agriculture interventions that built on each other to provide conceptual linkages between agri-food and nutrition (Dorward, 2014; Gillespie et al., 2012; Kadiyala et al., 2014; Kanter et al., 2015; Masset et al., 2011; Pandey et al., 2016; Webb, 2013). More detailed frameworks that focused on certain aspects of the food and agriculture sector and its impact on nutrition were also explored to provide a more comprehensive level of understanding to specific components such as aquaculture (Kawarazuka, 2010), agricultural productivity (Headey & Hoddinott, 2016), commercialization (Von Braun, 1995), healthcare for agriculture (Hawkes & Ruel, 2006), value chains (Gelli et al., 2015; Maestre et al., 2017; Zuberi et al., 2016), and gendered components (Chung, 2012). We also included analysis of nutrition causal analysis frameworks and initiatives (Manners et al., 2015; Mutegi & Korir, 2016; see https://www.linknca.org). A more specific and detailed framework holds the potential to assist with nutrition causal analysis which aims to understand causes of malnutrition with local contexts and plan appropriate interventions.
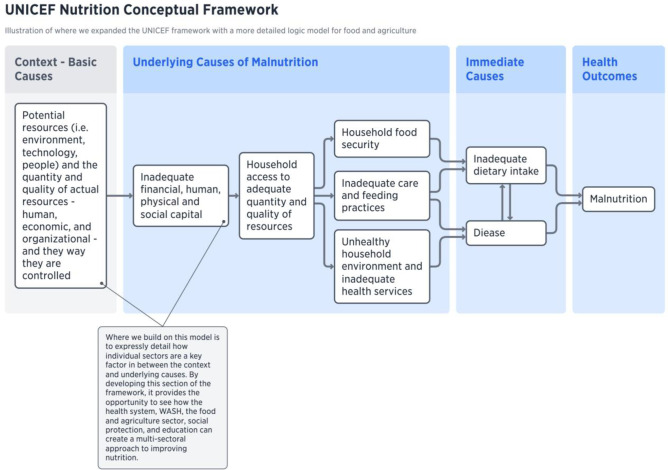


Using these frameworks, we organized the identified body of literature around 12 key food and agriculture sector components and 35 sub-components. To do this, we recorded what were the most common components of other frameworks, noting similarities and differences in terminology, and also components that were outliers, such as ones that were only occasionally included in the frameworks. Through this iterative process, we determined a comprehensive list of components and sub-components. The sub-components allowed for more details within the framework. In order to verify that these components were indeed central to the framework, we returned to conduct a secondary review of the literature involving a broad scope of bodies of literature from interdisciplinary food systems studies, nutrition studies, and development studies.^1^ In this review, we created a database of 232 papers that support the inclusion of each of the components and how they connect to each other. This database focused on food and nutrition security literature which provides evidence for how the agricultural components of our framework can affect nutrition programs, and in turn, affect population level nutrition outcomes. In addition, for each component of the food and agricultural sector, we identified indicators within public datasets, mostly compiled by the FAO, that could aid nutrition program developers to measure each component. For example, a key component of the food and agriculture sector is food prices, therefore an important indicator for this component is the food price index data generated by the FAO. Not only did the causal pathways include each component, but they also identified linkages between each component, supported by the literature, to show causal effects through the framework.

The next step was to organize the components in a logical flow, creating pathways between each component, and then attaching the evidence from our database (both literature and indicators) to each section. This was an iterative process with the research team and took approximately seven months of work before we felt the framework was ready to be reviewed by experts. The expert review process took place during a one-day workshop in October 2019 with 32 nutrition and nutrition-sensitive agriculture experts, representing some of the key organizations and research institutes working in the field of nutrition including: the World Food Programme, the Bill and Melinda Gates Foundation, the International Food Policy Research Institute, the Global Alliance for Improved Nutrition, the Arrell Food Institute, and Johns Hopkins Bloomberg School of Public Health. Experts were selected from a group of academics leading the research on these topics, all experts who assisted in developing the overall framework were also invited. As the audience for the framework is policy makers and program planners for use in design and evaluation of evidence-based nutrition programs, we chose to include experts from nutrition stakeholders. Our aim was to have a diverse review panel of expertise to ensure a comprehensive final framework. Experts were asked specific questions, such as whether they agreed with the high-level framework and if this was the best way to conceptualize nutrition, and if anything was missing from the framework.

This review process resulted in addition of components and ensuring that the descriptors for each component reflected the taxonomy used in the nutrition sector. After including the edits from the workshop, five follow-up consultative calls with experts were conducted to share the updated framework and solicit final revisions on the organization and inclusion of specific components. To summarize, we first reviewed the literature of conceptual frameworks of food systems and nutrition. Then we drafted our framework and created a database of literature to support this framework. Finally, we presented this framework to experts and modified accordingly. After the final round of changes, we arrived at a final version of the framework, presented in the results.

## Results

Herein, we present our food and agriculture sector framework. Our results begin with an overview of the framework and an overview of the traditional six NSA pathways and their associated components that we identified through our review of the literature. We use the term components (and associated sub-components) to define the topics that comprise our framework. For example, agri-inputs are one component of the agricultural production process, and we can further define this component through subcomponents such as soil enhancement, crop protection, seeds (including biofortified and nutrient dense seeds), and farm animal genetics and inputs. We provide more detail through highlighting three pathways in the framework that summarize the traditional six NSA pathways outlined in Table 1. We have consolidated the six NSA pathways (Herthford & Harris, 2014) into three for the purpose of explaining the framework and focus on (1) agriculture as a source of food, (2) describes the effects of food prices and income on nutrition, and finally the (3) considers the role of women in agriculture on nutrition. Within each of these pathways, our framework identifies the specific food and agriculture sector components as specific sites that can be targeted for improved nutrition outcomes. The components are not unique to any specific pathways, as there are overlaps between the figures presented here.

**Table 1:** Nutrition-sensitive agriculture pathways and their key components of the food and agriculture system

PATHWAY	DESCRIPTION	KEY FOOD & AGRICULTURE SYSTEM COMPONENTS	REFERENCES
1. Agriculture as a source of food	Agriculture is the source of food for household consumption	• Natural resource • Agricultural workforce • Agri-inputs • Infrastructure & technologies • Access to finance	Gillespie & Bold, 2017; Ruel et al., 2018; Hawkes & Ruel, 2008; Gillespie et al., 2012; Jaenicke & Virchow, 2013
2. Agriculture generates income for food and other nutrition-related expenditures	The sector generates income for those working in it for food and non-food expenditures	• Food markets and value chain • Quality of food • Quantity of food • Food prices • Earnings from agriculture • Food trade	Silvestri et al., 2015; Hawkes & Ruel, 2006;
3. Agricultural policy and other factors that affect food prices	Food prices, determined by contextual factors such as policy, affect people’s ability to purchase food	• Food markets and value chain • Quality of food • Quantity of food • Food prices • Earnings from agriculture • Food trade	Pinstrup-Andersen & Babinard, 2001; McMichael, 2001; Hawkes et al., 2009; Reardon et al., 2003; Gomez & Ricketts, 2013
4. Women’s employment decision making & resources allocation	Women’s engagement in agriculture has effects on intrahousehold decision making and resource allocation	• Agricultural education, trainings, & promotions • Commercialization • Production diversity • Earnings from agriculture	Malapit et al., 2015; Ruel, Alderman et al., 2013; Akter et al., 2017
5. Women’s employment time allocation & childcare	Women’s engagement in agricultural activities is dependent on available time. Also, the demand for women’s time for agricultural work may negatively affect childcare and other activities	• Availability of workforce • Adoption and use of inputs/technology • Utilization of time • Earnings from agriculture	Hillesland et al., 2016; Johnston et al., 2018
6. Women’s employment health & nutritional status	Women’s employment in agriculture directly affects their personal health. and nutritional status due to energy expenditure and safety of work	• Health, safety, & decent labour • Adoption & use of inputs/technology • Earnings from agriculture	FAO, 2015; FAO, 2016; Croppenstedt & Muller, 2000; Girard et al., 2012

**Fig. 2:**
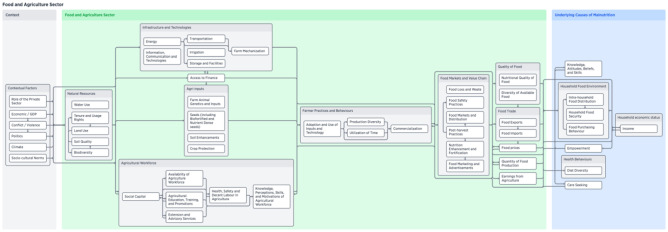
Detailed visualization of food and agriculture system components and sub-components for nutrition interventions framework

Figure 1 provides a high-level depiction of the framework by connecting contextual factors to the main components of the food and agriculture sector, and then finally relating those components to the underlying causes of malnutrition. Figure 2 shows a more highly detailed version of the first figure, which includes the sub-components. The contextual factors include the overarching situation that can contribute to both agricultural production and higher rates of malnutrition. These contextual factors are more situated at a national level, and in some cases, at an international level – for example, economic factors like GDP or the role of the private sector, the presence of conflict or violence, politics (especially agricultural and trade policies), biophysical factors like climate change, and socio-cultural norms. Within the food and agriculture sector, production components – the material necessary to produce food – and linked to the post-production value chain through farmer practices and behaviours. Ultimately, the outputs from the sector that affect nutrition outcomes include quality and quantity of food, food prices, food trade, and earnings from agriculture. These outputs are directly tied to the underlying causes of malnutrition which are determined by key components such as care and feeding practices, (women’s) empowerment, and household economic status and food environment. These were determined by our analysis of the UNICEF framework and remain similar to the original, however when we elaborate more specifically through the use of subcomponents.

Our framework aims to demonstrate that there are multiple entry points for interventions in agri-food to improve nutritional status. As an example, we highlight how the food and agricultural sector can be instrumental in delivering three key NSA interventions: fortification, home production, and nutrition-sensitive value chains. The purpose of selecting these interventions is two-fold: firstly, they were identified as key NSA interventions from our literature review, and secondly, they aptly demonstrate how our detailed framework is particularly useful for identifying areas in which to intervene. Finally, our results present important linkages between the food and agriculture sector and other sectors that influence nutrition outcomes from a multi-sectoral approach.

Table 1 provides an overview of the traditional six NSA pathways and their food and agriculture system components that were identified through our review of the literature. Conceptual frameworks showing how the food and agriculture system can affect nutrition outcomes have already been well established and adopted in the literature, namely: (1) through agriculture as a source of food for household consumption; (2) by generating income for food and nonfood expenditures; (3) through the effects of agricultural policy and food prices on consumption; (4) through women's employment in agriculture and its effect on intrahousehold decision-making and resource allocation; (5) through women's employment in agriculture and time available for childcare and child feeding; and (6) through women's employment in agriculture on their own health and nutritional status. These pathways are elaborated above in Table 1, where we highlight the key food and agricultural system components within each. We derived the components for each pathway from our initial literature review of conceptual frameworks for nutrition. Through our framework and by detailing pathway components, we show the connections and overlaps between these pathways and the multiple points along the food production chain at which interventions can influence nutrition. This process of identifying pathway components allows for a broader food and agriculture systems lens to show the interconnectedness of the previously established pathways. For example, by identifying the key food and agriculture system components, nutrition program developers can first determine which NSA pathway needs addressing in a specific context and then use the framework to identify more specific areas of intervention. As evidenced by Table 1, interventions that aim to address agriculture as a source of food for agricultural households should focus on the components of natural resources, agricultural workforce, agri-inputs, infrastructure and technologies, and access to finance.

### Presenting the framework

Within the framework both components and subcomponents are shown in the following figures. We use arrows between these components to denote relationships, where stronger relationships are shown through a solid line and less clear relationships are shown through a dotted line. The expert group agreed upon these relationships; however this paper does not describe these detailed relationships in detail so that framework users can analyze and apply the framework to their specific context. The framework is exceptionally detailed, therefore we selected to display the components and subcomponents in this paper to show their relevance to the six NSA pathways.

**Fig. 3:**
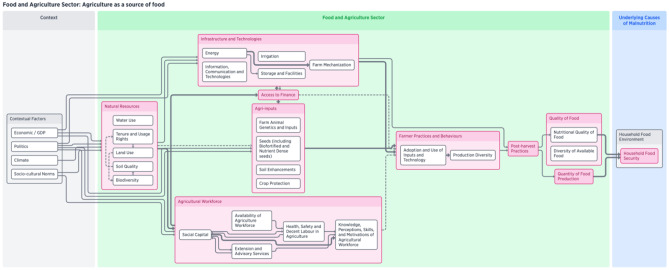
Visualization of food and agriculture system components framework—Agriculture as a source of food

The first key section of the framework (Fig. 3) presents the resources necessary for agricultural production including natural resources, agri-inputs, the agricultural workforce, access to finance, and infrastructure and technologies. These components provide the base necessities for producing food. The contextual factors that are influential for this pathway include: ecomomic and political factors, increasingly climate vulnerability, and sociocultural norms as production is linked to food preferences. In terms of the NSA pathways, these components are critical for food access because they affect the quantity and quality of available food, which ultimately helps to determine the household food environment. Figure 3 illustrates the components of this first section of the framework.

A key component that links the production components to the consumption components of the agri-food system is farmer practices and behaviour, which we identify as a ‘key linkage’ (Kuehne et al., 2017). While farmers are constrained in their decision-making by a variety of factors from the production side – such as availability of resources and access to finance and technologies – these considerations ultimately affect the production diversity, degree of commercialization, farmers’ utilization of time, distribution of agricultural products, and finally consumption. Here, we note one key overlap in pathways and components is women’s time use (See Fig. 5) – both women and men’s practices around spending time farming versus other competing priorities is both key in this production to consumption pathway and is elaborated further in the gender and empowerment pathway. The constraints faced by farmers under these key linkages directly influence the food environment available to households (Peterman et al., 2014). Farmers are likely to be influenced in their decision-making by their agricultural training, availability and quality of agricultural extension, and access to markets for the commercial sale of their production. Additionally, along this pathway, the contextual factor of climate vulnerability has a profound effect on the components that are critical to food production.

**Fig. 4:**
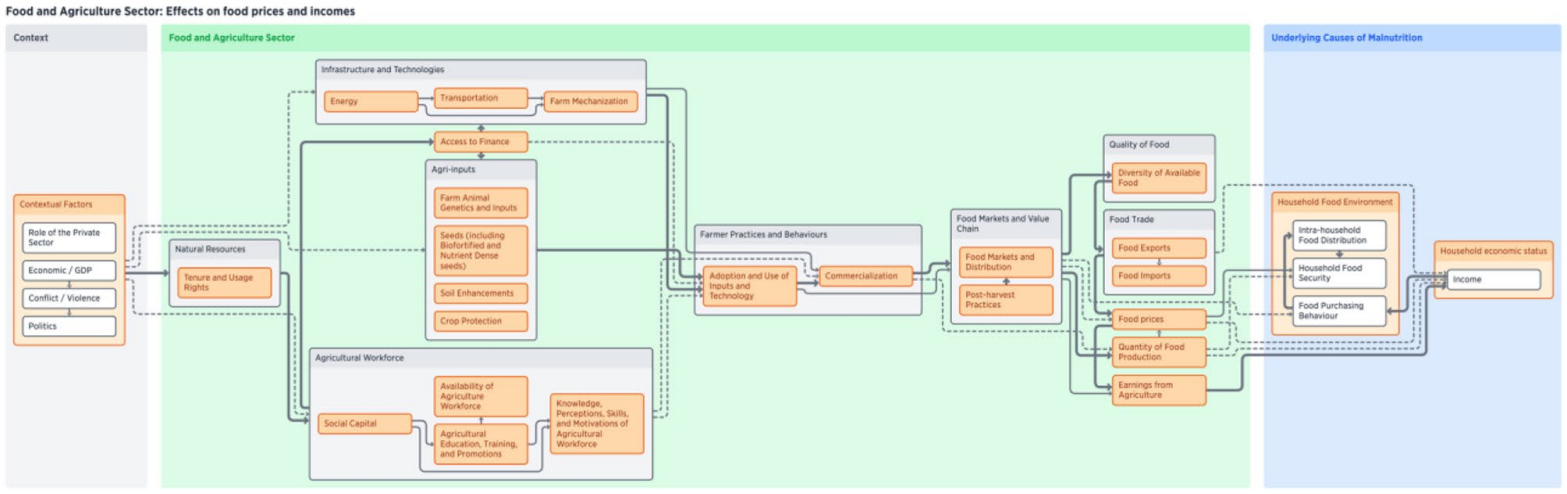
Visualization of food and agriculture system components framework—Effects on food prices and incomes

Income and food prices (Fig. 4) are two distinct components of the food and agriculture sector that directly and indirectly influence nutritional outcomes, and they comprise two of the NSA pathways. Income from agriculture can either be from waged labour or directly from the sale of commodities produced. Food insecurity is primarily influenced by insufficient earnings from agriculture^2^ as this component is directly tied to household socio-economic status which is the most significant determinant of household food security levels (Sen, 1990; Silvestri et al., 2015). Additionally, food price shocks can also lead to food insecurity at the household level. Components in the contextual factors section of the framework, such as the presence of conflict or violence or economic changes, also influence food prices. Furthermore, food prices are a crucial link between agriculture and nutrition. International and national agricultural policies – and in some cases regional/state policies – regulate supply and demand of agricultural production. Food trade (referring to cross border trade) is influenced by these policies, as well as by the value chain including components such as food safety practices, marketing, and post-harvest practices such as value-added processing (Gomez & Ricketts, 2013). Ultimately, these components set food prices, which determine sellers’ incomes and buyers’ purchasing power, thus shaping household food budgets.

Farmer practices and behaviour, particularly the extent of commercialization, is the key linkage connecting the production and consumption components of the food and agriculture sector related to income and food prices. The ‘downstream’ sections are focused on consumption and capture the food value chain (as shown in Fig. 4). This section includes key components such as food markets, prices, trade, as well as quantity and quality of food production. Leveraging the value chain has been identified as a potential strategy for intervention (Ruel et al., 2018; FAO, 2017). The components of the value chain are particularly important to nutritional outcomes because it is these components that are directly linked to the underlying causes of malnutrition, such as household food security (Villa-Rodriguez et al., 2015). Access to food markets, increased trade through globalization, and the rise in supermarketization (shift from traditional, fragmented local markets to larger, centralized wholesale markets such as grocery stores) in developing areas have caused significant changes in food consumption, namely the increase in consumption of highly processed foods (Hawkes et al., 2009; McMichael, 2001; Pinstrup-Andersen & Babinard, 2001; Reardon et al., 2003).

**Fig. 5:**
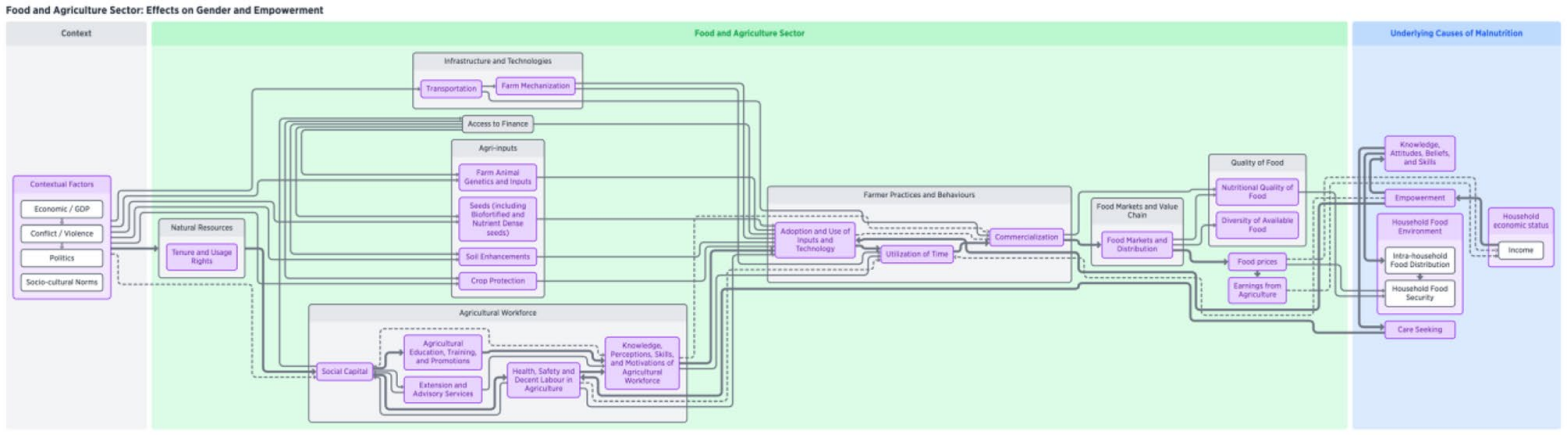
Visualization of food and agriculture system components framework—Effects on Gender and Empowerment

Finally, we show the food and agriculture sector components that are directly related to women’s involvement in agriculture. There are three NSA pathways related to this topic (Fig. 5), including women’s empowerment, utilization of time, and health and nutritional status of women. Farmer practices and behaviours, as a key linkage, is an area that contributes to agriculture’s effect on nutritional outcomes because it is directly related to utilization of time and women’s social status and empowerment (Hillesland et al., 2016). Women’s engagement in agriculture and access to decent labour and other resources affects time available for self and childcare. Gendered cultural practices often dictate the amount of time that women spend participating in agriculture, as well as balancing other household responsibilities, caregiving, and potentially engaging in other income-generating activities (Johnston et al., 2018). In some cases, it is not just balancing these activities, but there are trade-offs, for example, a woman might stop breastfeeding because she needs to work all day planting, harvesting, or weeding.

In turn, women’s utilization of time is a strong influencing factor on women’s social status, empowerment, and nutritional status of themselves and their children. While empowerment is likely to be considered an underlying driver of nutritional outcomes and is connected to a range of practices and behaviours, it is directly tied to the food and agricultural sector as participation in agriculture can impact women’s access to and control over resources and assets, such as livestock, home food production, or specific crops (Malapit et al., 2015). Participation in agriculture has the potential to increase women’s empowerment and positive nutritional outcomes as women gain more decision-making power over production choices, as well as over intra-household allocation of food and resources. However, these pathways are not always linear and may not work as intended. Employment in agriculture should not be directly equated with empowerment. The trade-offs between engaging in agricultural employment rather than other activities such as care-giving or other income-generating activities might undermine women’s decision making power (Johnston et al., 2018). Additionally, while women may be employed in agriculture, they may still be excluded from land ownership which has implications for access to credit. Thus, land tenure and usage rights are a key component in the pathway to ensuring women’s empowerment.

Also represented within this section of the framework (Fig. 5) is an important connection by which agriculture can affect nutrition outcomes: women’s health and nutritional status. A primary input into the food and agricultural sector is the workforce. While the availability and training of the workforce is important in general, research demonstrates that there are gendered differences observed. For example, migration affects the availability of the agriculture workforce and often leads to the feminization of agriculture, which has effects on productivity, women’s empowerment, and nutrition (Stanley, 2015). These effects have the potential to be positive on aspects of empowerment, such as women may gain more decision-making power and autonomy if men in the family have migrated to urban areas, or migration may have detrimental effects by dramatically increasing the workload of women. Health, safety, and decent labour (meaning the opportunity for everyone to engage in productive work that offers a fair income, workplace security, social protection, and prospects for personal development and social integration, while respecting human rights (FAO, 2015) in agriculture are areas for interventions into this pathway, as women’s health can be compromised through exposure to agriculture-associated health hazards and diseases, such as through exposure to agri-chemicals and drudgery (FAO, 2015; FAO, 2016). Additionally, nutritional requirements for women working in agriculture can be affected by increased energy expenditure during the manual labour required for production (Croppenstedt & Muller, 2000). This component is described in our framework through the availability of labour, with a focus on the distribution of labour by gender. Many studies suggest women’s health, nutritional status, and time are key factors influencing agricultural productivity and income received from agriculture, which provides an opportunity for intervention (FAO, 2017; Ruel et al., 2013; Lambrecht et al., 2018).

### Places to Intervene

To highlight areas in the framework that support nutrition-sensitive interventions within the food and agriculture sector, we discuss three interventions: fortification, home production, and nutrition-sensitive value chains. The framework demonstrates there are many more sites along the stages of food production than those traditionally considered in such programs to implement these interventions, as well as sites to expand to other potential interventions (Meadows, 1997). The interventions of fortification and nutrition-sensitive value chains are focused on the population level, whereas home production is an intervention that is focused on the household level. Our framework is not designed to target a specific level of intervention, but instead aims to demonstrate the connections between components within NSA pathways. We expand on three areas of intervention to show importance of this framework.

**Fig. 6:**
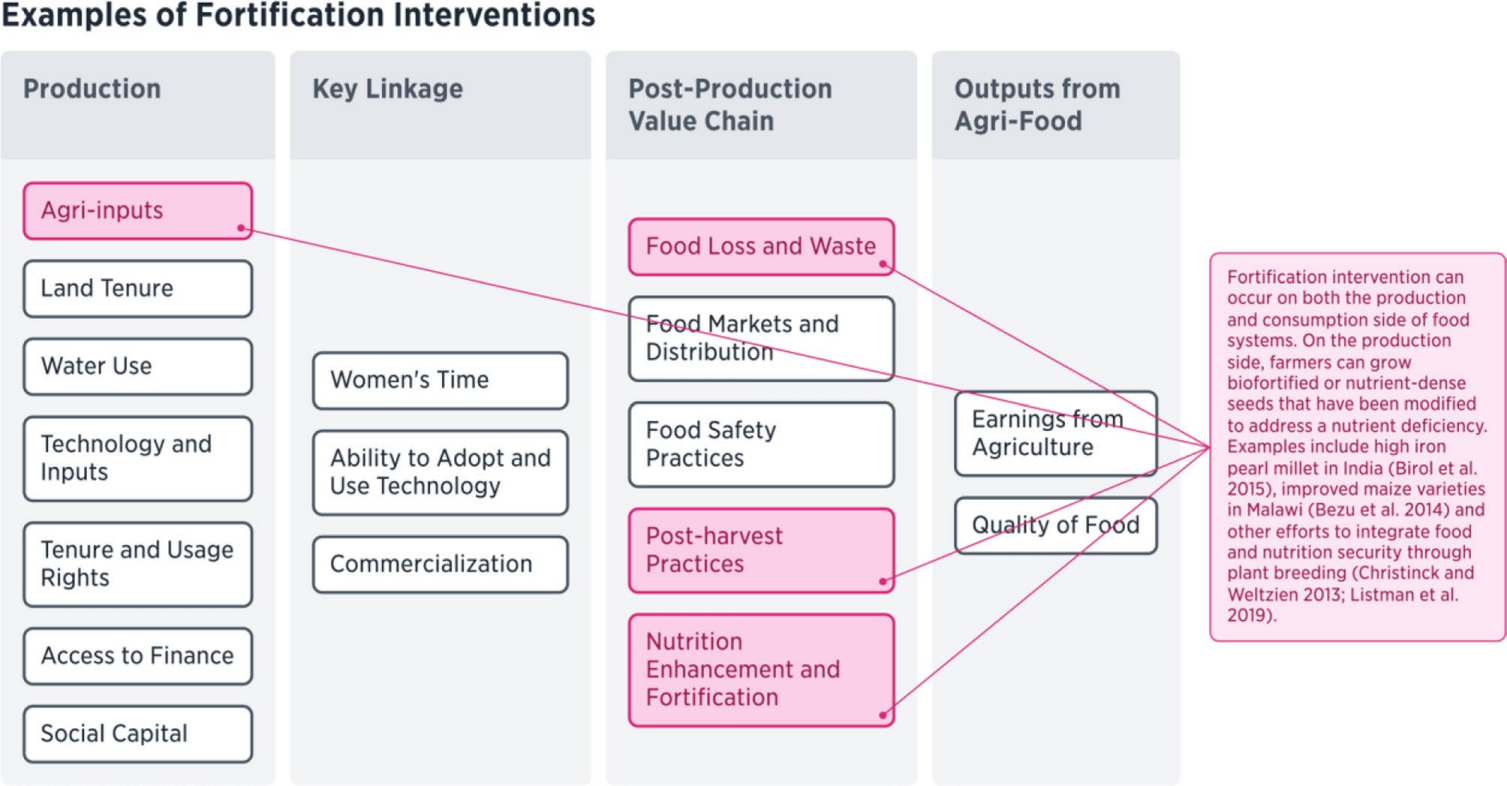
Examples of Fortification Interventions

Firstly, there are multiple ways for nutrition programs to provide fortification (see Fig. 6). This intervention can occur on the production side (commonly referred to as biofortification), where farmers grow biofortified or nutrient-dense varieties that have been modified to address a nutrient deficiency. Examples include high iron pearl millet in India (Birol et al., 2015), improved (high yielding) maize varieties in Malawi (Bezu et al., 2014) and other efforts to integrate food and nutrition security through plant breeding (Christinck & Weltzien, 2013; Listman et al., 2019). There are also examples of fortification via fertilizers, often referred to as agronomic fortification. The GeoNutrition Project has demonstrated results that micronutrient deficiencies of selenium can be alleviated through adding selenium fertilizers to staple cereal crops in Malawi (Joy et al., 2019). Additionally, the intervention of fortification can occur along the consumption side of the food and agriculture sector framework through nutrient enhancement during food processing. Nutrition-sensitive value chains provide an opportunity for fortification of grain products, such as wheat or maize flour. Fortification of wheat flour with folic acid is one example of postharvest fortification, while much research has been conducted on how to scale-up rice fortification to address micronutrient deficiencies (Dwyer et al., 2015; de Pee et al., 2014). While these two sites of intervention may seem obvious, by creating a framework that reveals the complexity of the food and agriculture sector, we can shine a light on other components of nutrition-sensitive interventions. By delineating all the components of the food and agriculture sector relevant to nutrition, it creates a model for sites of intervention. Nutrition programs can then target these specific sites. One example of a component that could address nutrition is at the site of food markets and post-harvest practices, where there is the potential for food loss and waste (Parfitt et al., 2010). Through enhanced storage techniques or fortification to prolong the shelf life of foods with high nutritional value (Cheema et al., 2018), such as fruits and vegetables, food loss and waste provides another independent point of intervention for fortification that can lead to increased food security and better nutritional outcomes (Hodges et al., 2011; Kumar & Kalita, 2017).

**Fig. 7:**
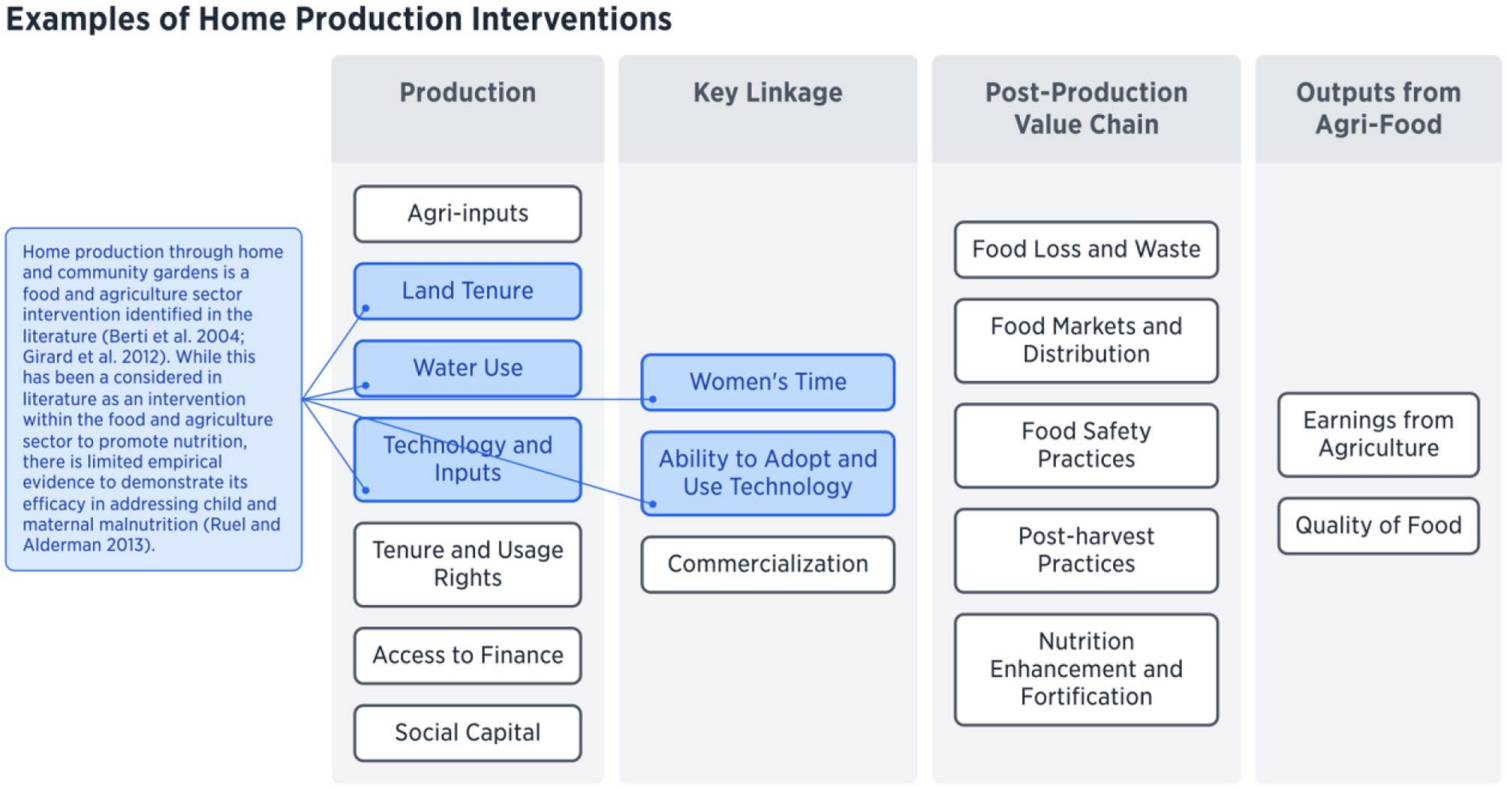
Examples of Home Production Interventions

As the production section of the framework demonstrates (see Fig. 7), critical components for home production include land tenure and usage rights, water use (including water availability and infrastructure), and technology and inputs. Home production through home gardens is another key food and agriculture sector intervention identified in the literature (Berti et al., 2004; Girard et al., 2012). While this has been a consistent intervention within the food and agriculture sector to promote nutrition, Ruel et al. (2013) indicated that there was limited empirical evidence to demonstrate its efficacy in addressing child and maternal malnutrition in terms of anthropometric outcomes. However, NGOs have advocated for its success (such as Hellen Keller International, Concern International, and German Agro-Action) and there is growing evidence of the benefits of home production on nutrition outcomes (Hendriks et al., 2020; Lal, 2020; Rammohan et al., 2019). However, it has been useful in other outcomes, such as enhancing women’s empowerment in the areas of increasing meeting with other women, purchasing decisions, and health care decisions (Olney et al., 2016, 2017). By identifying the components necessary in the food and agriculture sector for home production, it is evident that there are many “upstream” factors that may constrain this intervention, such as land tenure and usage rights, water availability, and access to technologies and inputs. Thus, interventions at these sites may be required before home food production can promote positive nutritional outcomes. Additionally, upstream interventions that target women’s activities and promote empowerment have been found to be more likely to have a nutritional effect (Ruel et al., 2013; Malapit et al., 2015; Akter et al., 2017). Considering key components identified by the framework, such as women’s utilization of time and their ability to adopt and use technologies and inputs, nutritional interventions in these areas could prove more successful. Therefore, exploring these important upstream entry points to improve home production more holistically may be more effective than simply intervening solely to improve home production practices, but this strategy still needs more empirical evidence.

**Fig. 8:**
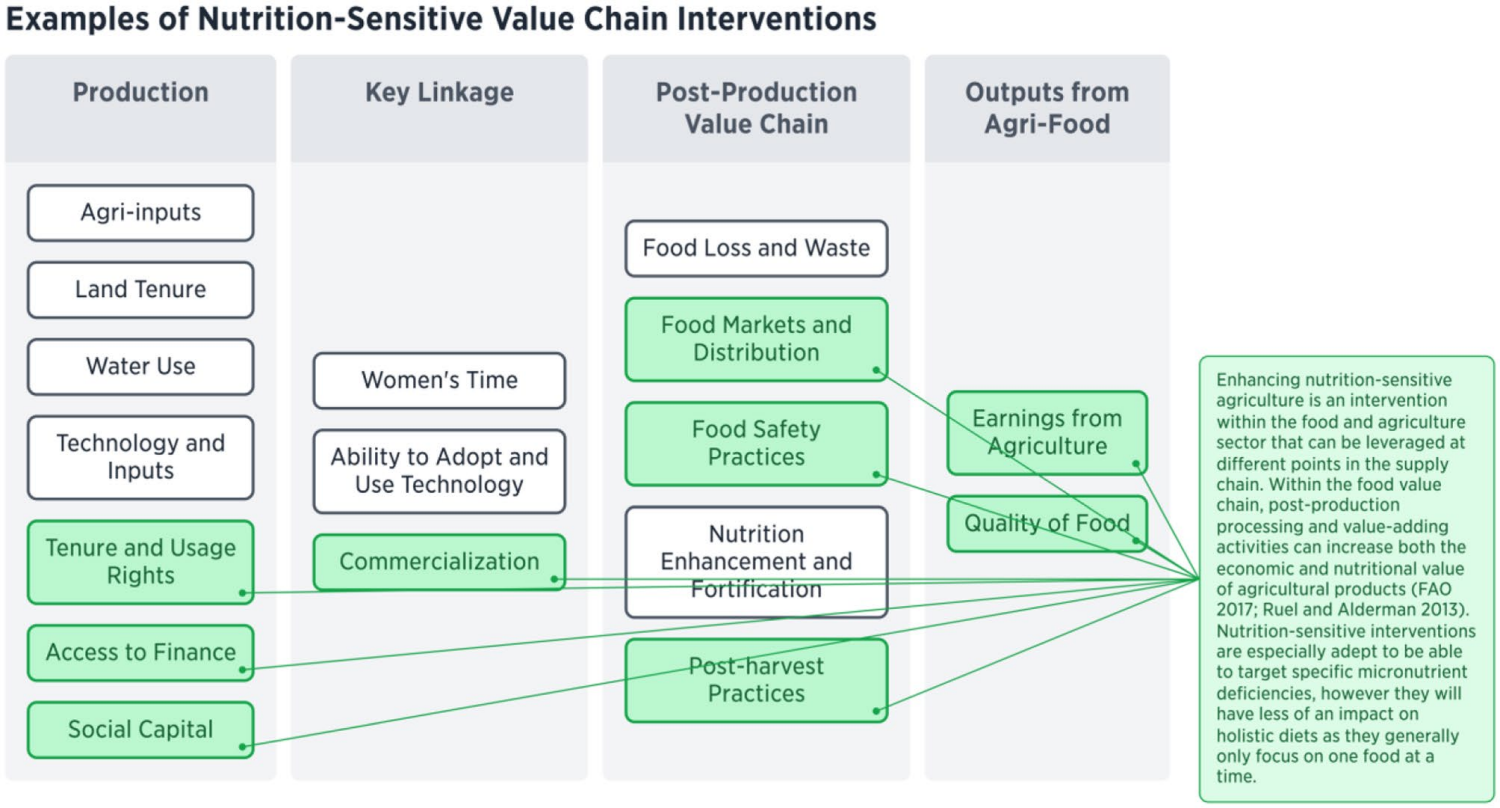
Examples of Nutrition-Sensitive Value Chain Interventions

Lastly, nutrition-sensitive value chains are another site of potential intervention within the food and agriculture sector (see Fig. 8) (Hawkes & Ruel, 2012; Gelli et al., 2015). Within the food value chain, post-production processing and value-adding activities can increase the both the economic and nutritional value of agricultural products. The components detailed in Fig. 5 of our framework detail the aspects of a nutrition-sensitive value chain. As most of the framework targets rural populations, interventions in a nutrition-sensitive value chain can create rural–urban linkages by ensuring that agricultural producers receive increased income from their products and urban consumers receive products that are fresh and nutritionally beneficial (FAO, 2017). Interventions to provide more nutritional benefits through the value chain will require coordination among food system actors and are important for stimulating the supply and demand of micronutrient-rich foods (Ruel et al., 2013). Value chain interventions are especially adept to be able to target specific micronutrient deficiencies, however they will have less of an impact on holistic diets as they generally only focus on one food at a time. They also have the potential to address food-borne illnesses through food safety interventions, which will have a positive outcome on nutritional status.

## Discussion

After presenting our framework, we discuss three key points which have been highlighted through this research: (1) the relevance and contribution of the detailed framework; (2) the need for multi-sectoral approaches to alleviating malnutrition; and (3) the importance of farmer behaviour and practices on nutritional outcomes.

Our framework expands on existing conceptual frameworks reviewed by breaking down larger elements in the stages of food production into their smaller constituents, which will allow program planners to identify a wider range of options and specificities of NSA interventions during the intervention design stage. Within each larger component are sub-components that together contribute to more specific targeting of interventions. Within each sub-component, there is the opportunity to create indicators that provide evidence for the importance of that component. These indicators are comprised of specific datasets, mostly provided by the FAO. For the nutrition interventions often diet-related indicators would be useful in measuring the success of the intervention. Several important indicators require farm-level production data, which could provide more specific information for nutrition interventions in the food and agriculture sector, however there is a lack of farm-level production data that is collected and accessible to nutrition program developers.

In this exercise of mapping out the food and agriculture sector’s potential connections to nutrition outcomes and reviewing relevant literature and existing frameworks, we have found some important knowledge gaps. In drawing connections between production inputs and nutrition literature, we found that this should be an area for future research to better explain how inputs affect productivity, food availability, and nutritional quality. There has been limited examination of the food and agriculture sector through a nutrition lens. This is evidenced by the lack of standardized indicators for the several of the major components of the framework. There is a significant body of work being undertake to address this through the IMMANA (Innovative Methods and Metrics for Agriculture and Nutrition Actions) Evidence and Gap Map to identify the tools, methods, and metrics needed to understand the complex connections between agriculture and nutrition (Sparling et al., 2019). Datasets for these indicators are necessary to contextualize conditions and status of the food and agriculture sector in relation to nutrition outcomes. We identify a need for more qualitative datasets from interviews and surveys to provide evidence for components, such as farmer practices and behaviours. Ultimately, food is for nourishment, yet the agricultural literature has overwhelmingly evaluated food as a commodity, providing an opportunity for increased research on NSA interventions.

One of the largest challenges facing the food and agriculture sector is the effect of climate change, one area for future research that we have identified is to map the connections between climate-smart agriculture and nutrition-sensitive agriculture. As climate change is affecting many of the key components for food production, such as biodiversity (Frison et al., 2011; Thrupp, 2000), soil quality (Gomiero, 2016), and water use (Hanjra & Qureshi, 2010), there will be a negative effect on nutrition outcomes (IPCC, 2019). The framework described here holds the possibility to provide a base for future research that aims to connect climate and nutrition by demonstrating the linkages between components that are connected to climate as a key contextual factor. For example, a changes in the context of climate may influence access to water for farmers, such as an increase in droughts. As shown in the framework, this has implications for whether farmers might decide to adopt irrigation affecting their ability to commercialize or produce a sufficient quantity of food, ultimately linking to some of the underlying causes for malnutrition.

The framework allows us to clearly tease out how agri-food sector connects to other sectors to influence nutrition outcomes. Results show some of the key connections between the food and agriculture sector to other crucial sectors are necessary for the delivery of nutrition interventions—there are four other key sectors intersecting with food production, namely: water and sanitation (WASH), education, healthcare, and social protection. Firstly, the components of the WASH sector that have an important connection to food safety are the safe reuse of wastewater, animal and human excreta, and greywater (Bracken et al., 2007). Access to water is essential for food production, and as many smallholder farmers rely on rainfed agriculture, reuse of water is one option during unpredictable rainfall (De Fraiture et al., 2010). However, this practice can have impacts on food safety if proper food hygiene practices are not implemented (Godfrey et al., 2010). Secondly, there are several connections between the education sector and the food and agriculture sector to improve nutrition outcomes because schools are considered a crucial site for nutrition interventions. Also, educational curriculum is necessary to provide not only nutritional education, but agricultural education and training. Integrating nutrition-sensitive agricultural practices into formal curriculum is an important intervention (Jaenicke & Virchow, 2013); and there is a growing evidence base that early childhood development is a key aspect of education-nutrition linkages (FAO et al., 2021). Agriculture and education connections through school meal programs (local procurement) also affects nutrition status in children and population health. While the healthcare sector is important for the delivery of nutrition-specific interventions, it also serves as a support for the food and agriculture sector through helping to maintain the health and safety of farm labourers by ensuring they have access to healthcare.

One of the strongest ties for a multi-sectoral approach to improving nutrition outcomes is linking the food and agricultural sector to social protection. One of the main social protection interventions that is extended to farmers on the production side of the framework is the provision of fertilizer and other agri-inputs to help ensure crop production, which has implications for food security and nutrition outcomes. Other social protection interventions aim to address consumption concerns on the ‘downstream’ end of the framework. Food transfers and emergency food aid are components of the social protection sector that have shown positive effects of improving household food security (Doocy et al., 2017). However, they may have adverse effects on the food and agriculture sector by depressing local prices and contributing to a lack of demand for locally produced food in areas where markets are functioning (Gelan, 2007). However, cash transfers can aid in addressing food insecurity by increasing budgetary allotment for food, in areas where markets are functioning, and they provide the ability to purchase from local producers, thereby stimulating the local economy (Seal et al., 2017; Doocy et al., 2020). Organizations such as the World Food Programme have well-established practices of assessing market functionality before making decisions regarding appropriate transfer modalities for social protection, such as the market functionality index (WFP, 2020). Nutritional programming should seek to understand the multifaceted linkages between these two sectors to ensure successful outcomes.

One key area to improve impacts of nutrition interventions is through farmer practices and behaviours. What farmers do on daily or seasonal basis on their farms and livelihoods can help enhance the quality and quantity of food available, as well as their own socio-economic wellbeing and food markets. Interventions that encourage farmers to adopt innovations and technologies (Kuehne et al., 2017) have the potential to diversify, increase, and enhance the quality of food produced. Thus, interventions targeting farmer practices and behaviours will have effects on ‘downstream’ components such as food availability, access, and stability of supplies (Garrity et al., 2010). The framework that we describe in Fig. 3 hinges on this component, thus making it a key area for intervention. Influencing farmer practices and behaviours is complex, and our framework identifies the sub-components, including utilization of time, adoption of technologies, commercialization, and production diversity as areas to intervene.

One particular challenge that was encountered with creating this framework was the question of scale. References to the food and agriculture sector in low- and middle-income countries (LMICs) and the linkages to nutrition, can often be overly characterized by subsistence agriculture. However, these systems are more complex, with many actors engaging at different levels of production scale, commercialization, participation in markets, and subsistence farming. Capturing the nuance of scale of production within a framework such as ours is particularly limiting, yet we have included components, such as commercialization and farmer practices and behaviour, that try to explain the dynamic nature of farming production by showing the linkages between these and the outputs of the food and agriculture sector (Ntakyo & van den Berg, 2019).

## Conclusion

This paper has presented the conceptual framework aspect of the forthcoming digital tool with respect to the food and agriculture sector. Overall, the aim is to build a better bridge between the food and agriculture sector and nutrition goals in order to address the pressing challenges of the SDGs with respect to zero hunger. The food and agricultural sector is ultimately tied to health outcomes through nutrition. We have described areas to strengthen the food and agriculture sector to provide better nutrition outcomes as well as detailed where there are connections between other sectors that are key for delivering nutrition interventions. In mapping out the key components of these sectors, it is clear that a multi-sectoral approach is needed to address the pressing challenge of malnutrition. The framework presented here can inform this approach moving forward because it provides a more detailed conceptual framework to delineate connections between the food and agriculture sector and other nutrition-related sectors.

## Supplementary Information

Below is the link to the electronic supplementary material.

Supplementary file1 (PDF 18734 KB)

## Data Availability

Not applicable.

## Footnotes

1 Three of the authors independently worked to gather literature using key word searches of the components and subcomponents. Then our individual lists were amalgamated to ensure commonalities. Any discrepancies over whether an article should be included in our final list were discussed amongst these authors until consensus was reached.

2 Earnings from agriculture specifically refers to the income received by the agricultural workforce, including sales from own production and wages received from agriculture/food production work.
