# Supplementary material for: Connecting the food and agriculture sector to nutrition interventions for improved health outcomes
Source: Food Secur. 2022 Feb 1;14(3):657–75. doi: 10.1007/s12571-022-01262-3 (PMC8804081; doi:10.1007/s12571-022-01262-3)
Supplement: Supplementary file 1 — Supplementary file1 (PDF 18734 KB) [file 12571_2022_1262_MOESM1_ESM.pdf]

Conceptual Frameworks

RADAR

Nutrition Visualizer Validation Workshop

18 October 2019

Institute for International Programs

Johns Hopkins Bloomberg School of Public Health

Table of Contents

General Nutrition 8

1. United Nations Children's Fund: UNICEF’s approach to scaling up nutrition for mothers and their children. In. Edited by Programme Division. New York: UNICEF; 2015. 8
2. SPRING: A Systems Thinking and Action for Nutrition: A Working Paper. In. Edited by USAID/ Strengthening Partnerships R, and Innovations in Nutrition Globally (SPRING) Project. Arlington, VA; 2015. 9
3. Black RE, Victora CG, Walker SP, Bhutta ZA, Christian P, de Onis M, Ezzati M, Grantham-McGregor S, Katz J, Martorell R et al: Maternal and child undernutrition and overweight in low-income and middle-income countries. Lancet (London, England) 2013, 382(9890):427-451 10
4. World Bank: Improving Nutrition Through Multisectoral Approaches. Edited by World Bank. Washington, DC; 2013 2013. https://openknowledge.worldbank.org/handle/10986/16450 License: CC BY-NC-ND 3.0 IGO. 11

Health System 12

1. Londoño JL, Frenk J: Structured pluralism: towards an innovative model for health system reform in Latin America. *Health policy (Amsterdam, Netherlands)* 1997, 41(1):1-36. 12
2. World Health Organization: Everybody's business -- strengthening health systems to improve health outcomes : WHO's framework for action. In. Edited by WHO. Geneva, Switzerland; 2007. https://apps.who.int/iris/handle/10665/43918 13
3. World Health Organization: The World Health Report 2000. In: *Improving Performance.* Edited by WHO. Geneva, Switzerland; 2000 13
5. World Bank: Improving Nutrition Through Multisectoral Approaches. Edited by World Bank. Washington, DC; 2013 2013. https://openknowledge.worldbank.org/handle/10986/16450 License: CC BY-NC-ND 3.0 IGO. 14
4. Roberts M, Hsiao W, Berman P, Reich M: Getting Health Reform Right – A Guide to Improving Performance and Equity. New York; 2003 15
5. Atun R, Menabde N: Health systems and systems thinking: Open University Press; 2008 15
6. Kleczkowski B, Roemer M, Van Der Werff A: National health systems and their reorientation towards health for all. Guidance for policy-making. 77th edition. Geneva: World Health Organisation; 1984. 16
7. World Health Organization: The World Health Report 2000. In: *Improving Performance.* Edited by WHO. Geneva, Switzerland; 2000 17
8. Sacks E, Morrow M, Story WT, Shelley KD, Shanklin D, Rahimtoola M, Rosales A, Ibe O, Sarriot E: Beyond the building blocks: integrating community roles into health systems frameworks to achieve health for all. 2019, 3(Suppl 3):e001384. 17

Food and Agriculture 18

1. Sobal J, Kettel Khan L, Bisogni C: A conceptual model of the food and nutrition system. *Social Science & Medicine* 1998, 47(7):853-863. https://doi.org/10.1016/S0277-9536(98)00104-X 18

2. Chung K: An Introduction to Nutrition-Agriculture Linkages. Direccao de economia, Ministerio da agricultura, Republica de Mocambique 2012. https://www.spring-nutrition.org/sites/default/files/chung_msu_2012_ag-nutrition_linkages.pdf..... 20

20

3. Kennedy E, Bouis HE: Linkages between agriculture and nutrition: implications for policy and research. Washington, DC: International Food Policy Research Institute; 1993. http://ebrary.ifpri.org/utils/getfile/collection/p15738coll2/id/125519/filename/125550.pdf..... 23

4. Hawkes C: Identifying Innovative Interventions to Promote Healthy Eating Using Consumption-Oriented Food Supply Chain Analysis. *J Hunger Environ Nutr* 2009, 4(3-4):336-356. https://www.tandfonline.com/doi/full/10.1080/19320240903321243..... 24

Continued: Hawkes C: Identifying Innovative Interventions to Promote Healthy Eating Using Consumption-Oriented Food Supply Chain Analysis. *J Hunger Environ Nutr* 2009, 4(3-4):336-356. https://www.tandfonline.com/doi/full/10.1080/19320240903321243..... 25

5. Kanter R, Walls HL, Tak M, Roberts F, Waage J. A conceptual framework for understanding the impacts of agriculture and food system policies on nutrition and health. 2015. https://link.springer.com/article/10.1007%2Fs12571-015-0473-6..... 26

6. Gillespie, S; Harris, J; Kadiyala, S; (2012) The Agriculture-Nutrition Disconnect in India: What Do We Know? Washington, DC; 2012. https://researchonline.lshtm.ac.uk/id/eprint/1440425..... 28

7. Food and Agriculture Organization of the United Nations Rome: Compendium of nutrition-sensitive indicators in agriculture. Rome, Italy; 2016 29

8. Herforth A, Harris J: Improving Nutrition through Agriculture Technical Brief Series: Understanding and Applying Primary Pathways and Principles; 2014..... 30

9. Kadiyala S, Harris J, Headey D, Yosef S, Gillespie S: Agriculture and nutrition in India: mapping evidence to pathways. Annals of the New York Academy of Sciences 2014, 1331(1):43-56. https://nyaspubs.onlinelibrary.wiley.com/doi/full/10.1111/nyas.12477 31

32

10. Pandey VL, Mahendra Dev S, Jayachandran U: Impact of agricultural interventions on the nutritional status in South Asia: A review. *Food Policy* 2016, 62:28-40. https://doi.org/10.1016/j.foodpol.2016.05.002 32

11. Black RE, Singhal A, Uauy R (eds): International Nutrition: Achieving Millennium Goals and Beyond. Nestlé Nutr Inst Workshop Ser. Nestec Ltd. Vevey/S. Karger AG Basel, © 2014, vol 78, pp 93-109. https://doi.org/10.1159/000354946 33

12. Webb P: Impact Pathways from Agricultural Research to Improved Nutrition and Health: Literature Analysis and Research Priorities. In: ICN2 Second International Conference on Nutrition. Rome, Italy: Food and Agriculture Organization of the United Nations (FAO) and the World Health Organization (WHO) 2013. http://www.fao.org/3/a-as573e.pdf..... 34

Education 38

1. Efevbera Y, Bhabha J, Farmer PE, Fink G: Girl child marriage as a risk factor for early childhood development and stunting. *Soc Sci Med* 2017, 185:91-101.doi:10.1016/j.socscimed.2017.05.027 38

2. Masset E, Gelli A: Improving Community Development by Linking Agriculture, Nutrition and Education: Design of a Randomised Trial of “Home-Grown” School Feeding in Mali. *Trials* 2013, 14:55. doi:10.1186/1745-6215-1455 38

3. Rangel CN, Nunn R, Dysarz F, Silva E, Fonseca AB: Teaching and learning about food and nutrition through science education in Brazilian schools: an intersection of knowledge. In: *Ciência & Saúde Coletiva.* 2014, 19:3915-3924. Available from: http://www.scielo.br/scielo.php?script=sci_arttext&pid=S1413-81232014000903915&lng=en. http://dx.doi.org/10.1590/1413-81232014199.12552013. 39

4. Masset E, Gelli A: Improving Community Development by Linking Agriculture, Nutrition and Education: Design of a Randomised Trial of "Home-Grown" School Feeding in Mali. *Trials* 2013, 14:55. doi:10.1186/1745-6215-1455 40

5. Levinger B. School Feeding, School Reform, and Food Security: Connecting the Dots. *Food and Nutrition Bulletin* 2005, 26(2_suppl2):S170-S178. doi:10.1177/15648265050262S207. 41

6. McCoy DC, Zuilkowski SS, Fink G: Poverty, physical stature, and cognitive skills: Mechanisms underlying children's school enrollment in Zambia. *Developmental Psychology* 2015, 51(5):600-614. doi:10.1037/a0038924 42

7. Torres I, Benn J: The rural school meal as a site for learning about food. *Appetite*. 2017, 117:29-39. doi:10.1016/j.appet.2017.05.055 43

8. Studdert LJ, Soekirman, Rasmussen KM, Habicht J-P: Community-Based School Feeding during Indonesia'S Economic Crisis: Implementation, Benefits, and Sustainability. *Food Nutrition Bulletin*. 2004, 25(2):156-165. doi:10.1177/156482650402500208 44

9. Schwartzman F, Mora CAR, Bogus CM, Villar BS: Background and elements of the linkage between the Brazilian school feeding program and family farming. *Cad Saude Publica*. 2017, 33(12):e00099816. doi:10.1590/0102-311X00099816 45

WASH 47

1. Cumming O, Cairncross S: Can water, sanitation and hygiene help eliminate stunting? Current evidence and policy implications. *Matern Child Nutr* 2016, 12(S1):91-105. https://doi.org/10.1111/mcn.12258........ 47

2. Wood S, Foster J, Kols A: Understanding why women adopt and sustain home water treatment: Insights from the Malawi antenatal care program. *Social Science & Medicine* 2012, 75(4):634-642. https://doi.org/10.1016/j.socscimed.2011.09.018 48

3. Dangour AD, Watson L, Cumming O, Boisson S, Che Y, Velleman Y, Cavill S, Allen E, Uauy R: Interventions to improve water quality and supply, sanitation and hygiene practices, and their effects on the nutritional status of children. *Cochrane Database of Systematic Reviews* 2013(8). https://doi.org/10.1002/14651858.CD009382.pub2........ 48

4. Dearden KA, Schott W, Crookston BT, Humphries DL, Penny ME, Behrman JR, Cueto S, Duc LT, Escobal J, Fernald L *et al*: Children with access to improved sanitation but not improved water are at lower risk of stunting compared to children without access: a cohort study in Ethiopia, India, Peru, and Vietnam. *BMC Public Health* 2017, 17(1):110. https://doi.org/10.1186/s12889-017-4033-1........ 50

5. Curtis V, Schmidt W, Luby S, Florez R, Touré O, Biran A: Hygiene: new hopes, new horizons. *The Lancet Infectious Diseases* 2011, 11(4):312-321. https://doi.org/10.1016/S1473-3099(10)70224-3........ 50

6. Jenkins MW, Scott B: Behavioral indicators of household decision-making and demand for sanitation and potential gains from social marketing in Ghana. *Social Science & Medicine* 2007, 64(12):2427-2442. https://doi.org/10.1016/j.socscimed.2007.03.010 51

7. WHO and UNICEF Joint Monitoring Programme for Water Supply, Sanitation, and Hygiene: Service level ladders for monitoring drinking water, sanitation, and hygiene. 2017. 52

9. WHO and UNICEF Joint Monitoring Programme for Water Supply, Sanitation, and Hygiene: Classification of improved and unimproved drinking water and sanitation facility types. 2017. 53

10. Mbuya MNN, Humphrey JH: Preventing environmental enteric dysfunction through improved water, sanitation and hygiene: an opportunity for stunting reduction in developing countries. *Matern Child Nutr* 2016, 12 Suppl 1(Suppl Suppl 1):106-120. https://doi.org/10.1111/mcn.12220 54

54

11. Prendergast AJ, Kelly P: Interactions between intestinal pathogens, enteropathy and malnutrition in developing countries. 2016, 29(3):229-236. doi: 10.1097/QCO.0000000000000261 55

12. Raihan MJ, Farzana FD, Sultana S, Haque MA, Rahman AS, Waid JL, McCormick B, Choudhury N, Ahmed T: Examining the relationship between socio-economic status, WASH practices and wasting. *PloS one* 2017, 12(3):e0172134. https://doi.org/10.1371/journal.pone.0172134 56

13. World Health Organization: Guidelines for drinking-water quality: fourth edition incorporating the first addendum. Geneva: World Health Organization; 2017. 57

14. World Health Organization. Guidelines on sanitation and health. Geneva: World Health Organization; 2018. 58

15. Jones KD, Thitiri J, Ngari M, Berkley JA: Childhood Malnutrition: Toward an Understanding of Infections, Inflammation, and Antimicrobials. *Food and Nutrition Bulletin* 2014, 35(2_suppl1):S64-S70. https://doi.org/10.1177/15648265140352S110 65

16. Humphrey JH: Child undernutrition, tropical enteropathy, toilets, and handwashing. *The Lancet* 2009, 374(9694):1032-1035. https://doi.org/10.1016/S0140-6736(09)60950-8 66

17. Dreibelbis R, Winch PJ, Leontsini E, Hulland KRS, Ram PK, Unicomb L, Luby SP: The Integrated Behavioural Model for Water, Sanitation, and Hygiene: a systematic review of behavioural models and a framework for designing and evaluating behaviour change interventions in infrastructure-restricted settings. *BMC Public Health* 2013, 13(1):1015. https://doi.org/10.1186/1471-2458-13-1015 67

18. Mosler H-J: A systematic approach to behavior change interventions for the water and sanitation sector in developing countries: A conceptual model, a review, and a guideline. *International journal of environmental health research* 2012, 22:431-449. DOI: 10.1080/09603123.2011.650156 68

19. Nguyen-Viet H, Zinsstag J, Schertenleib R, Zurbrügg C, Obrist B, Montangero A, Surkinkul N, Koné D, Morel A, Koottatep T *et al*: Improving Environmental Sanitation, Health, and Well-Being: A Conceptual Framework for Integral Interventions. *EcoHealth* 2009, 6:180-191. 69

20. Gentry-Shields J, Bartram J: Human health and the water environment: using the DPSEEA framework to identify the driving forces of disease. *The Science of the total environment* 2014, 468-469:306-314. 70

21. Campbell OMR, Benova L, Gon G, Afsana K, Cumming O: Getting the basic rights - the role of water, sanitation and hygiene in maternal and reproductive health: a conceptual framework. *Trop Med Int Health* 2015, 20(3):252-267. 73

Social Protection 74

1. Adato M, Bassett L: Social protection to support vulnerable children and families: The potential of cash transfers to protect education, health and nutrition. *AIDS Care - Psychol Socio-Medical Asp AIDS/HIV*. 2009;21(SUPPL. 1):60-75. doi:10.1080/09540120903112351 74

2. Floate HJ, Marks GC, Durham J: Cash transfer programmes in lower-income and middle-income countries: understanding pathways to nutritional change-a realist review protocol. *BMJ Open*. 2019;9:28314. doi:10.1136/bmjopen-2018-028314 75

3. Souza D, Chmielewska D: Public Support To Food Security in India, Brazil and South Africa: Elements for a Policy Dialogue. 2011. https://ipcig.org/pub/IPCWorkingPaper80.pdf. 76

4. Neufeld: Nutrition in the Oportunidades conditional cash transfer program: Strengths and challenges, presentation at the Third International CCT Conference, Istanbul, 29 June 2006. 77

5. World Bank: “Managing Risk, Promoting Growth: Developing Systems for Social Protection in Africa—Africa Social Protection Strategy 2011–2021.” Concept Note, World Bank, Washington, DC. 2011. 77

6. Narayanan S, Gerber N: Social safety nets for food and nutrition security in India. *Glob Food Sec*. 2017;15:65-76. doi:10.1016/j.gfs.2017.05.001 78

7. Leroy JL, Ruel M, Verhofstadt E: The impact of conditional cash transfer programmes on child nutrition: a review of evidence using a programme theory framework. *J Dev Eff*. 2009;1(2):103-129. doi:10.1080/19439340902924043 79

8. de Groot R, Palermo T, Handa S, Ragno LP, Peterman A: Cash Transfers and Child Nutrition: Pathways and Impacts. *Development Policy Review* 2017, 35(5):621-643. doi:10.1111/dpr.12255 80

9. Alderman H: Leveraging Social Protection Programs for Improved Nutrition: Summary of Evidence Prepared for the Global Forum on Nutrition-Sensitive Social Protection Programs, 2015. Washington, D.C.; 2016. Available from: http://www.securenutrition.org/sites/default/files/resources/attachment/english/Alderman - Global Forum Summary of Evidence - 2016_0.pdf. 81

10. Olney DK, Marshall Q, Honton G, et al: Leveraging an Implementation-Research Partnership to Improve Effectiveness of Nutrition-Sensitive Programs at the World Food Programme. *Food Nutr Bull*. 2019. doi:10.1177/0379572119874273 82

General Nutrition

1. United Nations Children's Fund: **UNICEF's approach to scaling up nutrition for mothers and their children.** In. Edited by Programme Division. New York: UNICEF; 2015.

FIGURE 1

UNICEF CONCEPTUAL FRAMEWORK OF THE DETERMINANTS OF CHILD UNDERNUTRITION

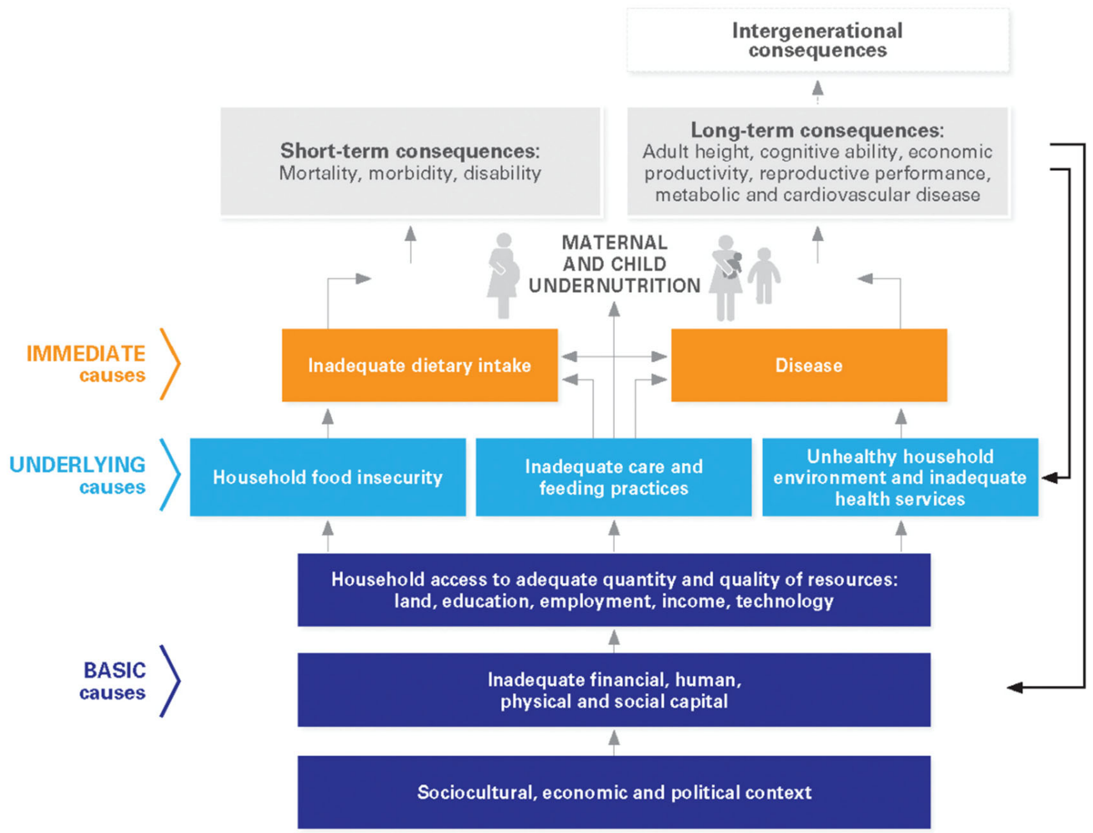

The black arrows show that the consequences of undernutrition can feed back to the underlying and basic causes of undernutrition, perpetuating the cycle of undernutrition, poverty and inequities.

Source: Adapted from UNICEF, 1990.

2. SPRING: A Systems Thinking and Action for Nutrition: A Working Paper. In. Edited by USAID/ Strengthening Partnerships R, and Innovations in Nutrition Globally (SPRING) Project. Arlington, VA; 2015.

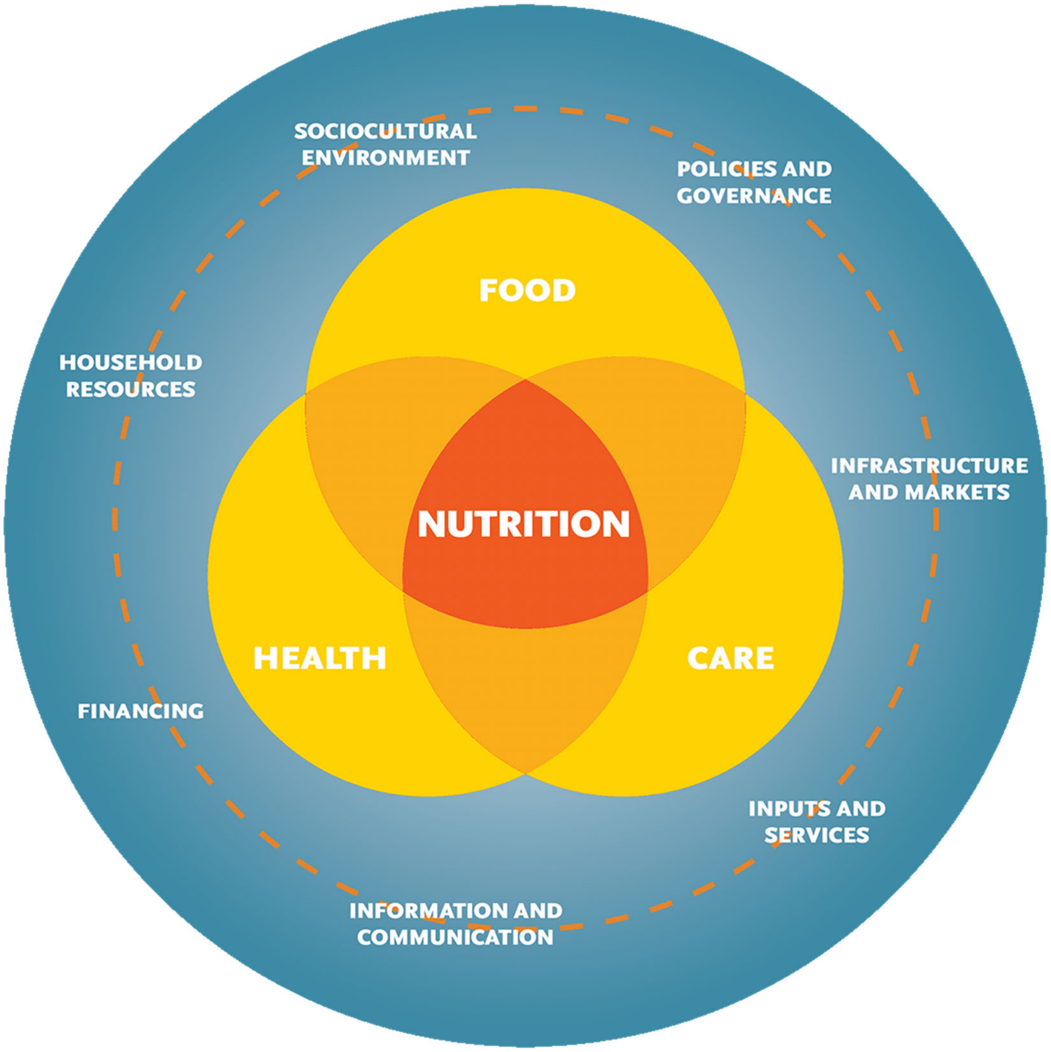

3. Black RE, Victora CG, Walker SP, Bhutta ZA, Christian P, de Onis M, Ezzati M, Grantham-McGregor S, Katz J, Martorell R et al: Maternal and child undernutrition and overweight in low-income and middle-income countries. Lancet (London, England) 2013, 382(9890):427-451.

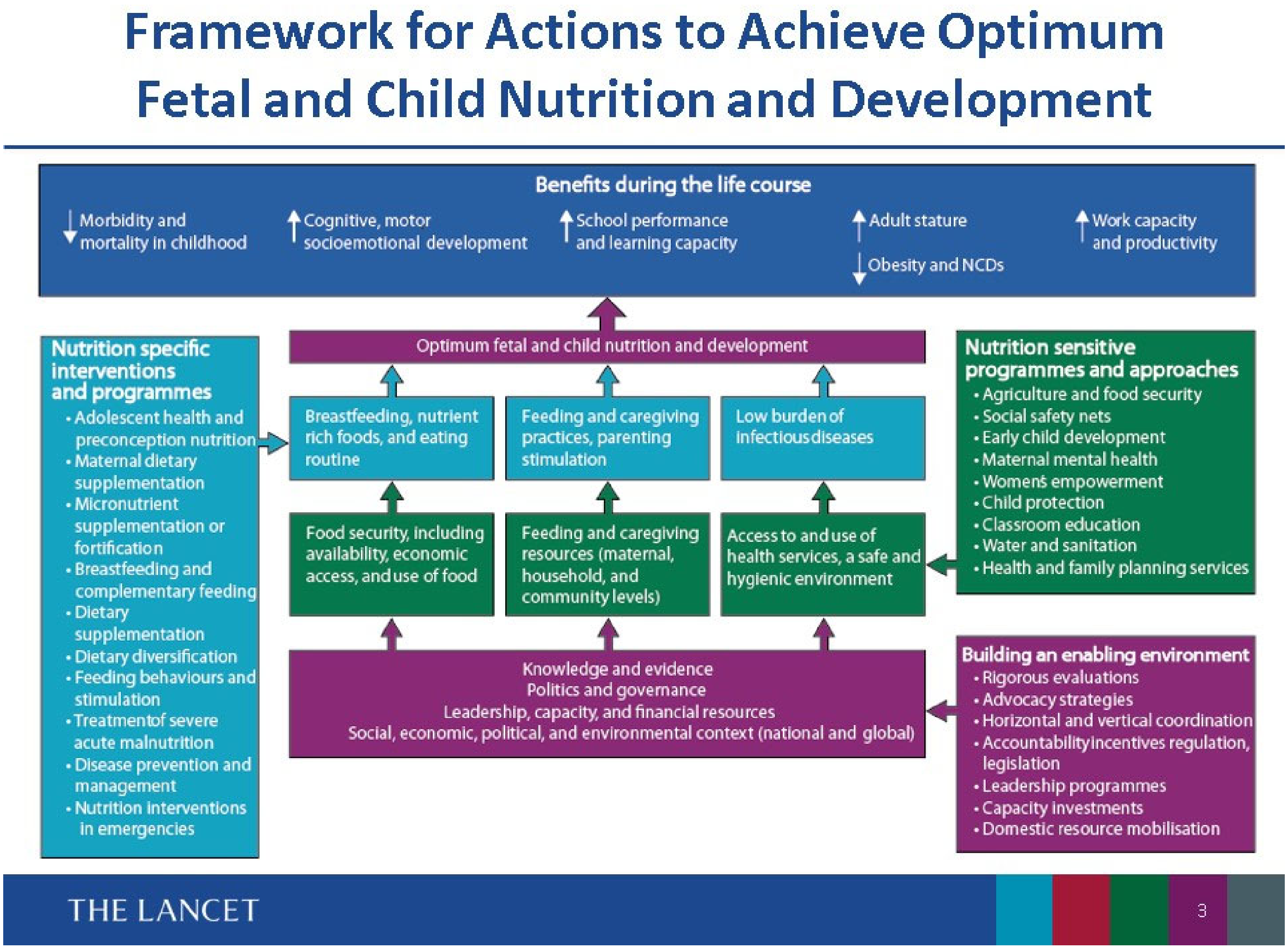

4. World Bank: Improving Nutrition Through Multisectoral Approaches. Edited by World Bank. Washington, DC; 2013 2013. https://openknowledge.worldbank.org/handle/10986/16450 License: CC BY-NC-ND 3.0 IGO.

FIGURE E-1. HEALTH SECTOR-SPECIFIC FRAMEWORK FOR CHILD NUTRITION

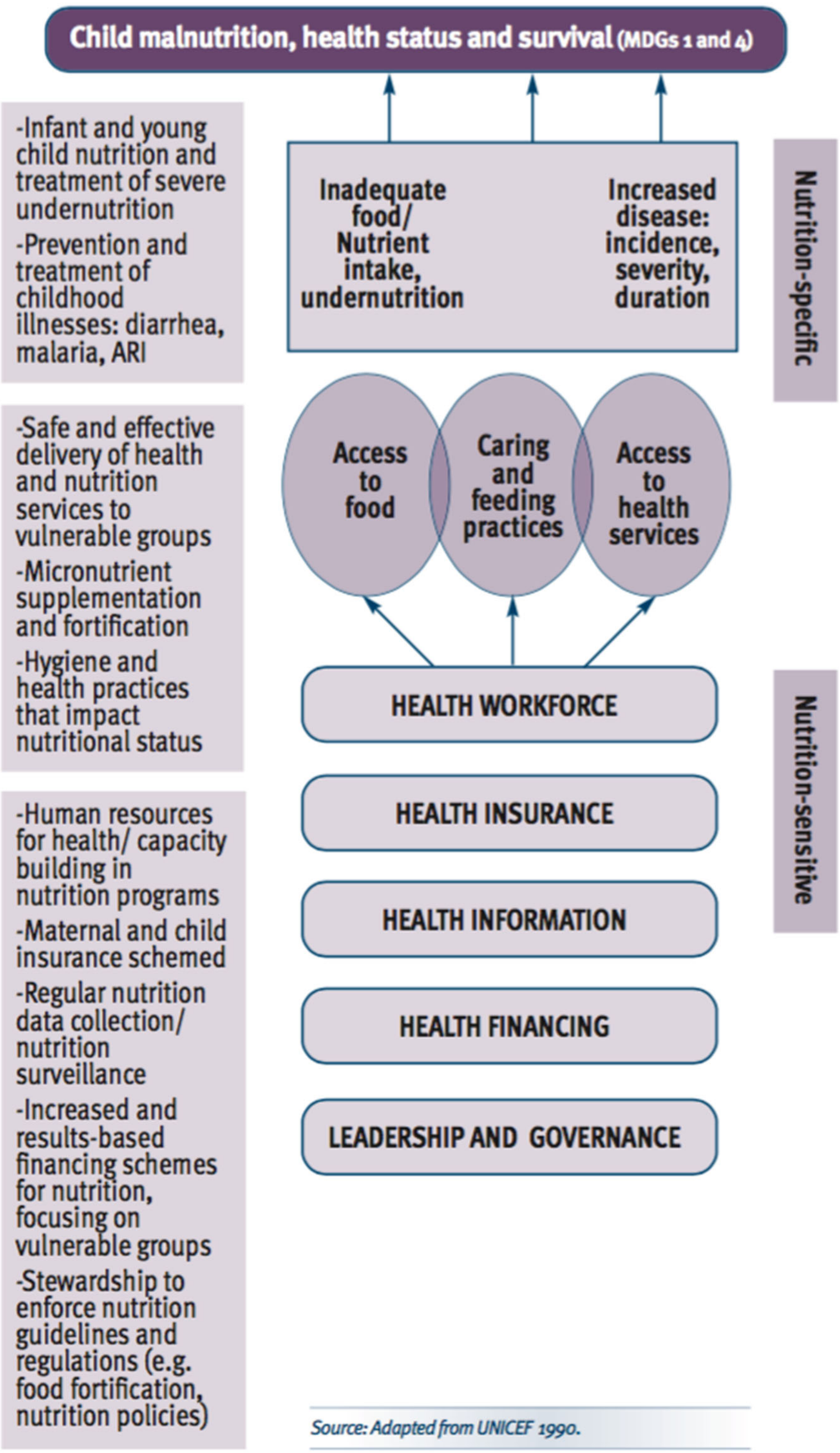

Source: Adapted from UNICEF 1990.

Health System

1. Londoño JL, Frenk J: **Structured pluralism: towards an innovative model for health system reform in Latin America**. *Health policy (Amsterdam, Netherlands)* 1997, 41(1):1-36.

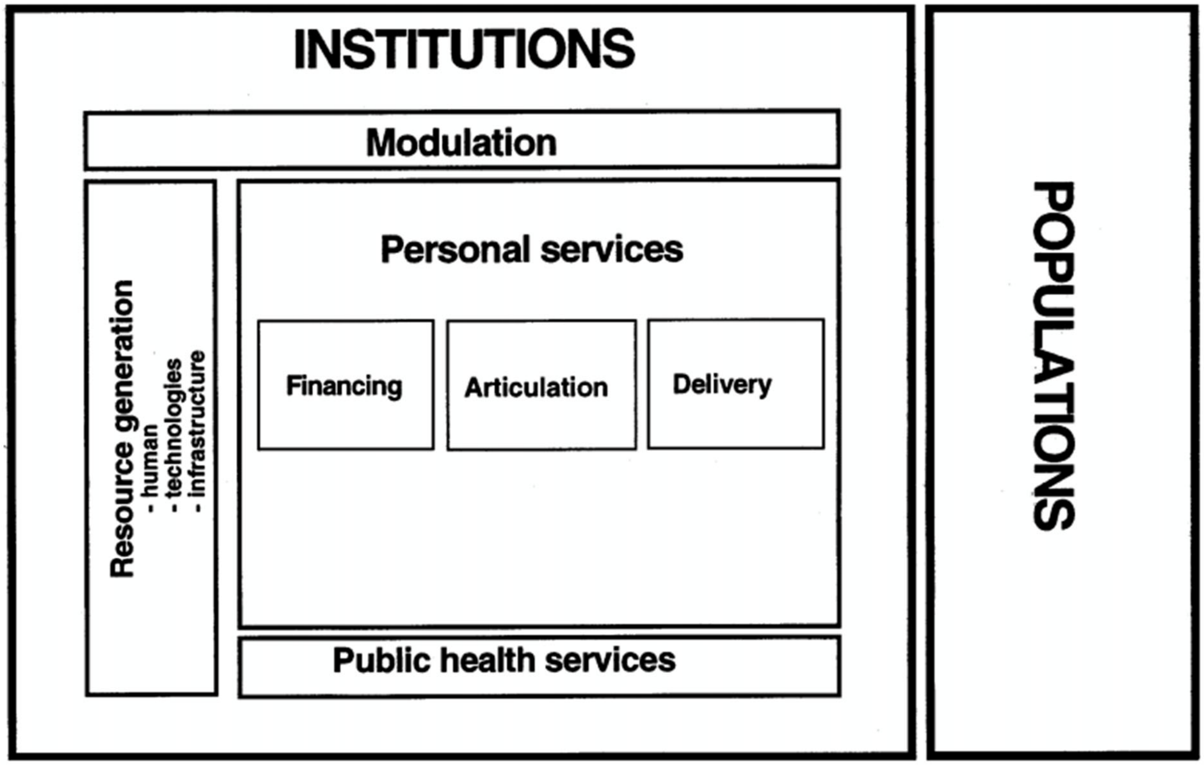

Fig. 2. Components of health systems.

2. World Health Organization: **Everybody's business -- strengthening health systems to improve health outcomes : WHO's framework for action.** In. Edited by WHO. Geneva, Switzerland; 2007. https://apps.who.int/iris/handle/10665/43918

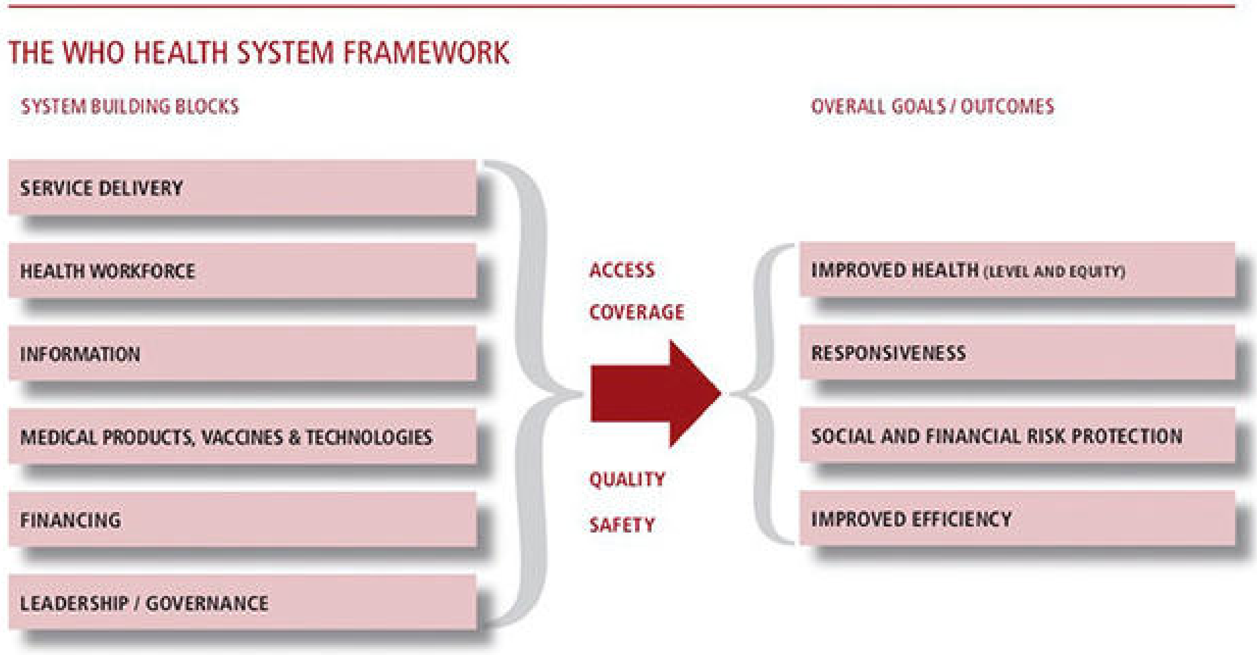

3. World Health Organization: **The World Health Report 2000**. In: *Improving Performance.* Edited by WHO. Geneva, Switzerland; 2000

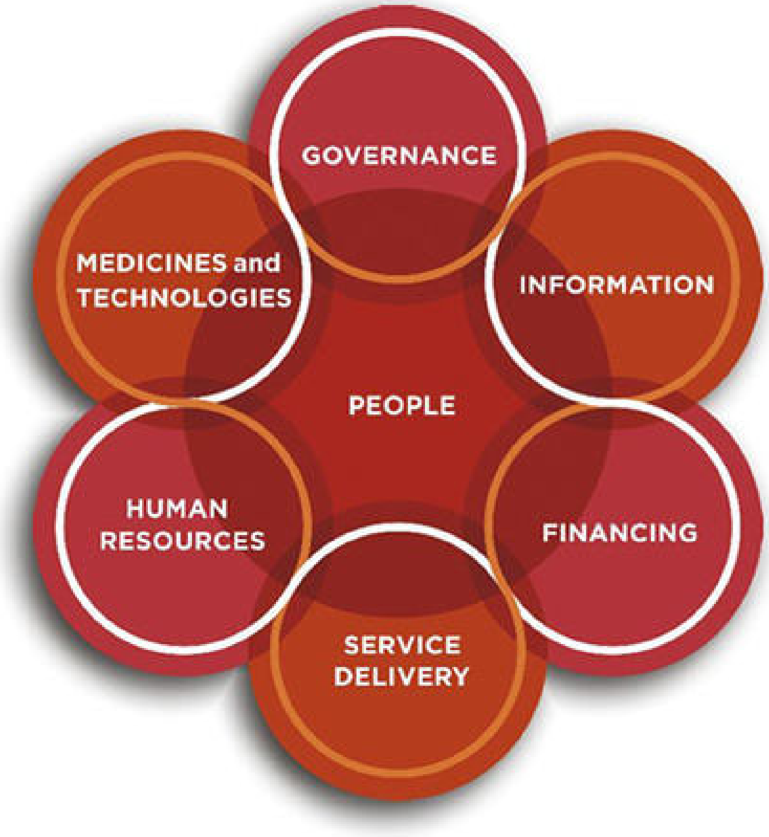

5. World Bank: Improving Nutrition Through Multisectoral Approaches. Edited by World Bank. Washington, DC; 2013 2013. https://openknowledge.worldbank.org/handle/10986/16450 License: CC BY-NC-ND 3.0 IGO.

FIGURE A-3. DETERMINANTS OF CHILD NUTRITION AND INTERVENTIONS TO ADDRESS THEM

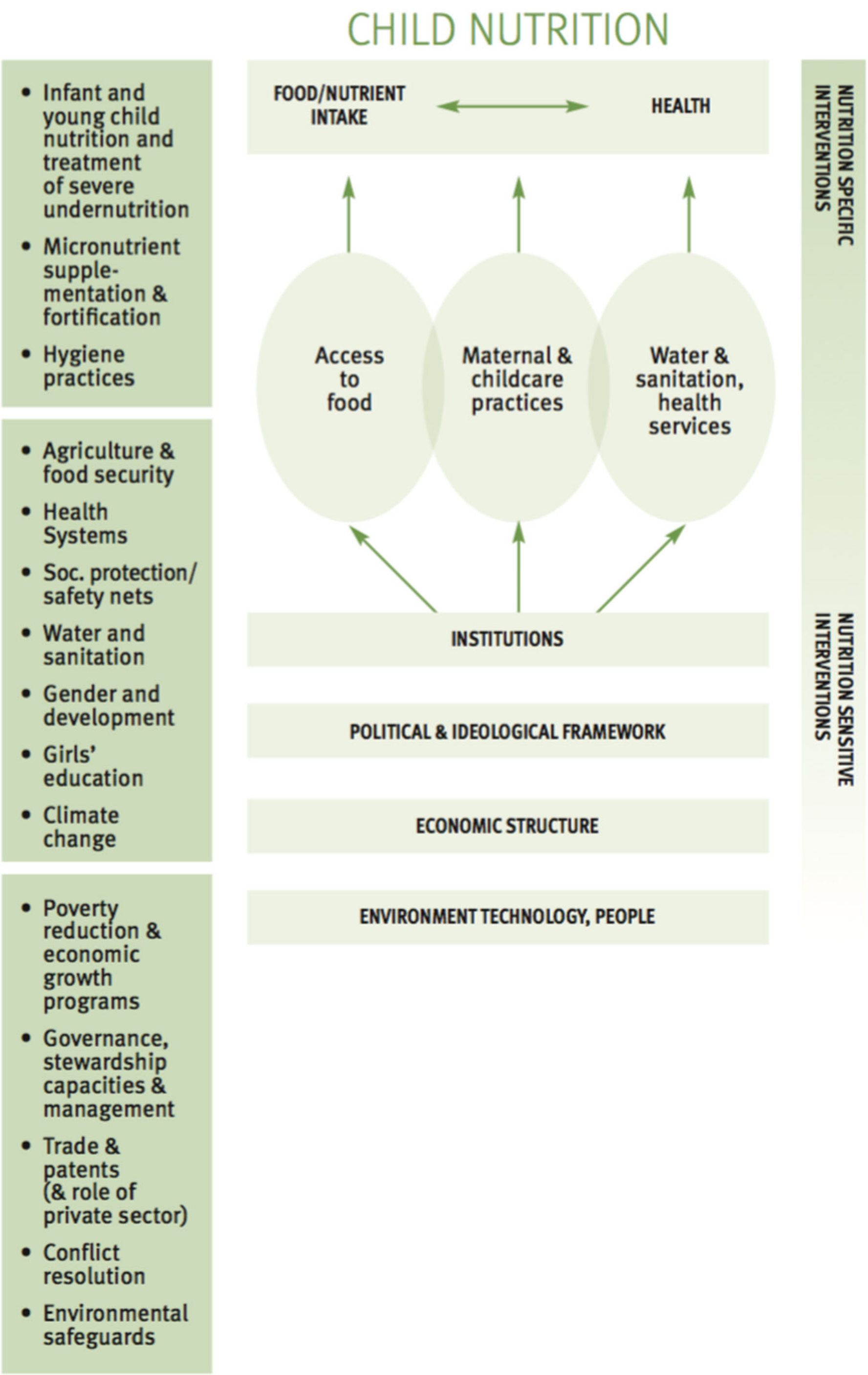

Source: Adapted from UNICEF 1990 and Ruel 2008.

4. Roberts M, Hsiao W, Berman P, Reich M: **Getting Health Reform Right – A Guide to Improving Performance and Equity.** New York; 2003

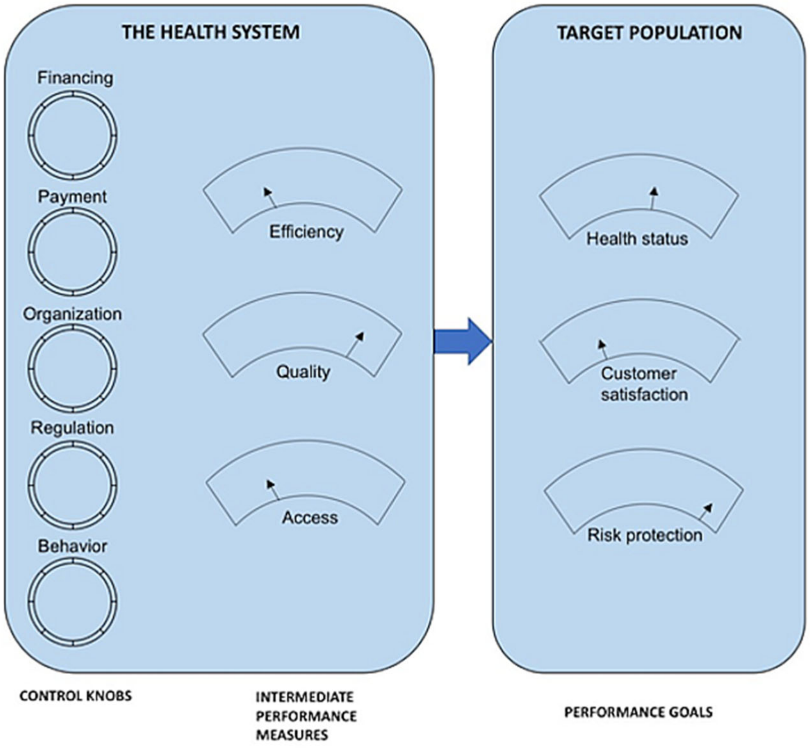

5. Atun R, Menabde N: **Health systems and systems thinking:** Open University Press; 2008

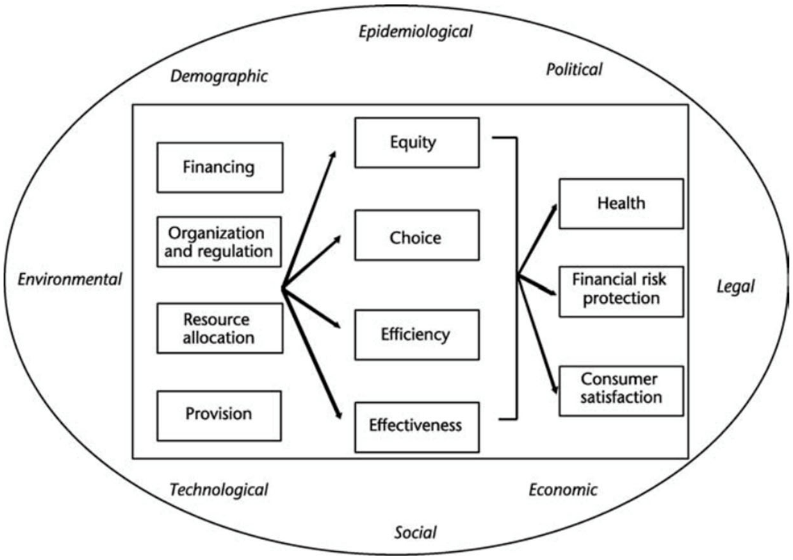

Figure 7.1 A framework for analysing health systems and the context

Note: Framework adapted from Atun, R. A., Kyratsis, I., Gurol, I., Rados-Malicbegovic, R. and Jelic, G. (2007c). Diffusion of complex health innovations-implementation of primary care reforms in Bosnia and Herzegovina: a qualitative study, Health Policy and Planning, 22(1): 28–39.

6. Kleczkowski B, Roemer M, Van Der Werff A: National health systems and their reorientation towards health for all. Guidance for policy-making. 77th edition. Geneva: World Health Organisation; 1984.

Fig. 1. Model of a national health system: its structure and functional interrelationships

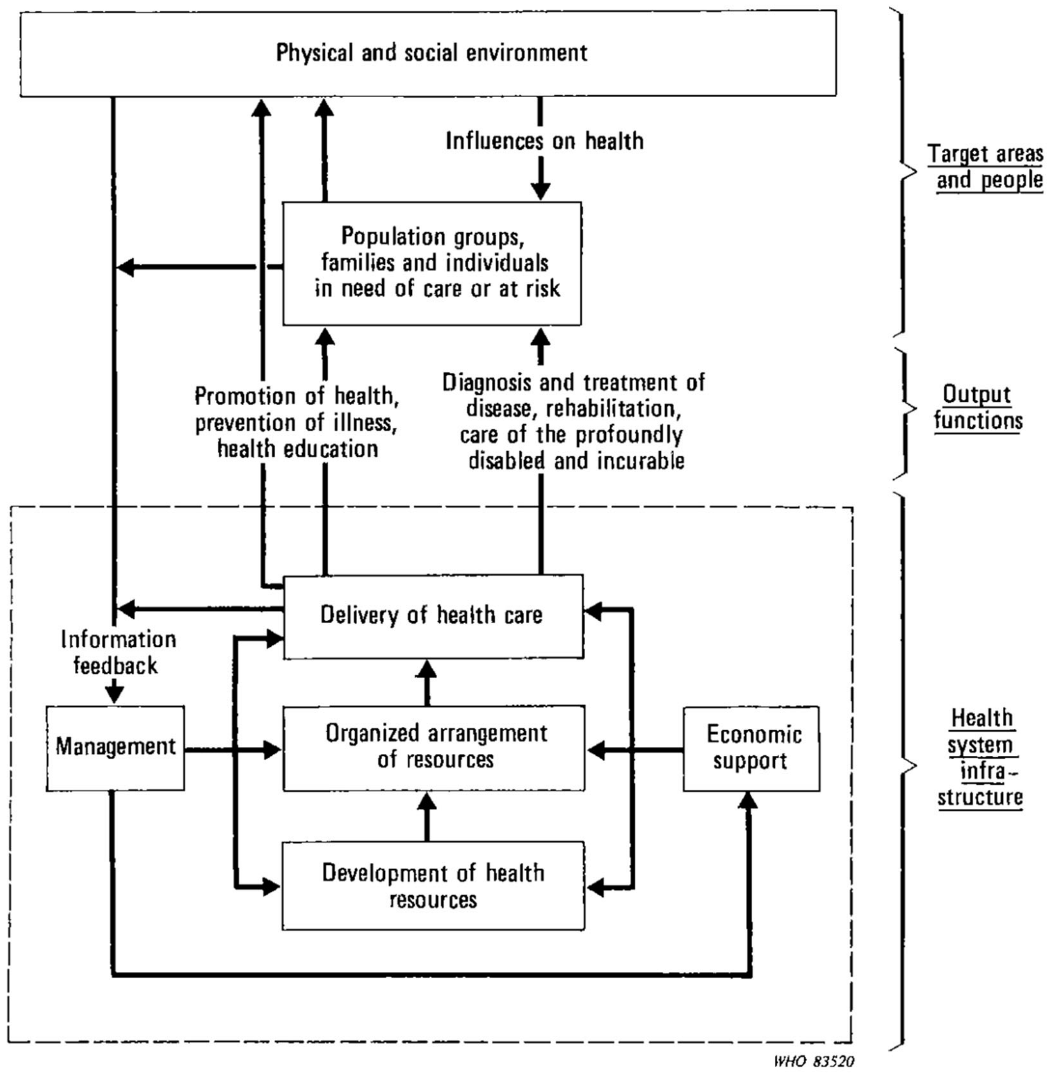

7. World Health Organization: **The World Health Report 2000**. In: *Improving Performance.* Edited by WHO. Geneva, Switzerland; 2000

Figure 2.1 Relations between functions and objectives of a health system

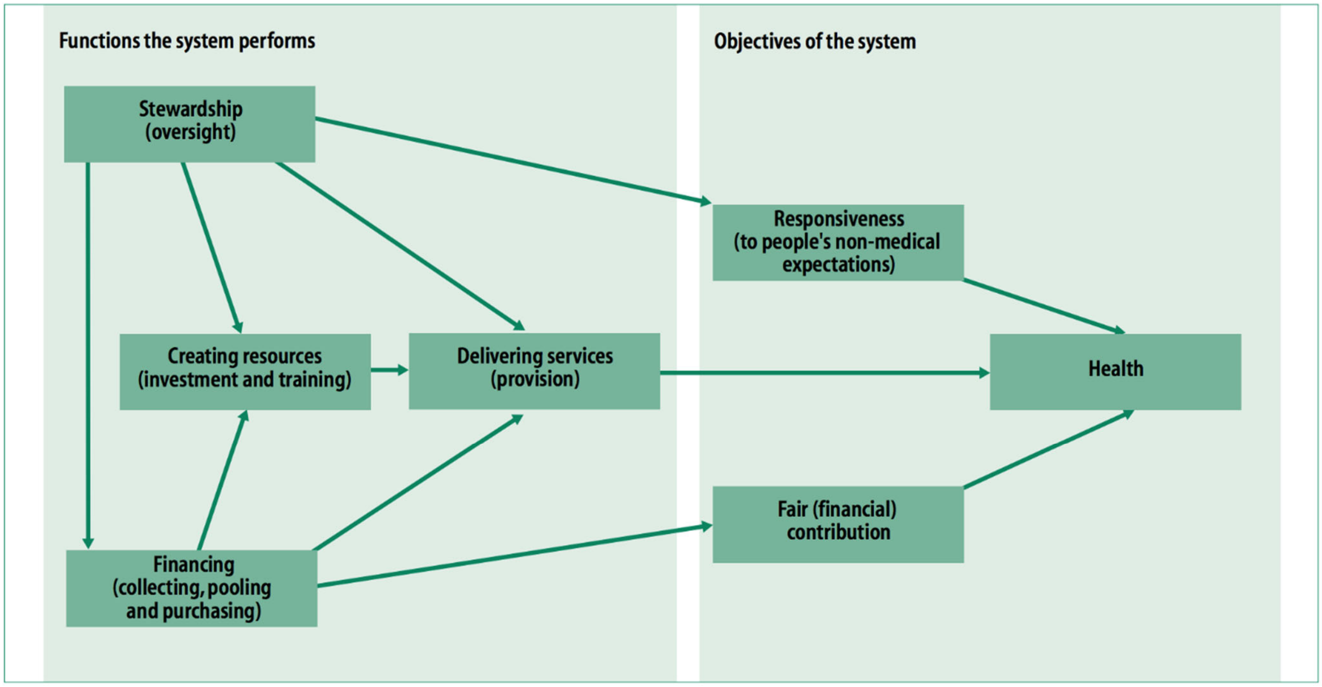

8. Sacks E, Morrow M, Story WT, Shelley KD, Shanklin D, Rahimtoola M, Rosales A, Ibe O, Sarriot E: **Beyond the building blocks: integrating community roles into health systems frameworks to achieve health for all.** 2019, 3(Suppl 3):e001384.

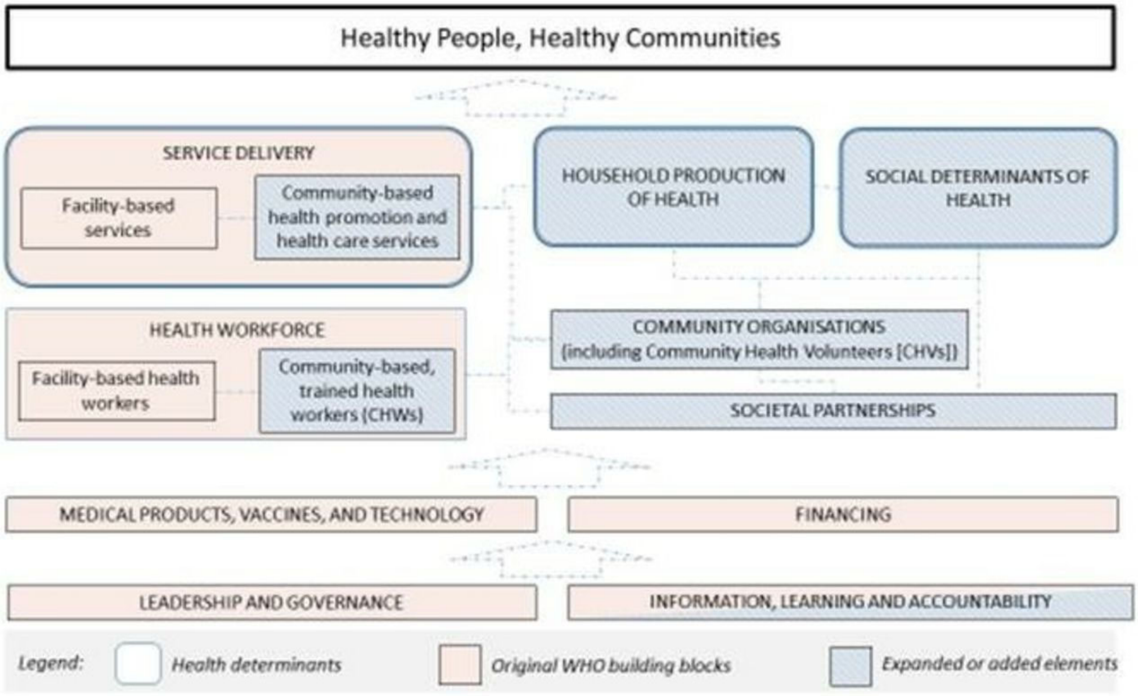

Food and Agriculture

1. Sobal J, Kettel Khan L, Bisogni C: A conceptual model of the food and nutrition system. *Social Science & Medicine* 1998, 47(7):853-863. https://doi.org/10.1016/S0277-9536(98)00104-X

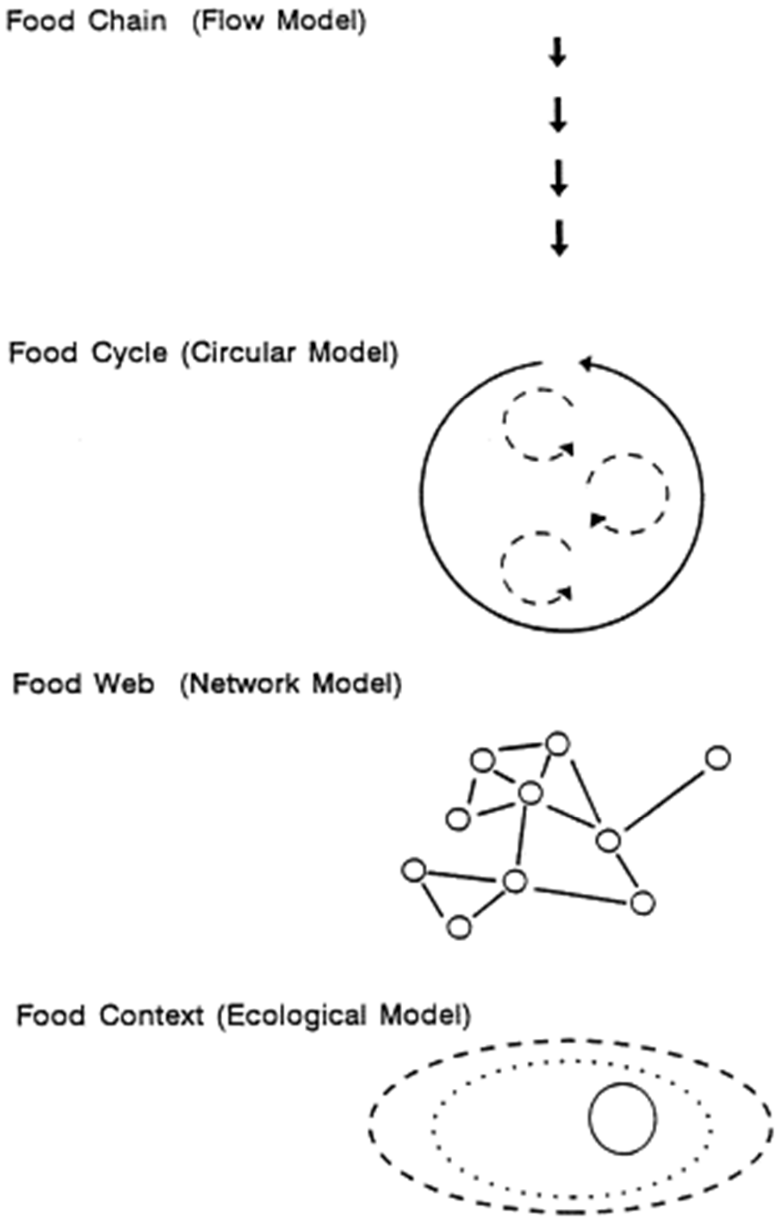

Continued: Sobal J, Kettel Khan L, Bisogni C: A conceptual model of the food and nutrition system. Social Science & Medicine 1998, 47(7):853-863. https://doi.org/10.1016/S0277-9536(98)00104-X

THE FOOD AND NUTRITION SYSTEM

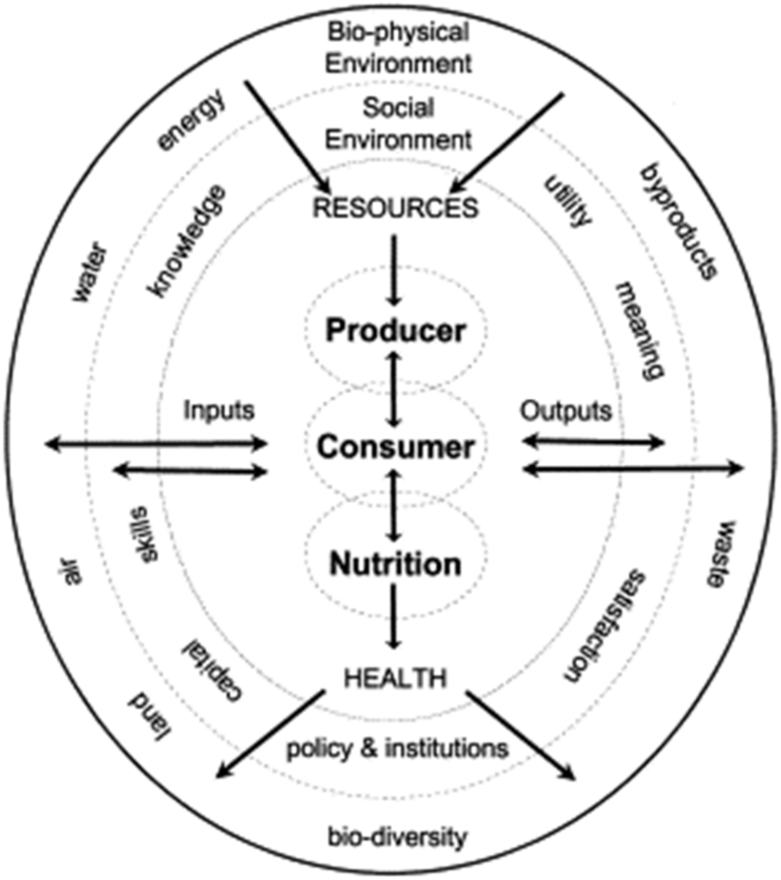

SELECTED ROLES AND RELATIONSHIPS IN THE FOOD AND NUTRITION WEB

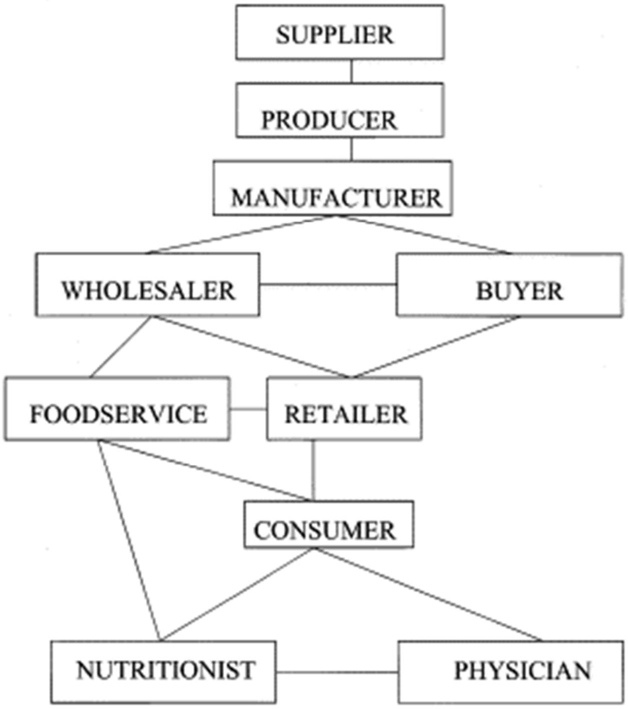

Relationships of the Food and Nutrition System to Selected Other Systems

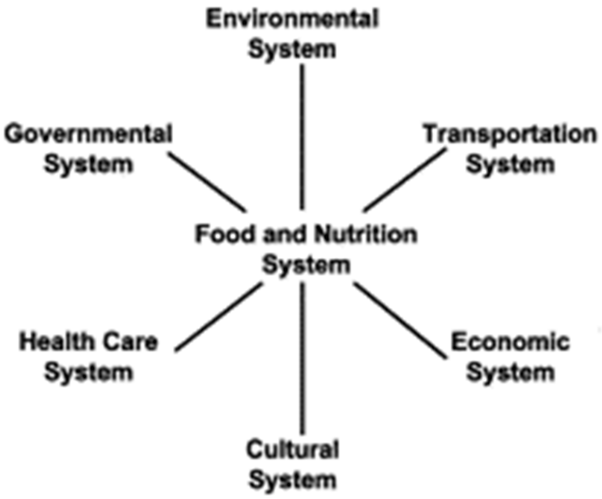

2. Chung K: An Introduction to Nutrition-Agriculture Linkages. Direccao de economia, Ministerio da agricultura, Republica de Mocambique 2012. https://www.spring-nutrition.org/sites/default/files/chung_msu_2012_ag-nutrition_linkages.pdf

Fig 1: Ag-Nutrition Linkages at the Household and Intra-Household Levels

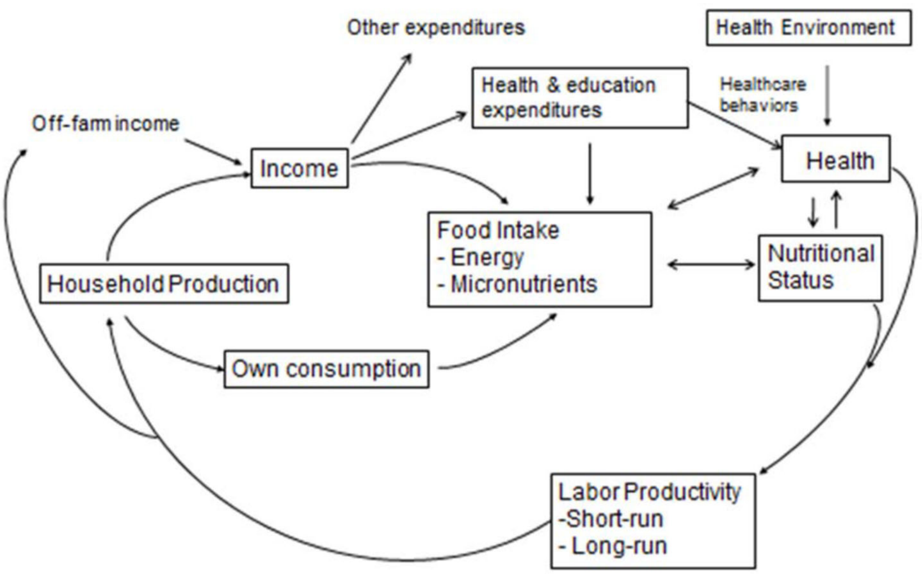

Figure 2: The Trickle Down Approach to Improving Nutrition thru Agriculture

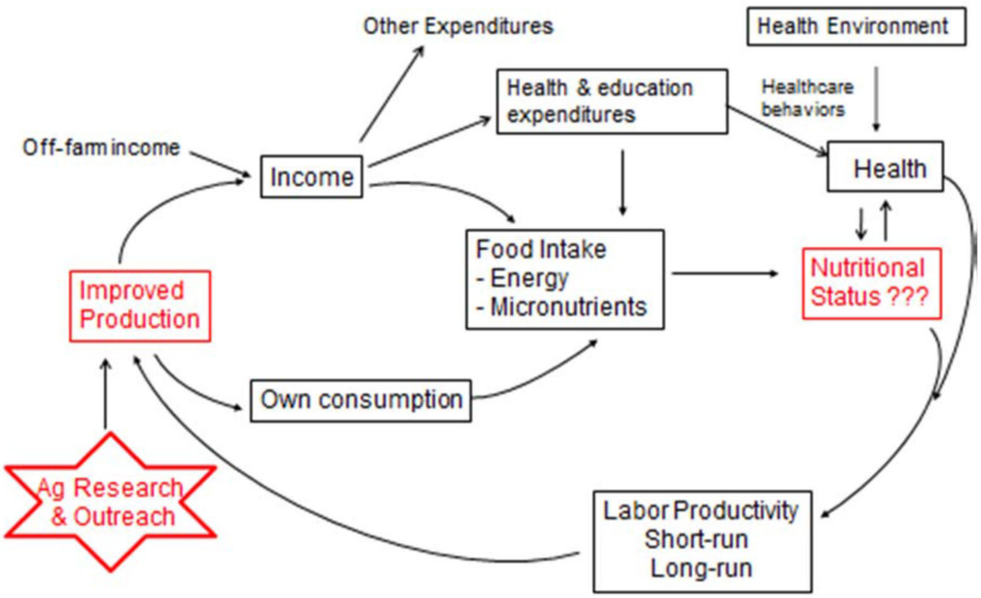

Continued: Chung K: An Introduction to Nutrition-Agriculture Linkages. Direccao de economia, Ministerio da agricultura, Republica de Mocambique 2012. https://www.spring-nutrition.org/sites/default/files/chung_msu_2012_ag-nutrition_linkages.pdf

Figure 3: The Biofortification Approach

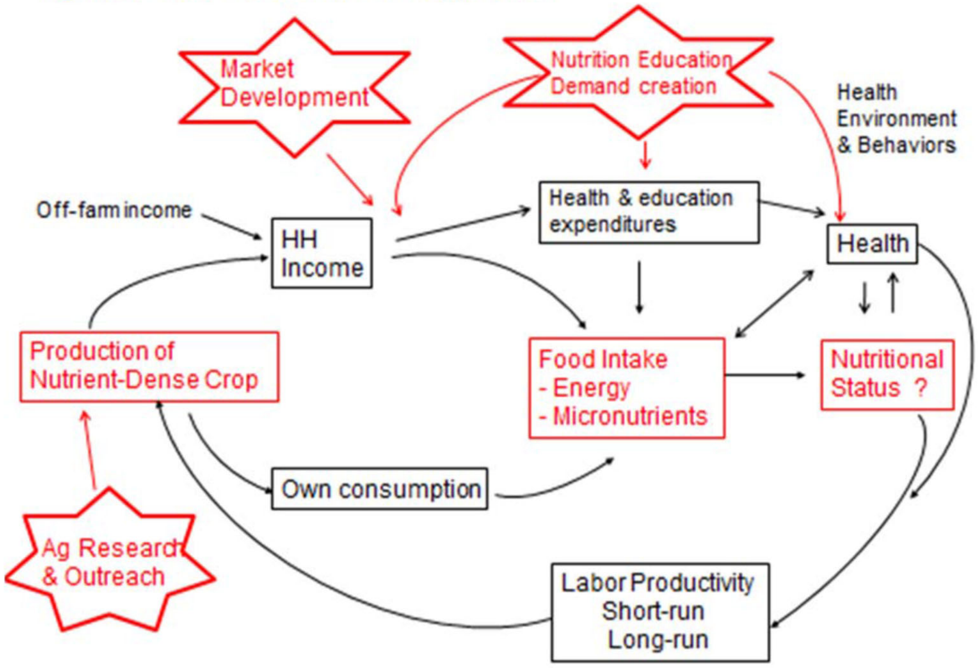

Figure 4: The Ag-Based Diet Diversity Approach

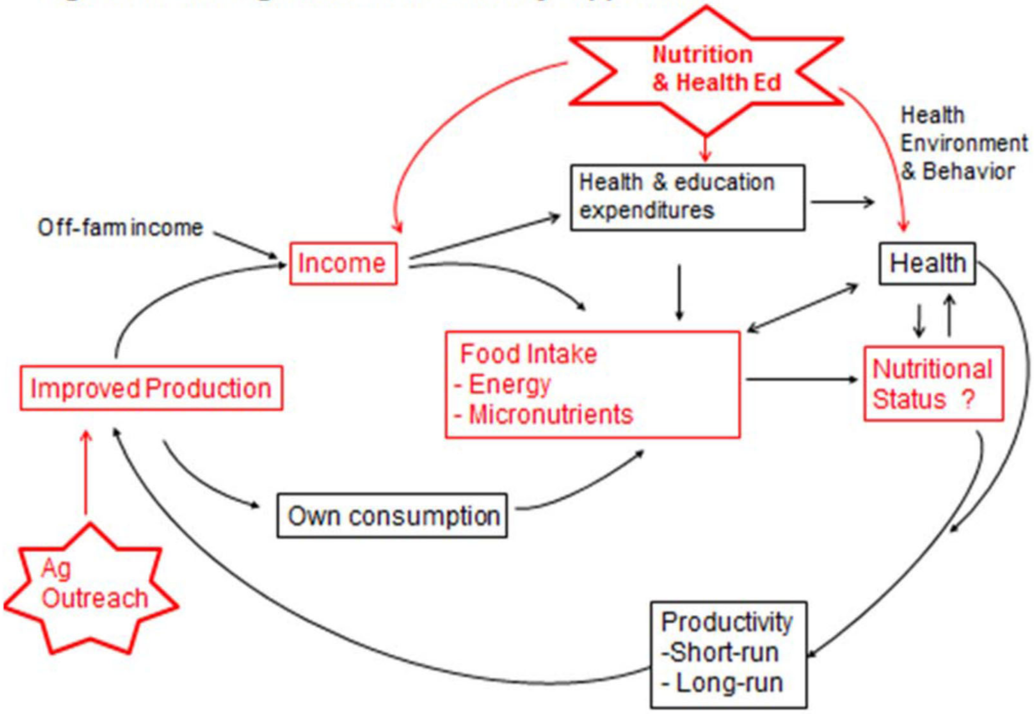

Continued: Chung K: An Introduction to Nutrition-Agriculture Linkages. Direccao de economia, Ministerio da agricultura, Republica de Mocambique 2012. https://www.spring-nutrition.org/sites/default/files/chung_msu_2012_ag-nutrition_linkages.pdf

Figure 5: A Gendered View of Nutrition-Agriculture Linkages

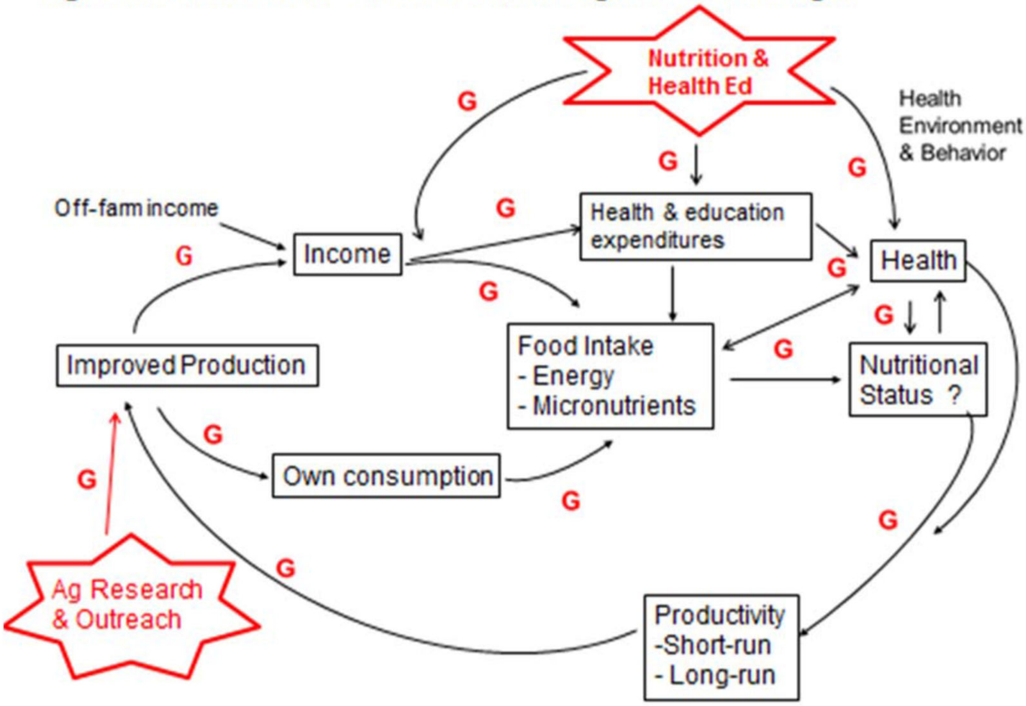

3. Kennedy E, Bouis HE: Linkages between agriculture and nutrition: implications for policy and research. Washington, DC: International Food Policy Research Institute; 1993. http://ebrary.ifpri.org/utils/getfile/collection/p15738coll2/id/125519/filename/125550.pdf

Figure 1—Agricultural policies, household resource allocation, and nutrition

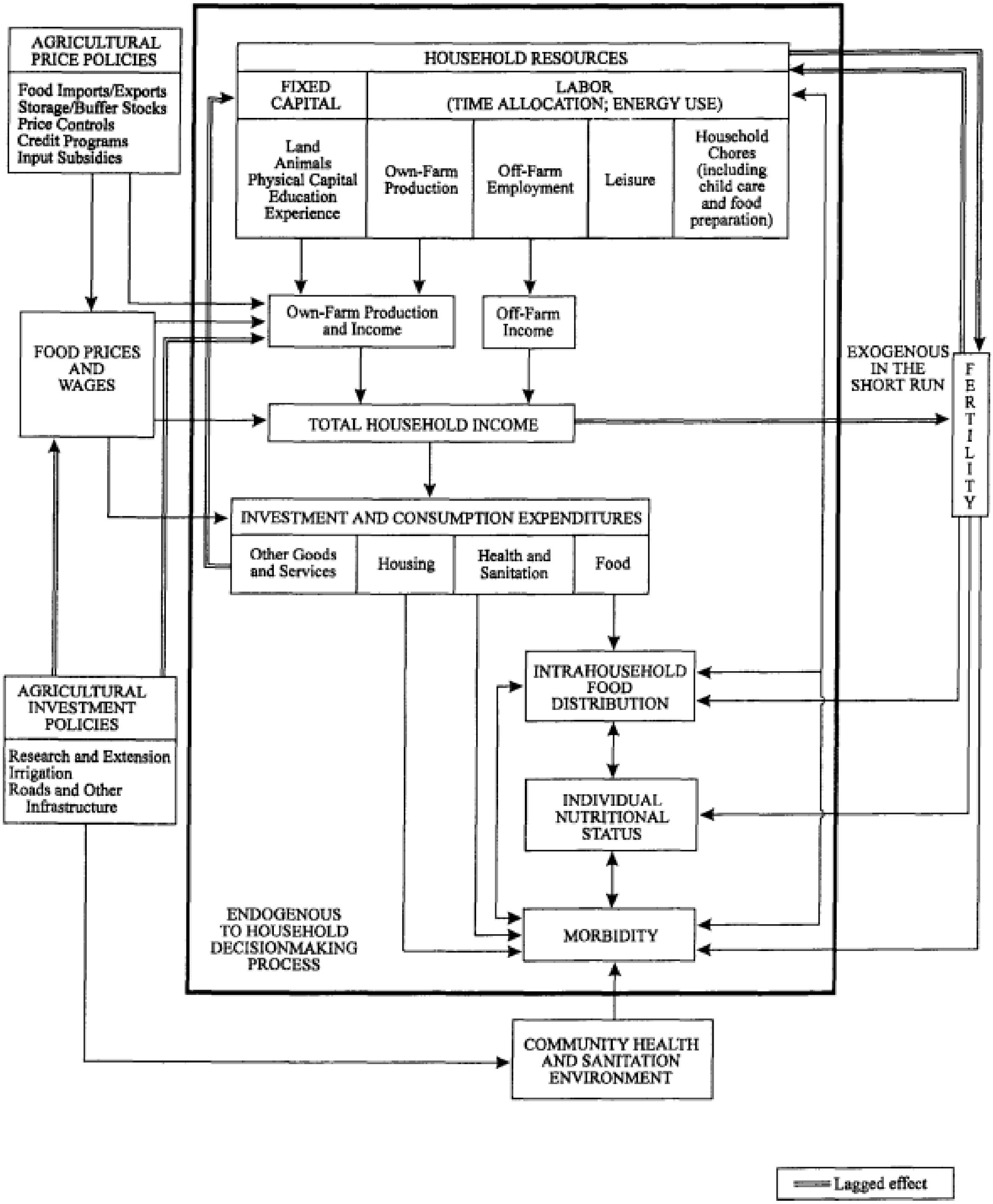

4. Hawkes C: Identifying Innovative Interventions to Promote Healthy Eating Using Consumption-Oriented Food Supply Chain Analysis. *J Hunger Environ Nutr* 2009, 4(3-4):336-356. https://www.tandfonline.com/doi/full/10.1080/19320240903321243

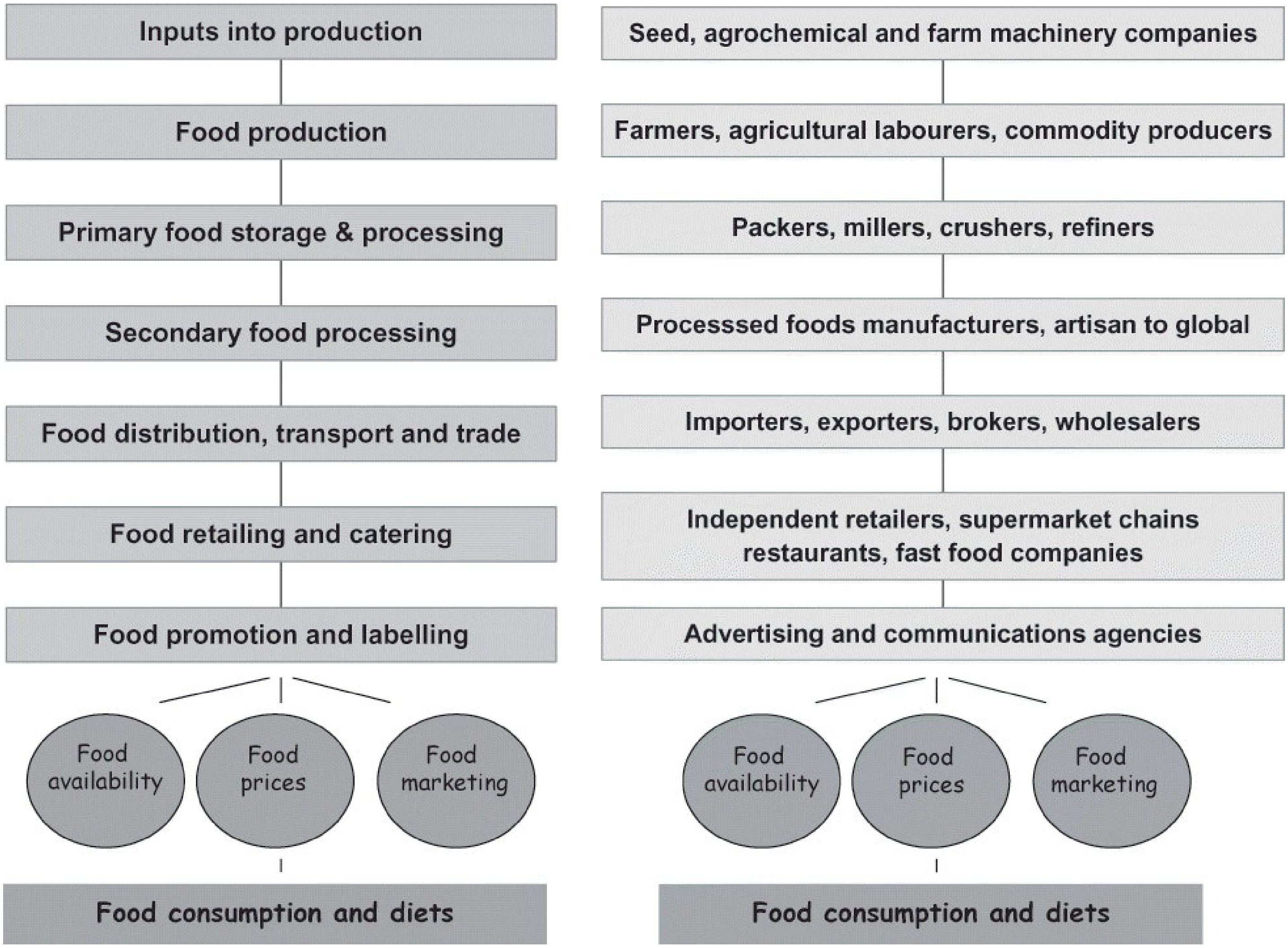

Continued: Hawkes C: **Identifying Innovative Interventions to Promote Healthy Eating Using Consumption-Oriented Food Supply Chain Analysis.** *J Hunger Environ Nutr* 2009, 4(3-4):336-356. https://www.tandfonline.com/doi/full/10.1080/19320240903321243

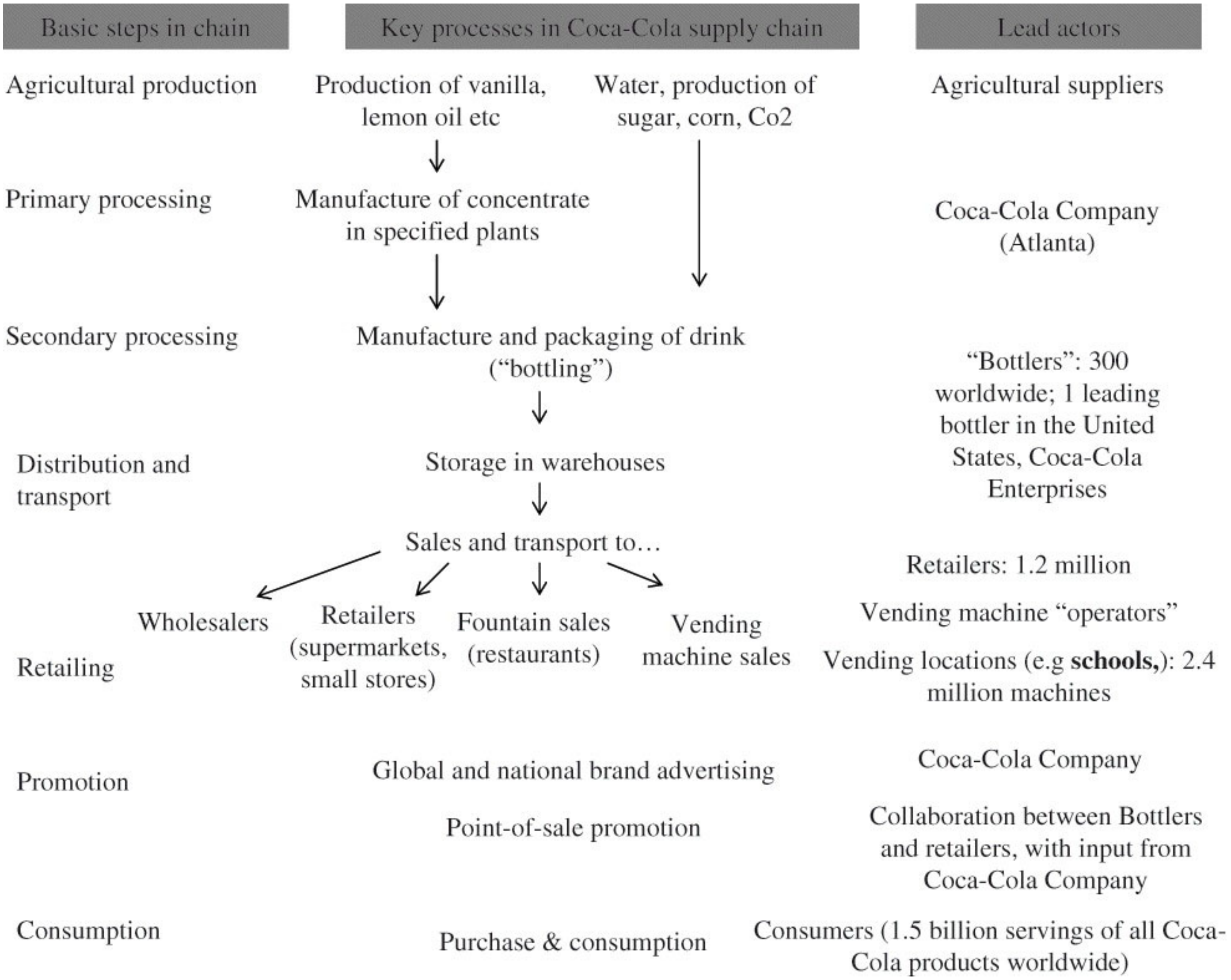

5. Kanter R, Walls HL, Tak M, Roberts F, Waage J. A conceptual framework for understanding the impacts of agriculture and food system policies on nutrition and health. 2015. https://link.springer.com/article/10.1007%2Fs12571-015-0473-6

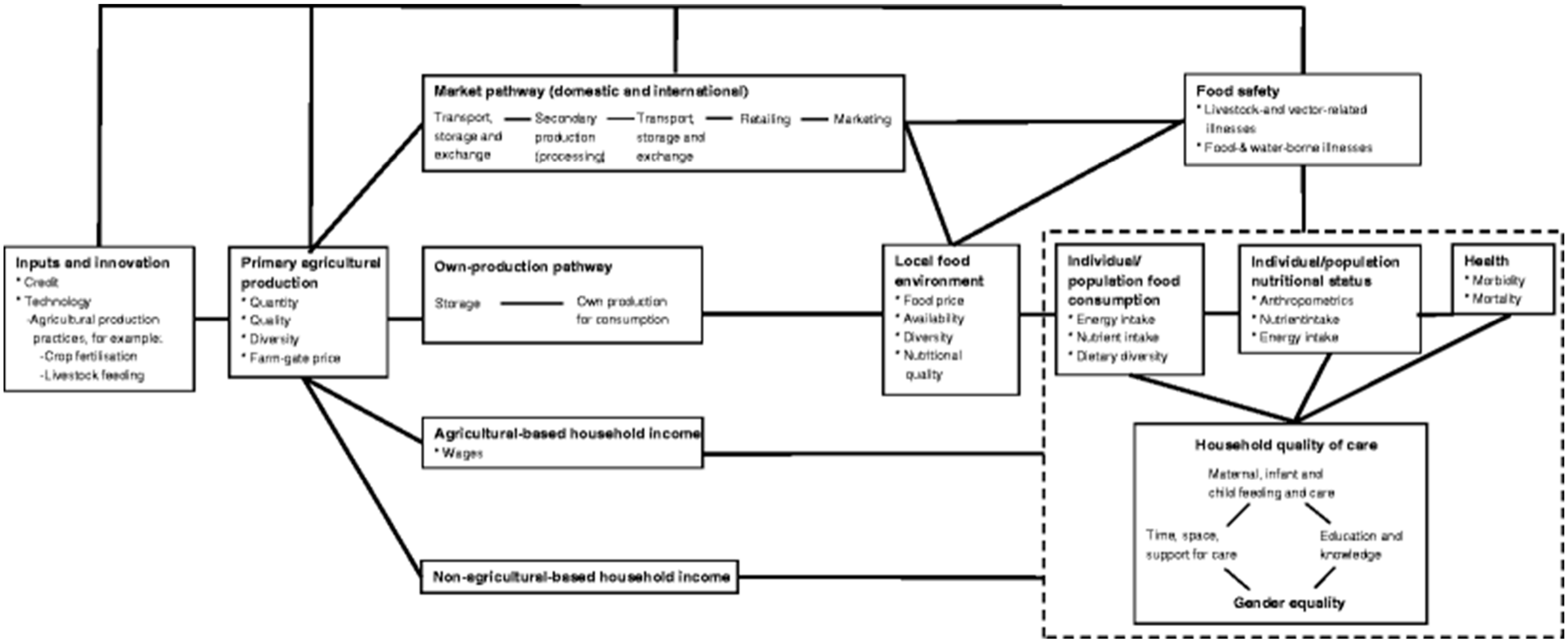

Note: Conceptual framework of the links between agriculture, the food system, nutrition and public health. Note that we have not included other important influences acting at multiple points across the framework, including, but not limited to: culture; gender and gender inequality; weather and climate variability; political and economic circumstances.

Continued: Kanter R, Walls HL, Tak M, Roberts F, Waage J. **A conceptual framework for understanding the impacts of agriculture and food system policies on nutrition and health.** 2015. https://link.springer.com/article/10.1007%2Fs12571-015-0473-6

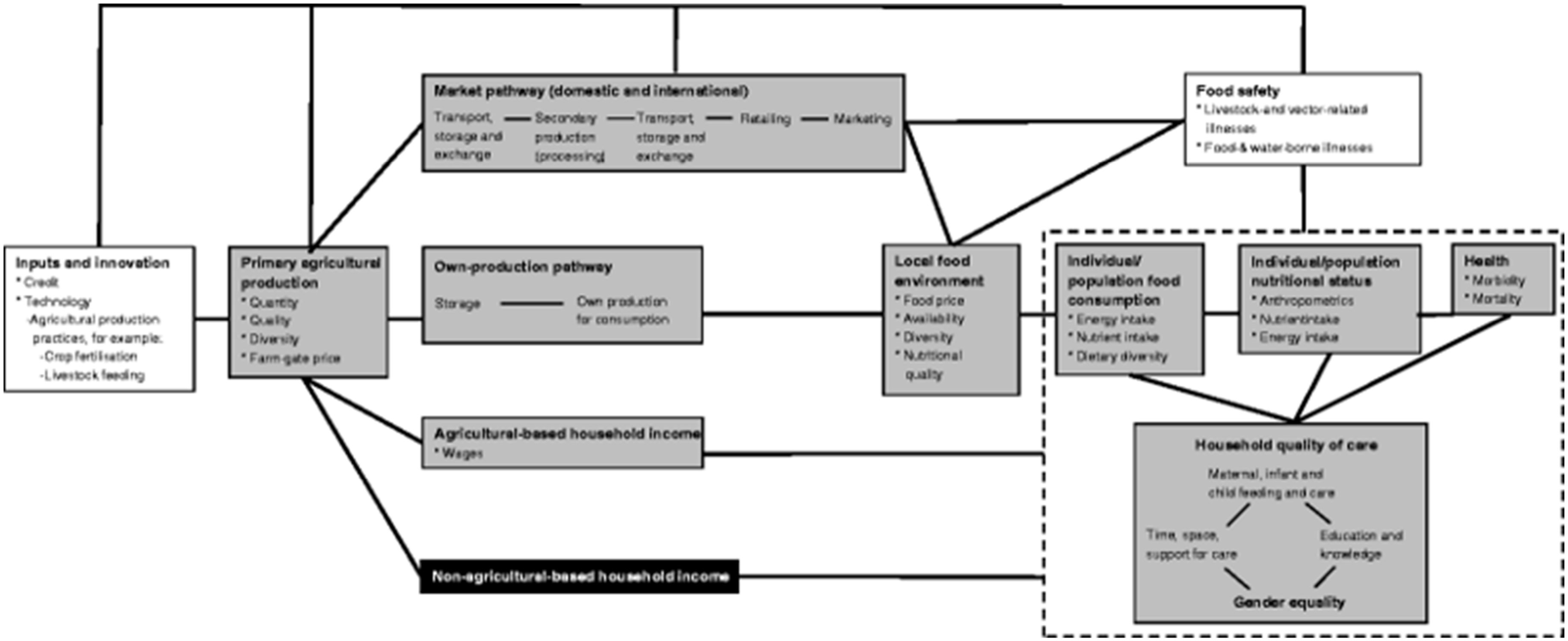

Note: Conceptual framework of the links between agriculture, the food system, nutrition and public health. An example of a public distribution system policy, a conditional cash transfer program, and the direct and indirect impacts of that policy on agriculture, the food system, nutrition and public health (*Black box* with *white* text indicates 'starting point' for a conditional cash transfer policy. *Grey boxes* indicate where a conditional cash transfer policy would likely have direct impacts, while the *boxes* in *white* are where a conditional cash transfer policy may have indirect impacts). Note that we have not included other important influences acting at multiple points across the framework, including, but not limited to: culture; gender and gender inequality; weather and climate variability; political and economic circumstances.

6. Gillespie, S; Harris, J; Kadiyala, S; (2012) **The Agriculture-Nutrition Disconnect in India: What Do We Know?** Washington, DC; 2012. https://researchonline.lshtm.ac.uk/id/eprint/1440425

Figure 3.1—Mapping the agriculture-nutrition disconnect

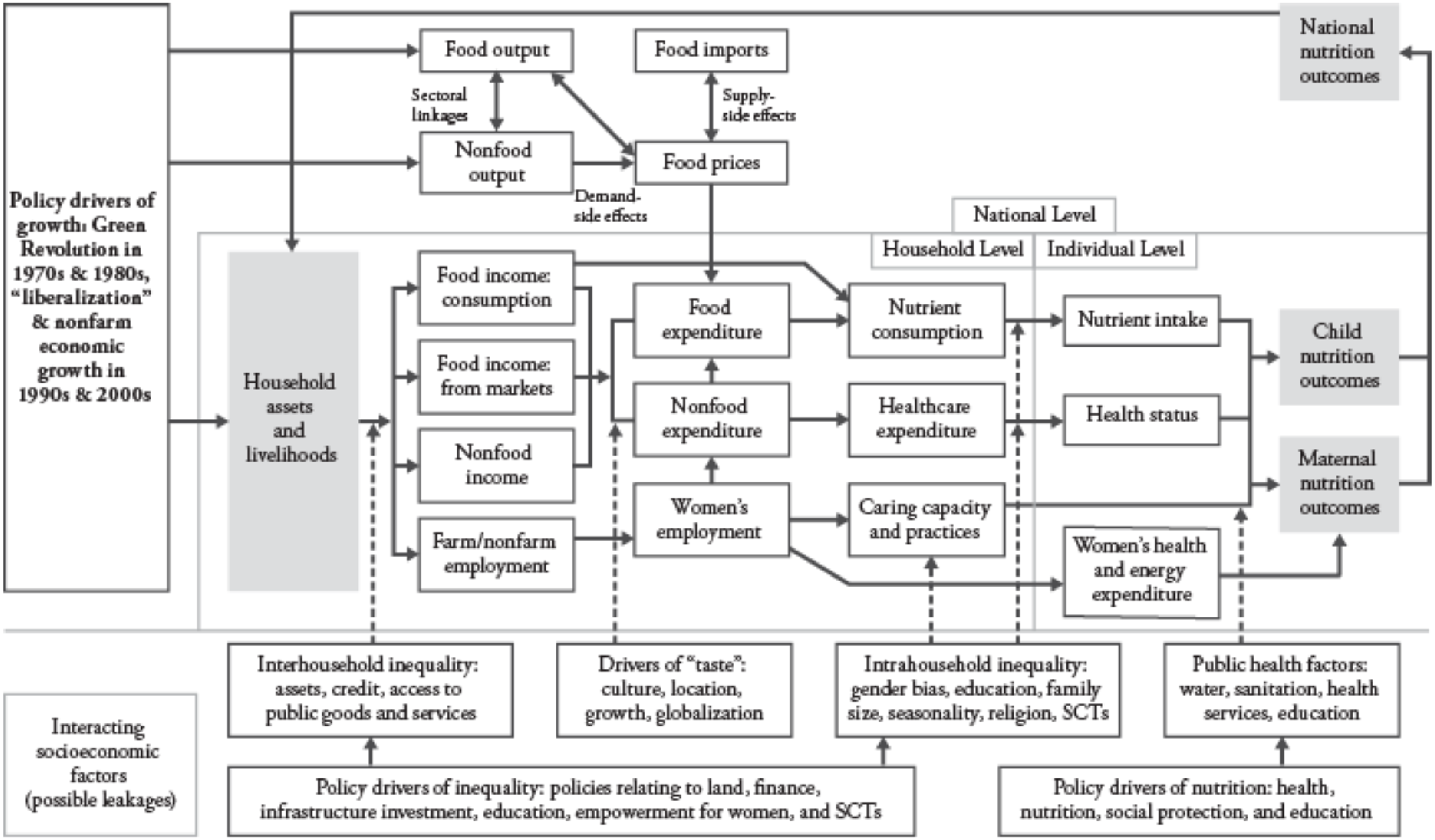

Source: Adapted by the authors from Headey, Chiu, and Kadiyala (2011).

7. Food and Agriculture Organization of the United Nations Rome: **Compendium of nutrition-sensitive indicators in agriculture.** Rome, Italy; 2016

Figure 1. Simplified impact pathway framework of investment projects. This framework identifies six outcome areas that are directly affected by agriculture, rural development and food systems, and how these can influence nutrition (see glossary of terms, page10).

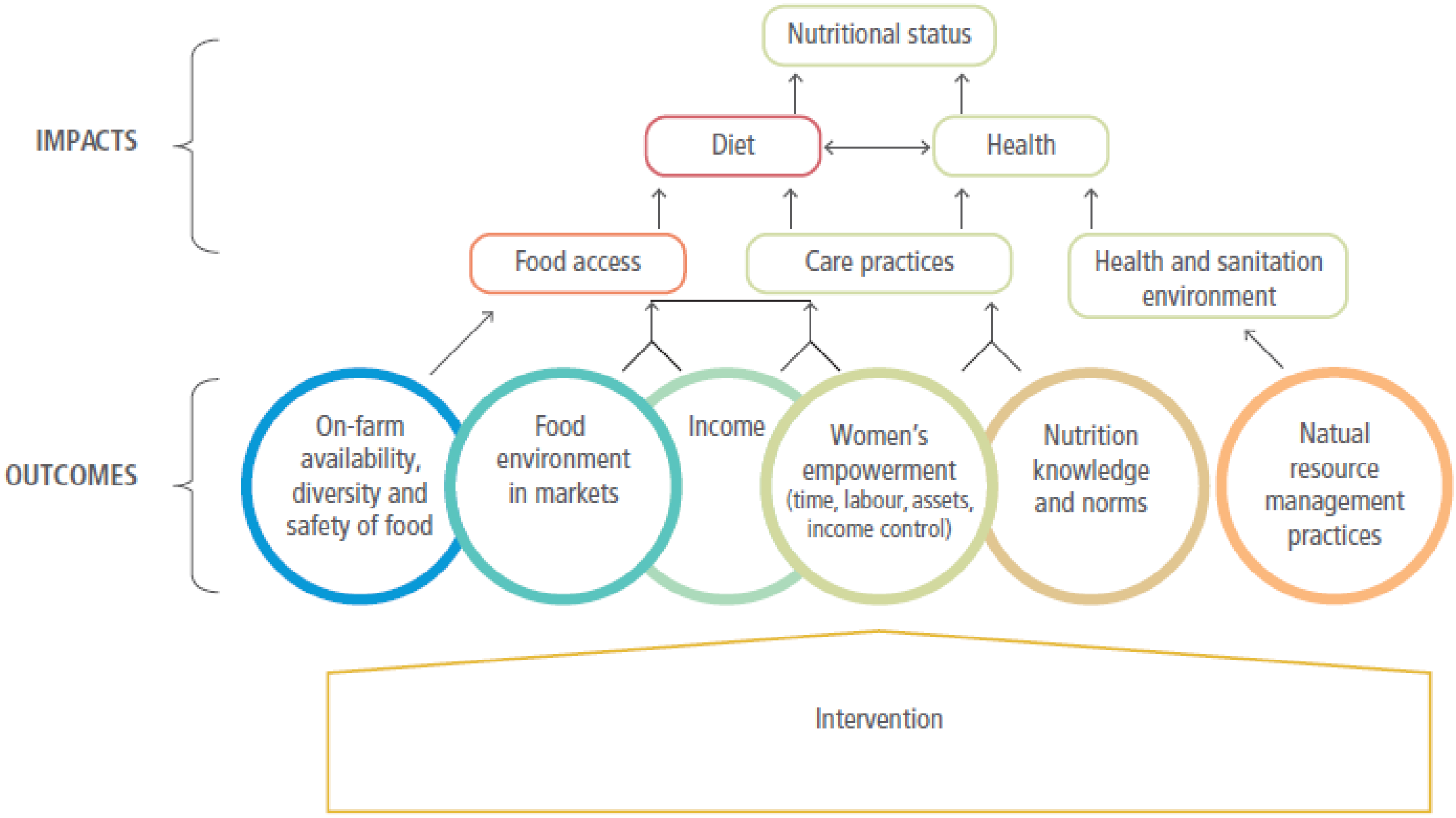

Source: Herforth and Ballard, 2016.[6]

Note: Herforth and Ballard 2016 refers to: Herforth, A. & Ballard, T. 2016. Nutrition indicators in agriculture projects: current measurements, priorities and gaps. Global Food Security. Available at: www.sciencedirect.com/science/article/pii/S22119123415300109

8. Herforth A, Harris J: Improving Nutrition through Agriculture Technical Brief Series: Understanding and Applying Primary Pathways and Principles; 2014

FIGURE. CONCEPTUAL PATHWAYS BETWEEN AGRICULTURE AND NUTRITION

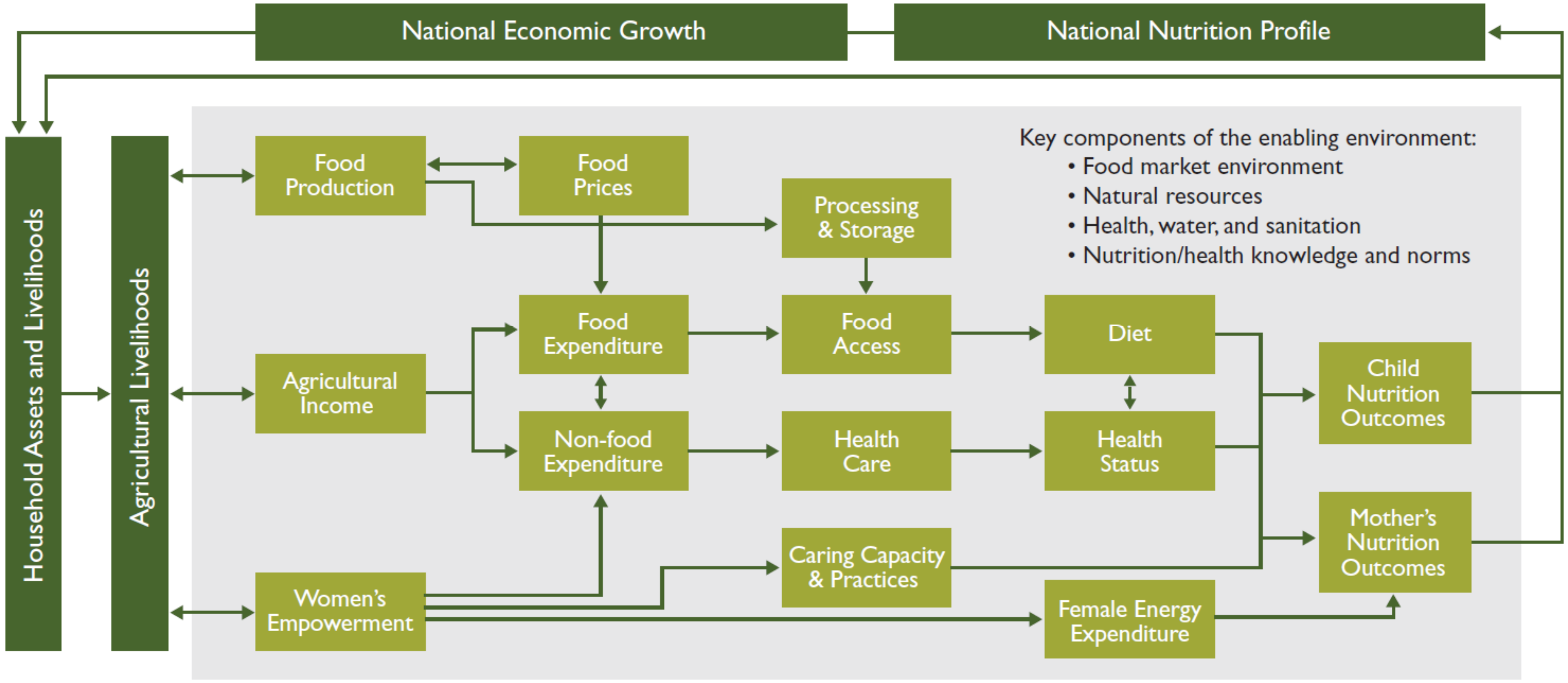

Adapted for Feed the Future by Anna Herforth, Jody Harris, and SPRING, from Gillespie, Harris, and Kadiyala (2012) and Headey, Chiu, and Kadiyala (2011).

9. Kadiyala S, Harris J, Headey D, Yosef S, Gillespie S: **Agriculture and nutrition in India: mapping evidence to pathways.** Annals of the New York Academy of Sciences 2014, 1331(1):43-56. https://nyaspubs.onlinelibrary.wiley.com/doi/full/10.1111/nyas.12477

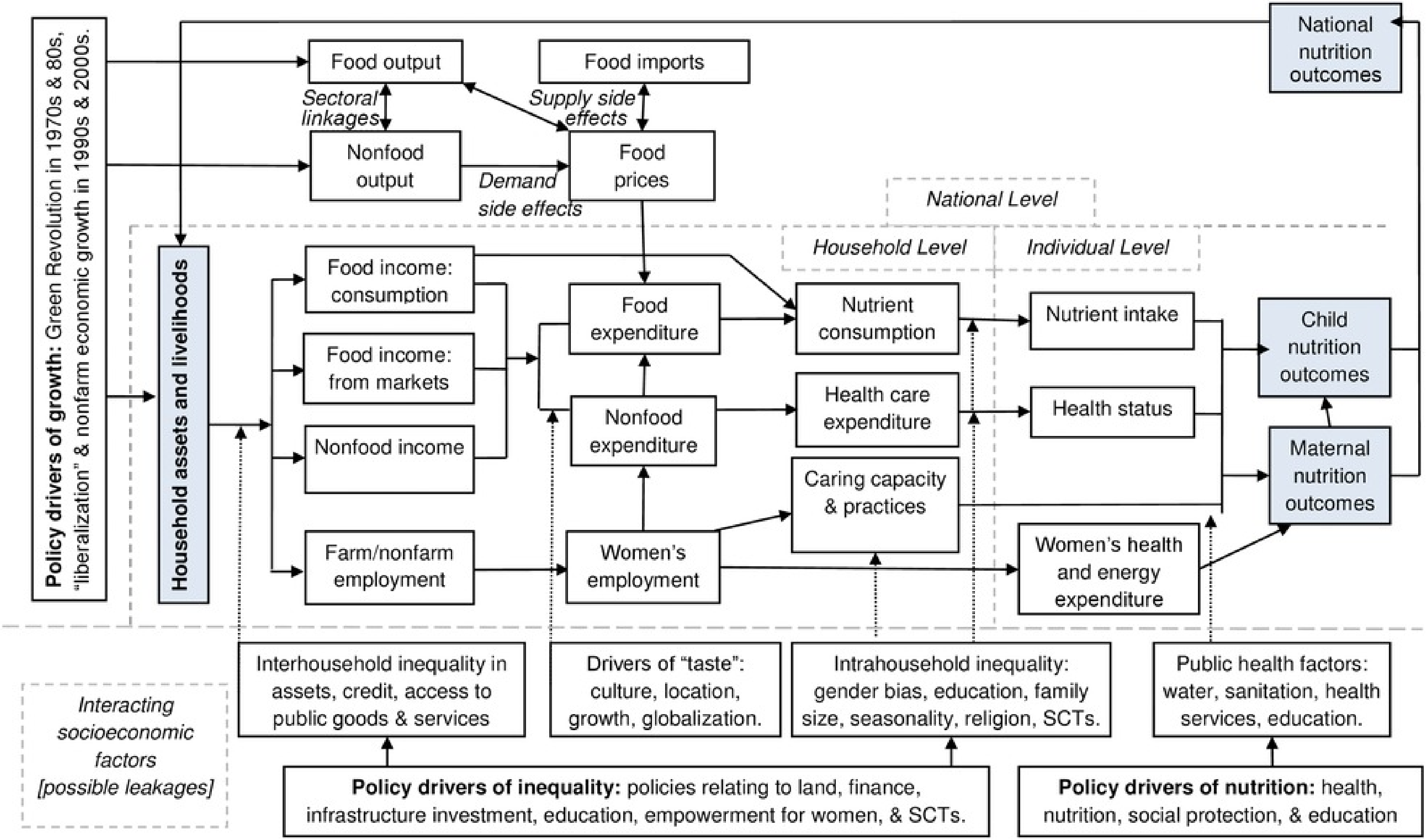

10. Pandey VL, Mahendra Dev S, Jayachandran U: Impact of agricultural interventions on the nutritional status in South Asia: A review. *Food Policy* 2016, 62:28-40. https://doi.org/10.1016/j.foodpol.2016.05.002

Fig. 3. Pathways of agricultural interventions and nutrition.

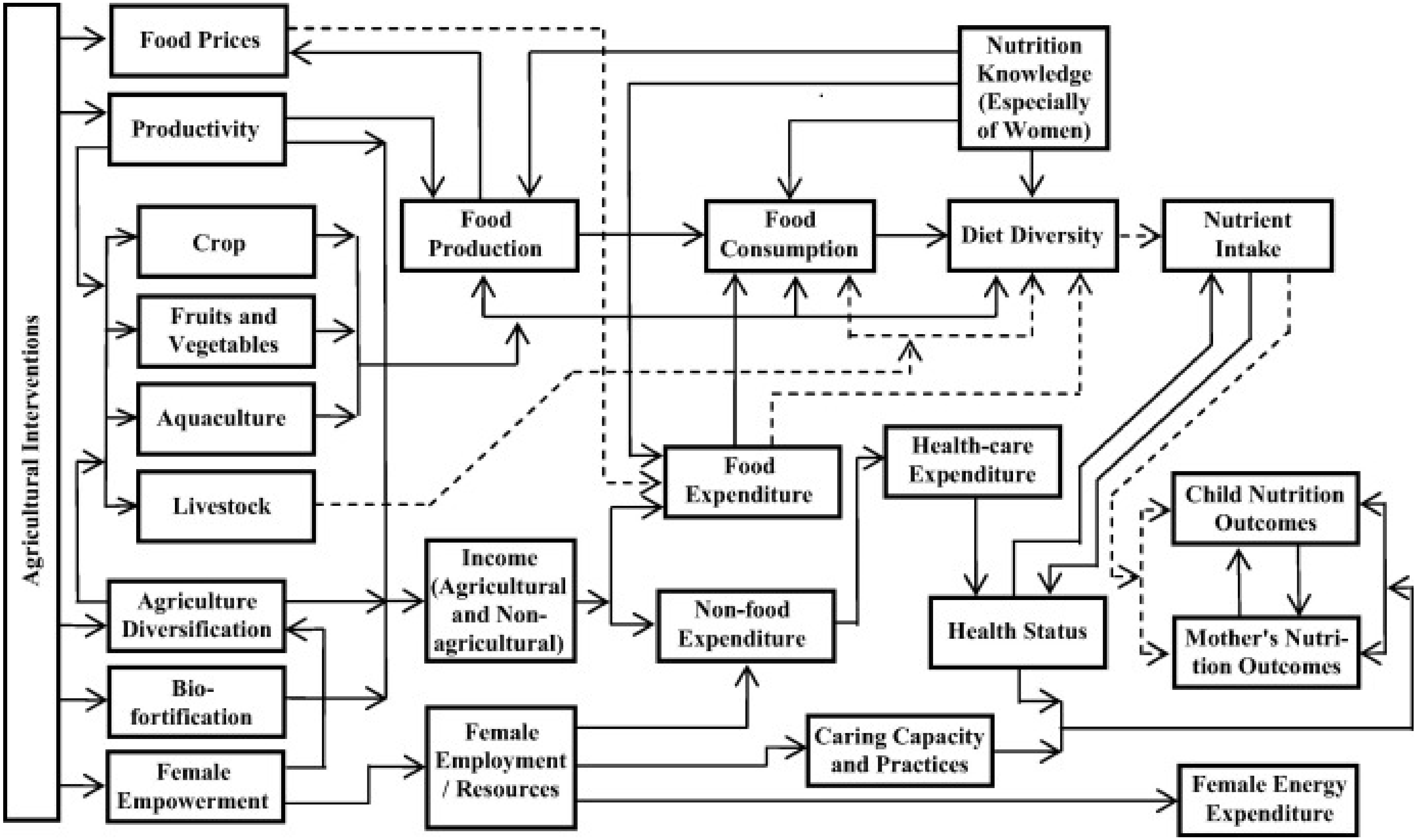

11. Black RE, Singhal A, Uauy R (eds): International Nutrition: Achieving Millennium Goals and Beyond. Nestlé Nutr Inst Workshop Ser. Nestec Ltd. Vevey/S. Karger AG Basel, © 2014, vol 78, pp 93-109. https://doi.org/10.1159/000354946

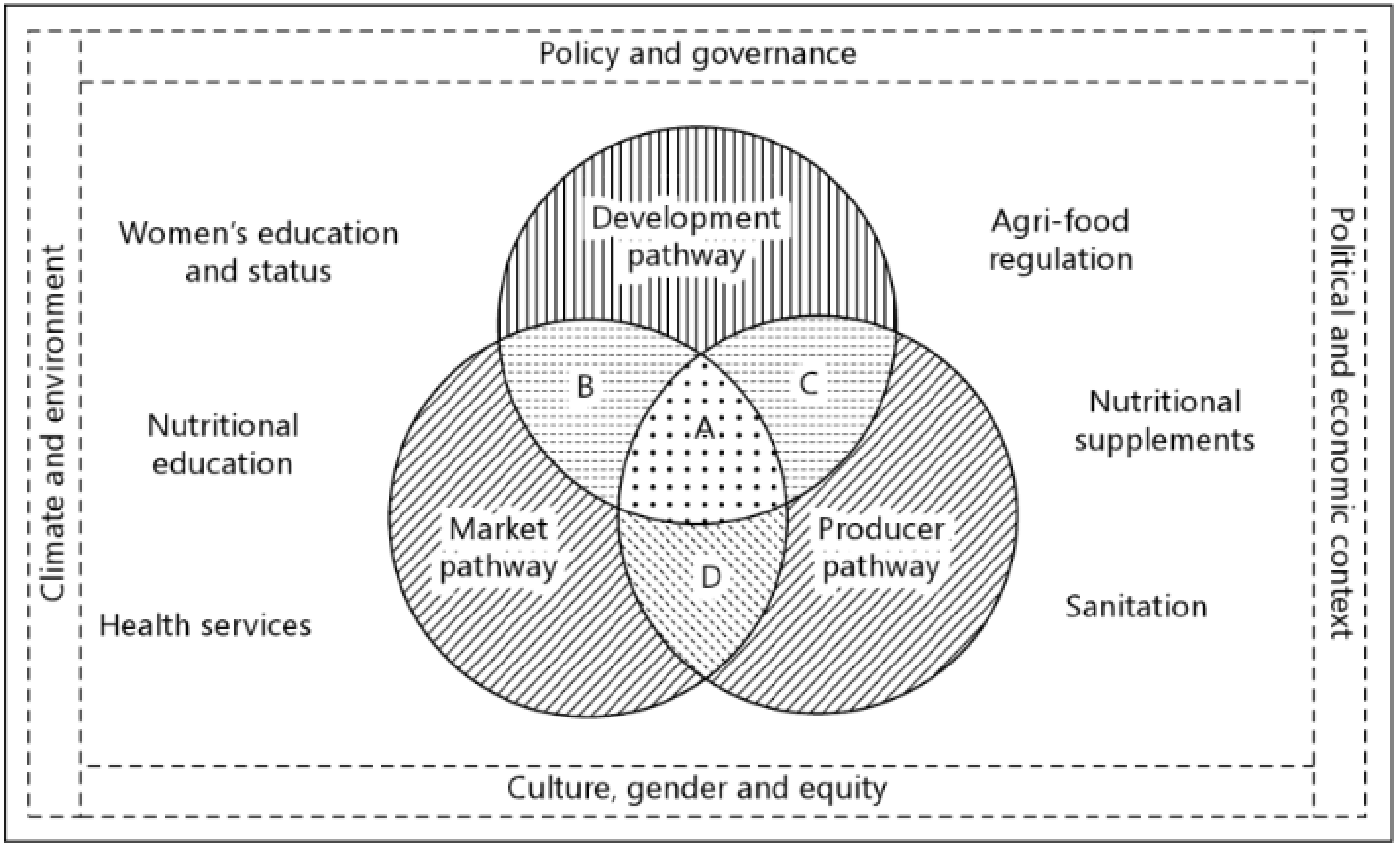

Fig. 2. Overlaps between development, market and own-production pathways for agricultural impacts on nutrition.

12. Webb P: **Impact Pathways from Agricultural Research to Improved Nutrition and Health: Literature Analysis and Research Priorities.** In: ICN2 Second International Conference on Nutrition. Rome, Italy: Food and Agriculture Organization of the United Nations (FAO) and the World Health Organization (WHO) 2013. http://www.fao.org/3/a-as573e.pdf

Figure 1: Logical framework for assessing impact of agricultural interventions on nutrition (as proposed by Masset et al. 2011) with 7 key pathways added.

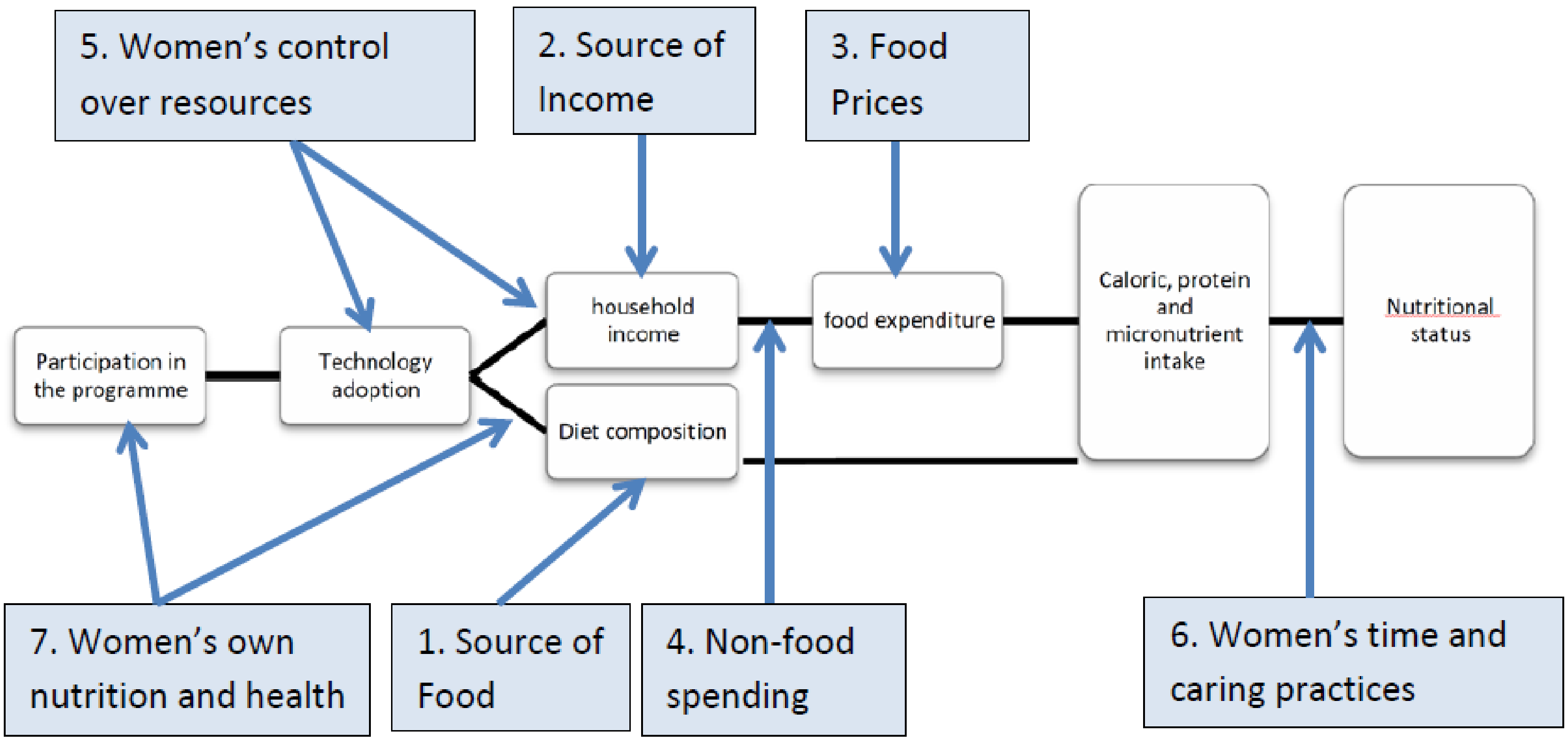

Continued: Webb P: **Impact Pathways from Agricultural Research to Improved Nutrition and Health: Literature Analysis and Research Priorities.** In: ICN2 Second International Conference on Nutrition. Rome, Italy: Food and Agriculture Organization of the United Nations (FAO) and the World Health Organization (WHO) 2013. http://www.fao.org/3/a-as573e.pdf

Figure 2: Standards of evidence for links in the chain from biofortification to nutrition.

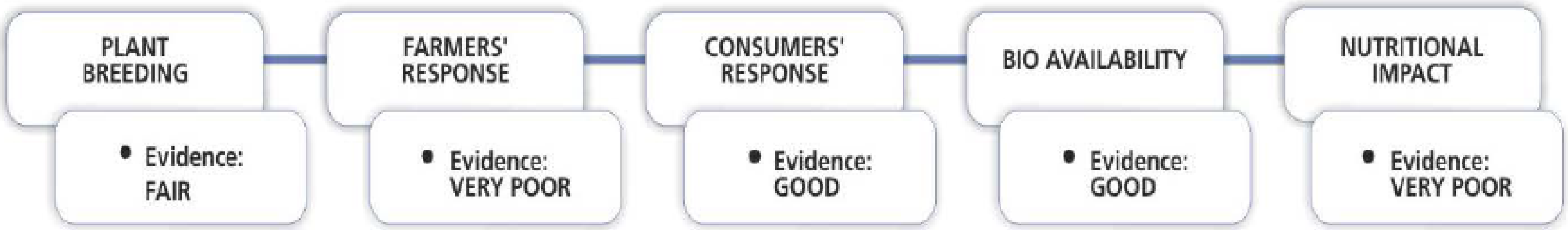

Source: Evidence Matters 2012.

Continued: Webb P: **Impact Pathways from Agricultural Research to Improved Nutrition and Health: Literature Analysis and Research Priorities.** In: ICN2 Second International Conference on Nutrition. Rome, Italy: Food and Agriculture Organization of the United Nations (FAO) and the World Health Organization (WHO) 2013. http://www.fao.org/3/a-as573e.pdf

Figure 3: Adaptation of Logical Framework proposed by Masset et al. (2011)

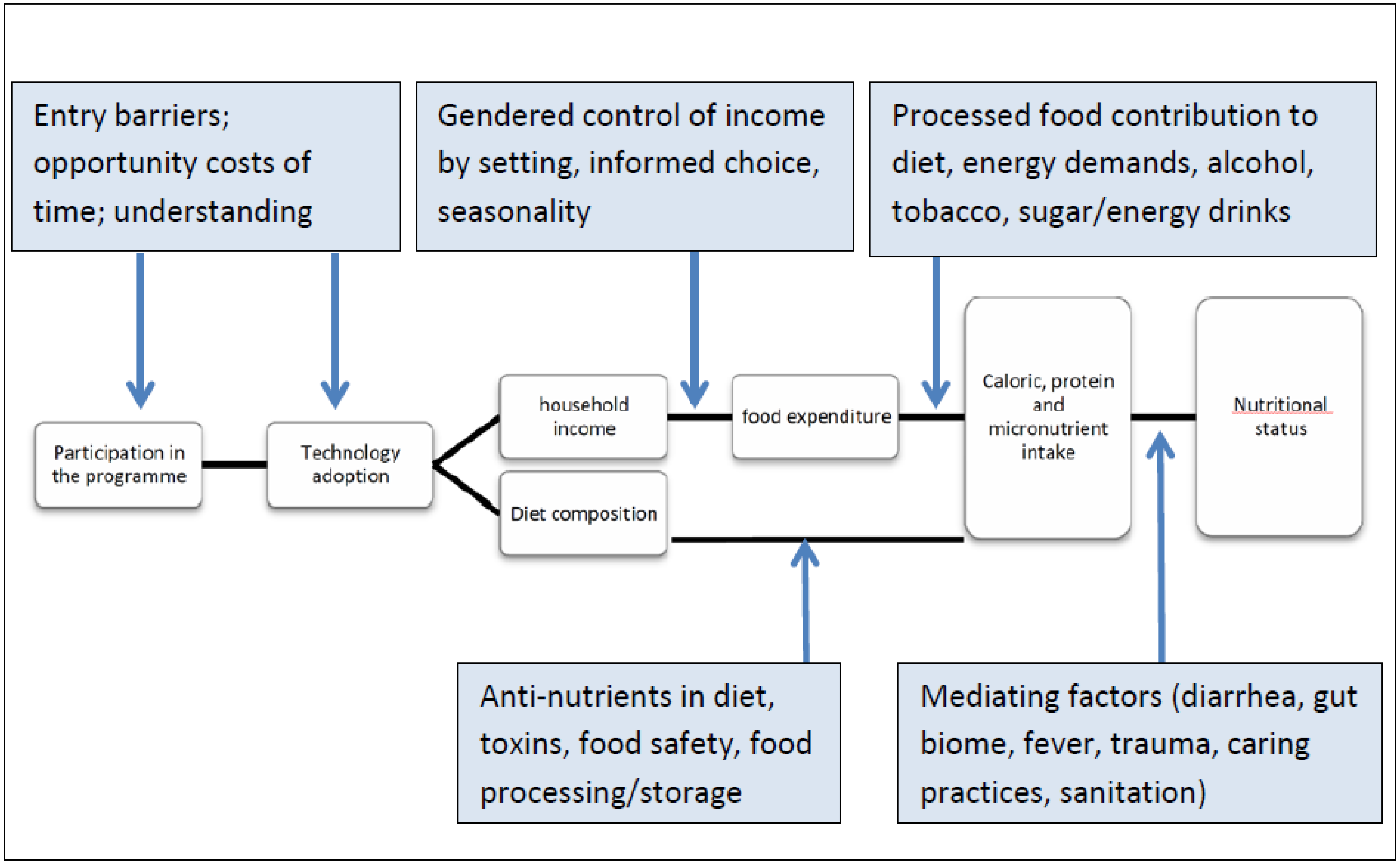

Note: Masset et al. refers to Masset E, Haddad L, Cornelius A, Isaza-Castro J. 2011. A systematic review of agricultural interventions that aim to improve nutritional status of children. London: EPPI-Centre, Social Science Research Unit, Institute of Education. University of London.

Education

1. Efevbera Y, Bhabha J, Farmer PE, Fink G: **Girl child marriage as a risk factor for early childhood development and stunting.** *Soc Sci Med* 2017, 185:91-101.doi:10.1016/j.socscimed.2017.05.027

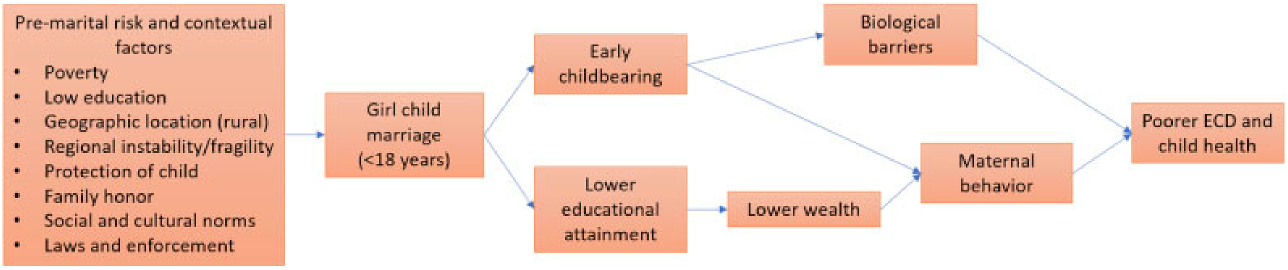

2. Masset E, Gelli A: **Improving Community Development by Linking Agriculture, Nutrition and Education: Design of a Randomised Trial of "Home-Grown" School Feeding in Mali.** *Trials* 2013, 14:55. doi:10.1186/1745-6215-1455

Figure 1: Programme theory of school feeding and impact on education

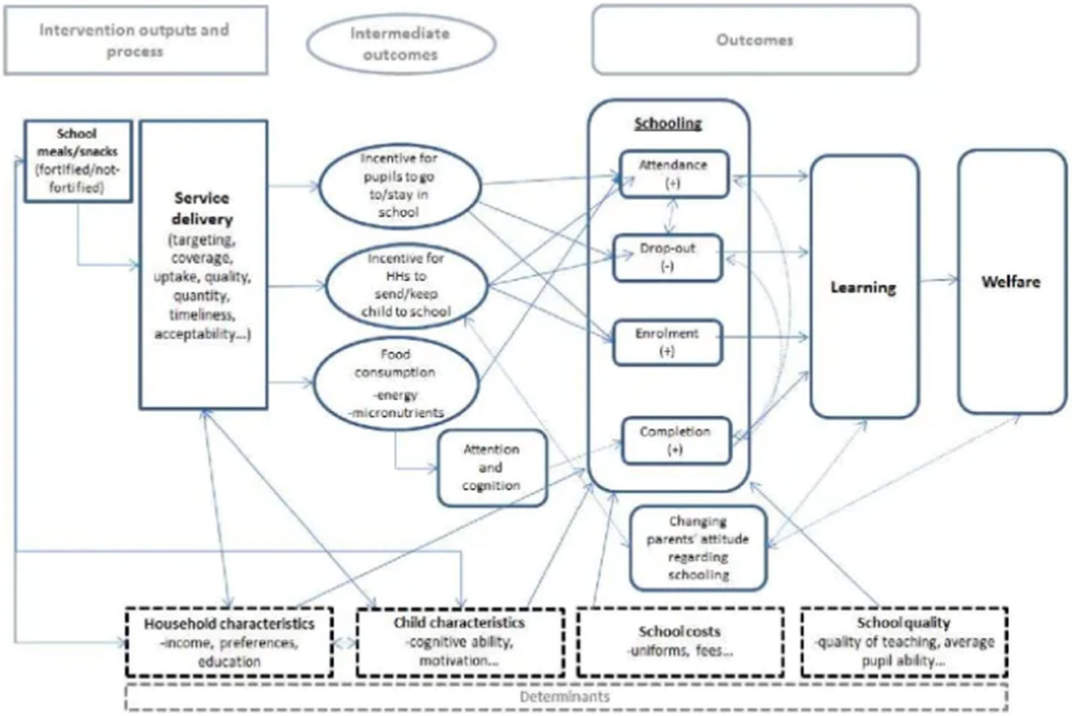

3. Rangel CN, Nunn R, Dysarz F, Silva E, Fonseca AB: Teaching and learning about food and nutrition through science education in Brazilian schools: an intersection of knowledge. In: *Ciência & Saúde Coletiva.* 2014, 19:3915-3924. Available from: http://www.scielo.br/scielo.php?script=sci_arttext&pid=S1413-81232014000903915&lng=en. http://dx.doi.org/10.1590/1413-81232014199.12552013.

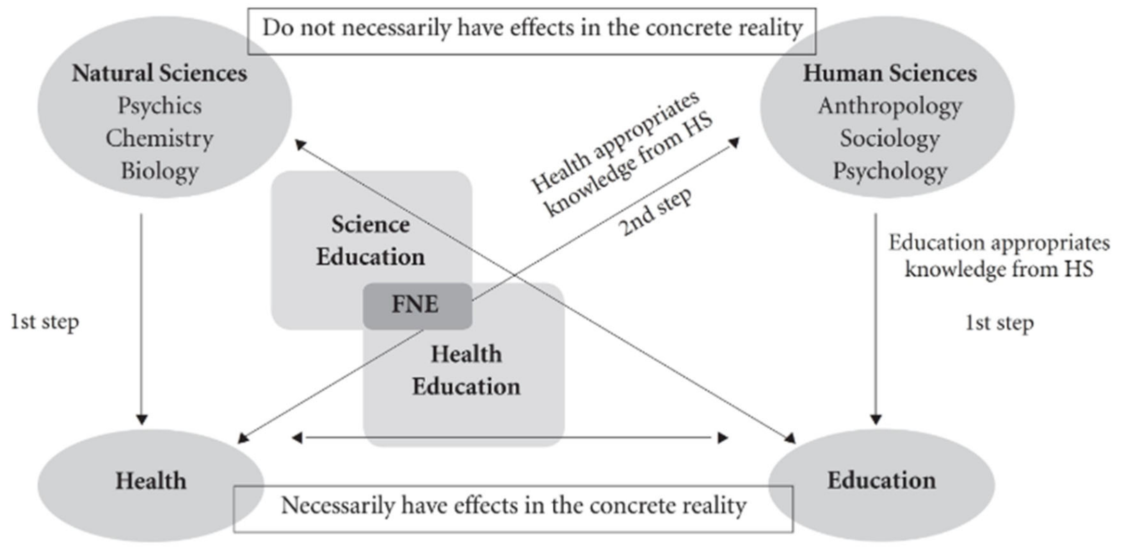

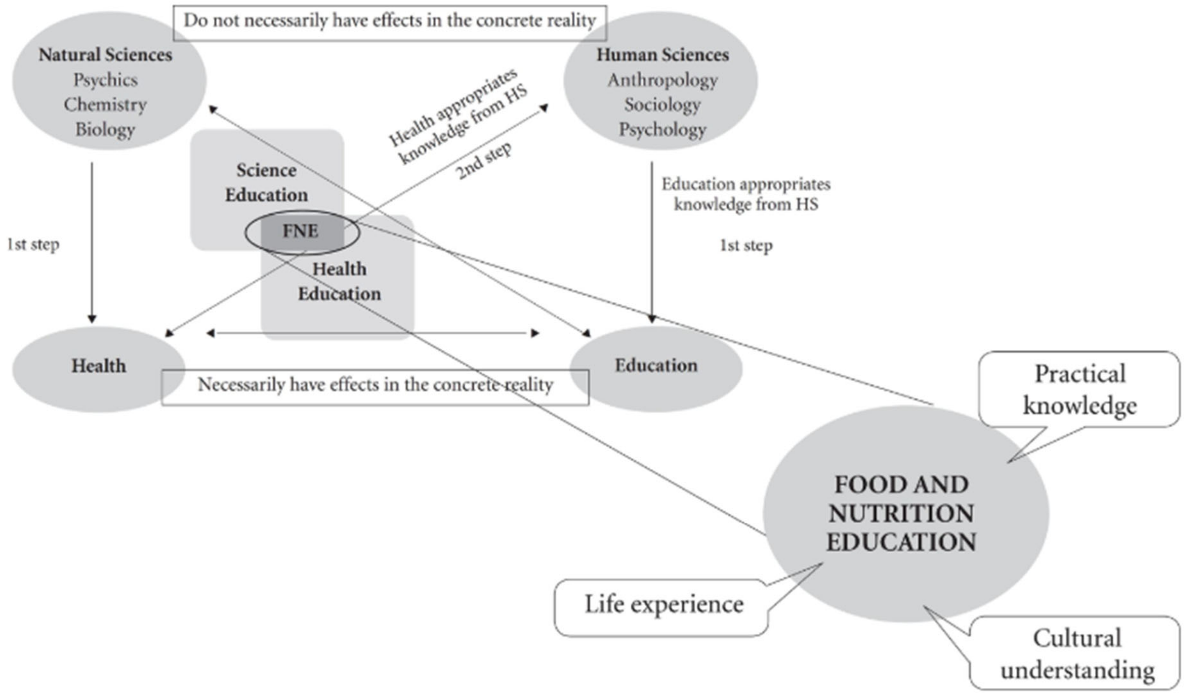

Figure 2 Non-scientific knowledge involved with food and nutrition education

4. Masset E, Gelli A: Improving Community Development by Linking Agriculture, Nutrition and Education: Design of a Randomised Trial of "Home-Grown" School Feeding in Mali. *Trials* 2013, 14:55. doi:10.1186/1745-6215-1455

Figure 11

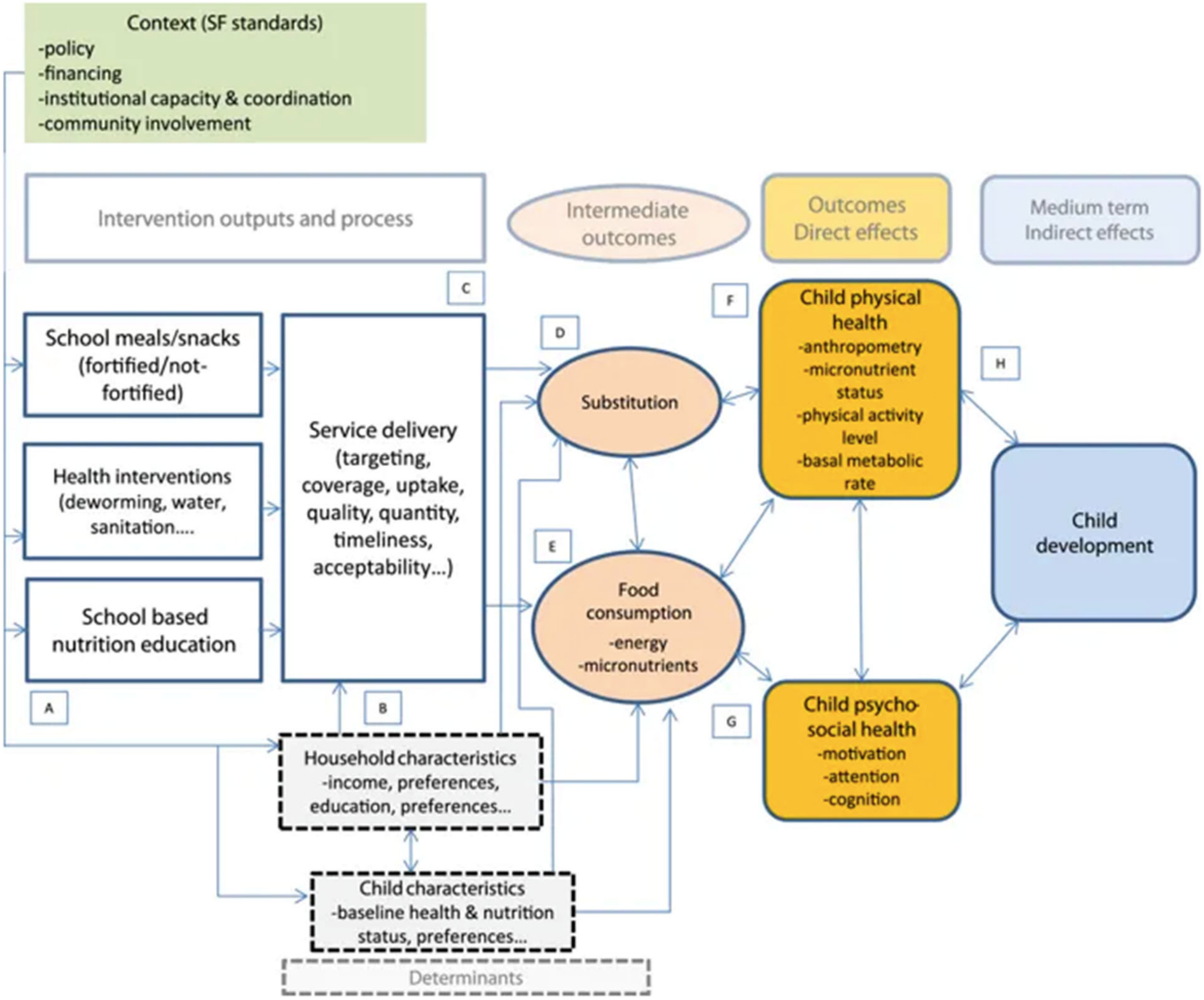

Programme theory of impact on nutrition. The nutritional impact is mediated by the extent of food substitution effects within the household, and the use of the energy intake by the child and her siblings. The reduction in malnutrition via diet diversification and the absorption of micronutrients in the body can have direct effects on cognition. Better nourished children may learn better while in class and outside class.

5. Levinger B. School Feeding, School Reform, and Food Security: Connecting the Dots. *Food and Nutrition Bulletin* 2005, 26(2_suppl2):S170-S178. doi:10.1177/15648265050262S207.

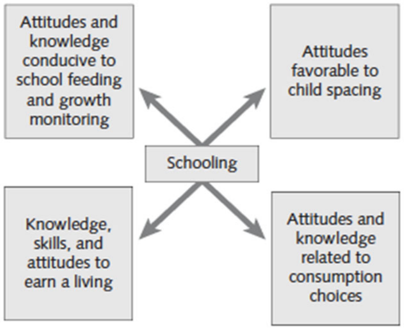

FIG. 3. How access to schooling influences the availability component of food security

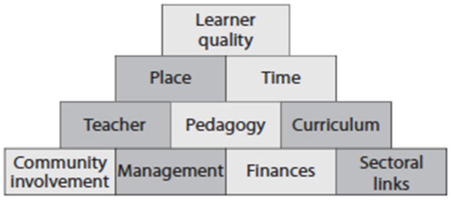

FIG. 6. Education quality building blocks

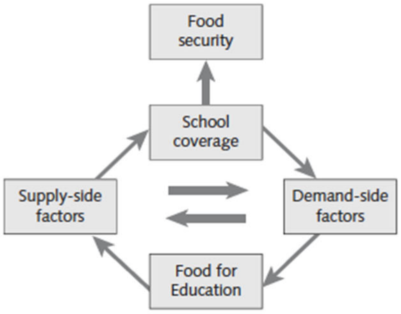

FIG. 5. Food for Education as a determinant of school participation and food security

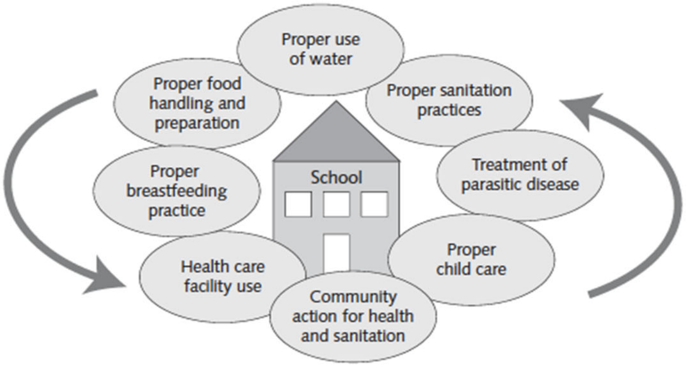

FIG. 4. How access to schooling influences the utilization component of food security

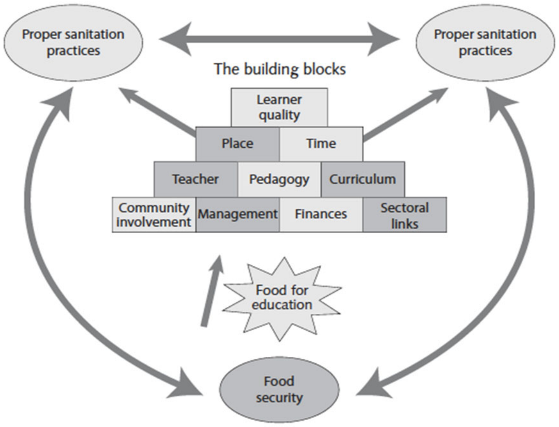

FIG. 8. Achieving the full potential of Food for Education

6. McCoy DC, Zuilkowski SS, Fink G: **Poverty, physical stature, and cognitive skills: Mechanisms underlying children's school enrollment in Zambia.** *Developmental Psychology* 2015, 51(5):600-614. doi:10.1037/a0038924

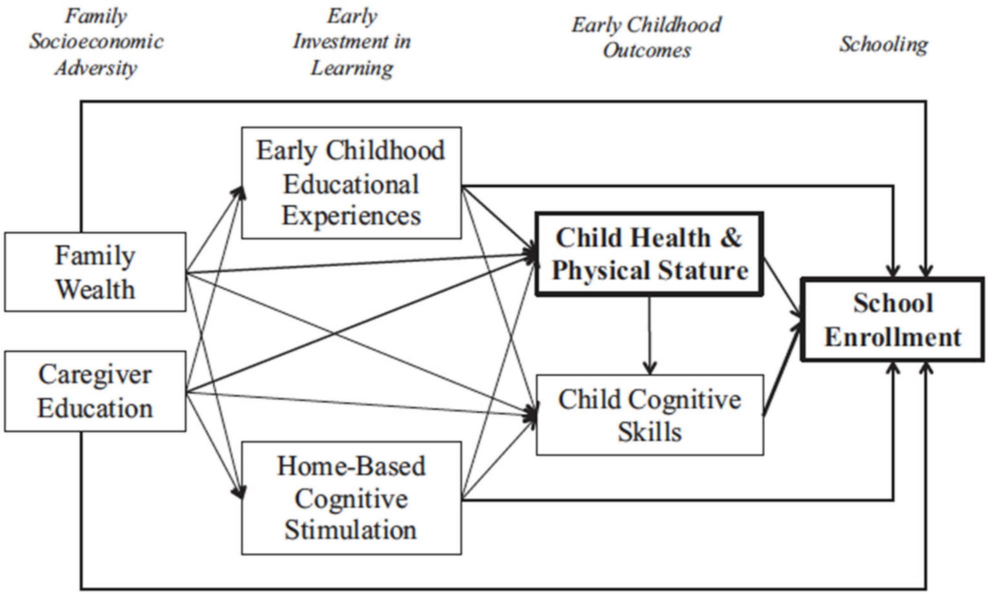

Figure 1. Proposed theoretical model of the relations between family SES, early investment in learning, child development, and schooling in low- and middle-income countries. Bold variables and paths indicate unique contributions over previous models of early development from the United States.

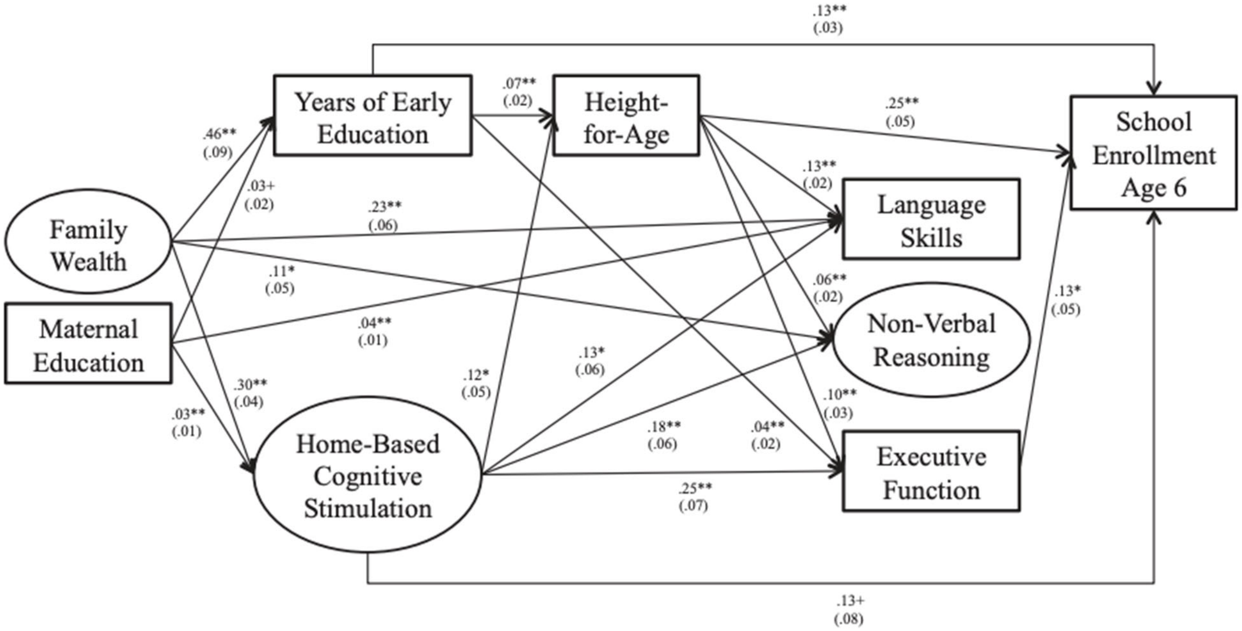

Figure 3. Final early (age 6) enrollment structural equation model results (full sample; $n = 2{,}711$). Notes: $^{+} p < .10$. $^{*} p < .05$. $^{**} p < .01$. Only significant, direct pathways shown. See Table 5 for coefficients and standard errors of significant indirect pathways. Covariates (child gender, child age, household size, region urbanicity, region wealth), residual correlations (between cognitive stimulation variables, and between cognitive skill variables), and embedded measurement models (for family wealth, home-based cognitive stimulation, and nonverbal reasoning) included in model but not shown.

7. Torres I, Benn J: The rural school meal as a site for learning about food. *Appetite*. 2017, 117:29-39. doi:10.1016/j.appet.2017.05.055

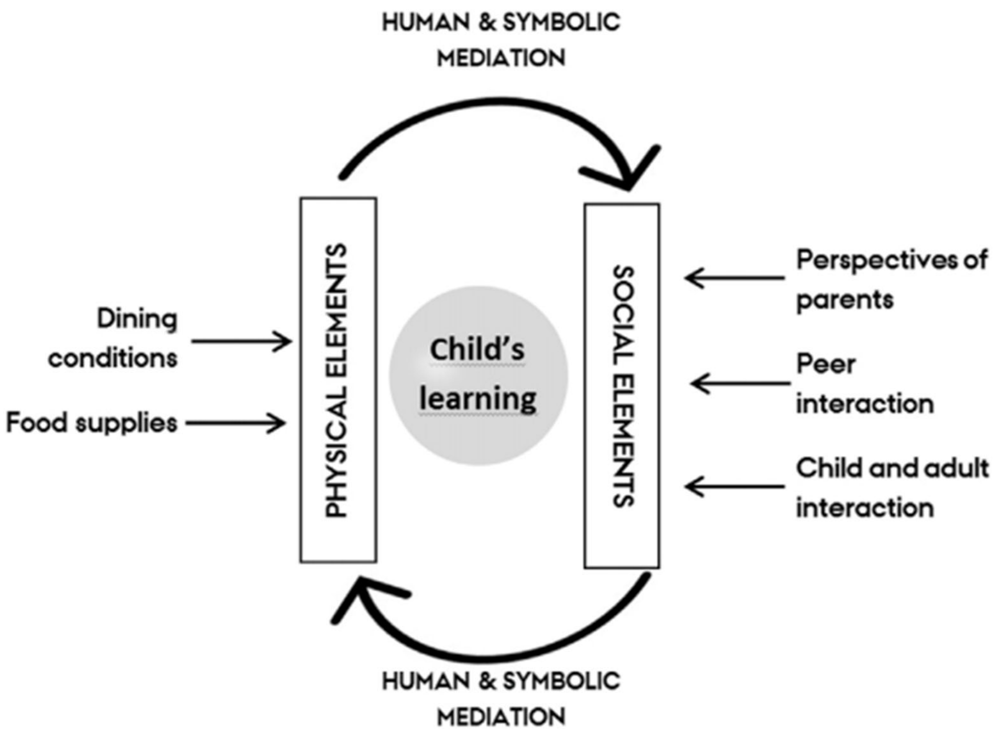

Fig. 1. The school meal as a site for learning.

8. Studdert LJ, Soekirman, Rasmussen KM, Habicht J-P: Community-Based School Feeding during Indonesia'S Economic Crisis: Implementation, Benefits, and Sustainability. *Food Nutrition Bulletin*. 2004, 25(2):156-165. doi:10.1177/156482650402500208

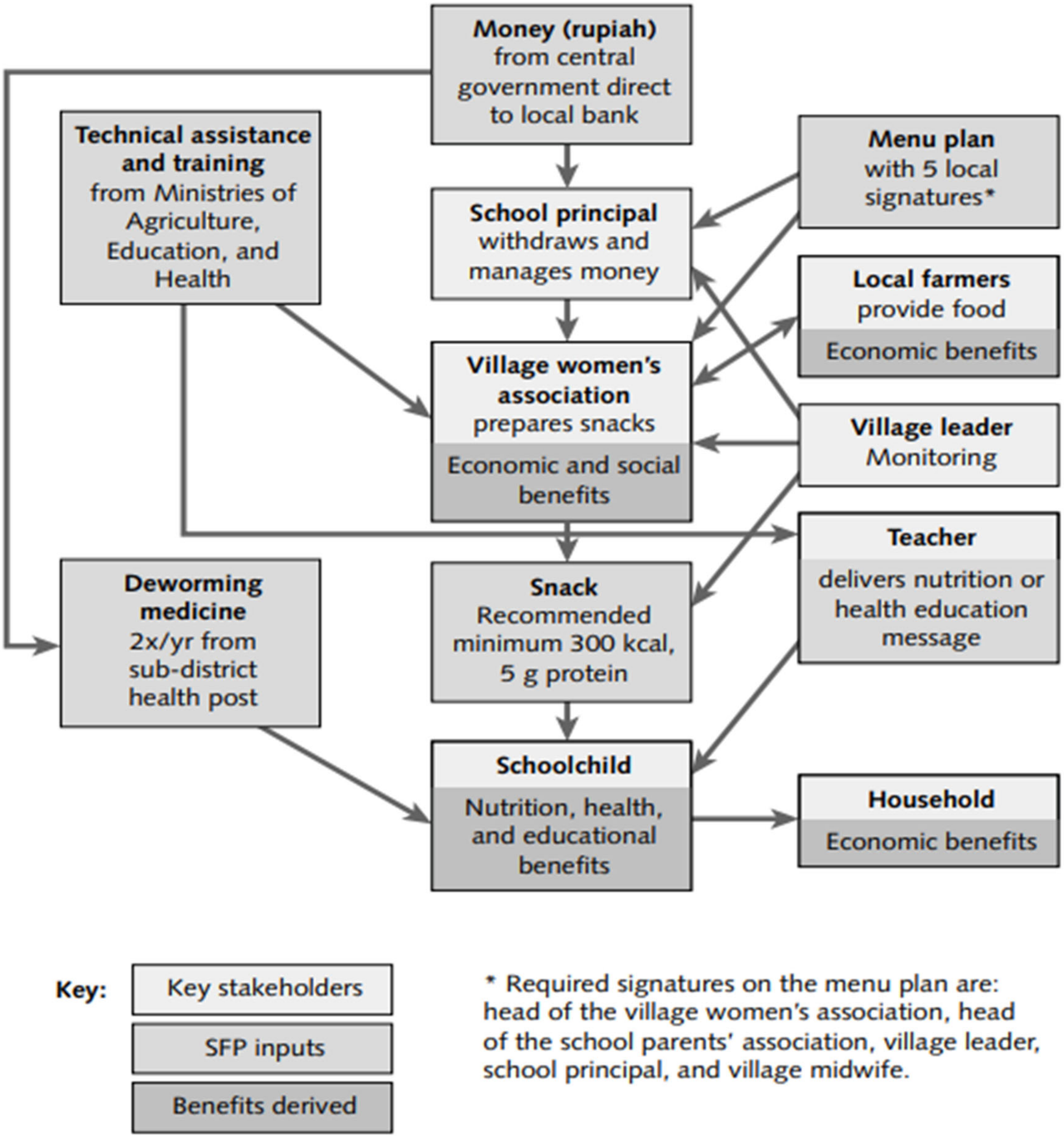

FIG. 1. Implementation flowchart for the Indonesia school-feeding program

9. Schwartzman F, Mora CAR, Bogus CM, Villar BS: **Background and elements of the linkage between the Brazilian school feeding program and family farming.** *Cad Saude Publica*. 2017, 33(12):e00099816. doi:10.1590/0102-311X00099816

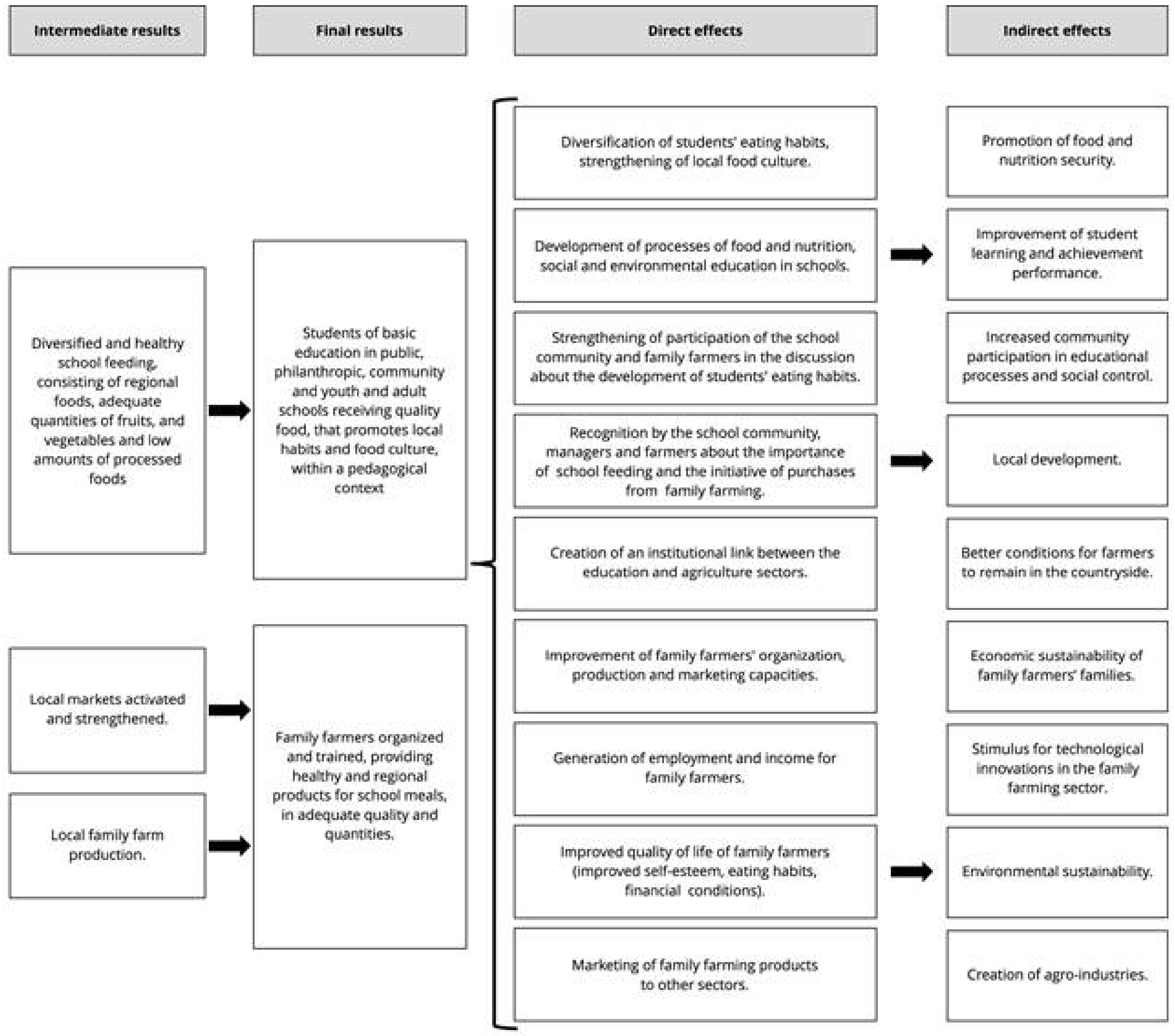

Continued: Schwartzman F, Mora CAR, Bogus CM, Villar BS: **Background and elements of the linkage between the Brazilian school feeding program and family farming.** *Cad Saude Publica*. 2017, 33(12):e00099816. doi:10.1590/0102-311X00099816

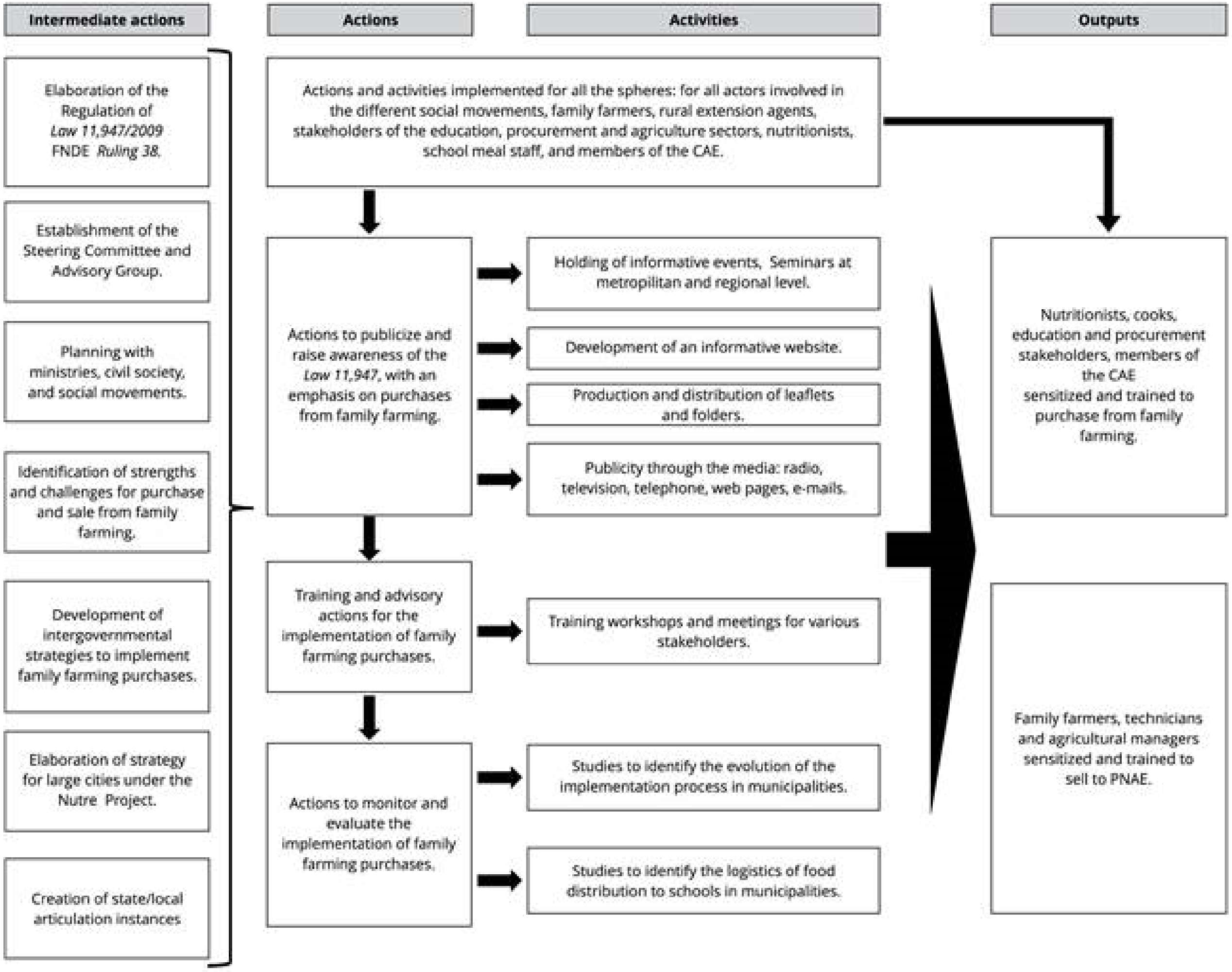

CAE: School Feeding Council; FNDE: Brazilian National Fund for Education Development.

WASH

1. Cumming O, Cairncross S: **Can water, sanitation and hygiene help eliminate stunting? Current evidence and policy implications.** *Matern Child Nutr* 2016, 12(S1):91-105. https://doi.org/10.1111/mcn.12258

Note: Figure 1. Reproduction of the F-diagram, adapted from the original Wagner & Lanoix (1958)

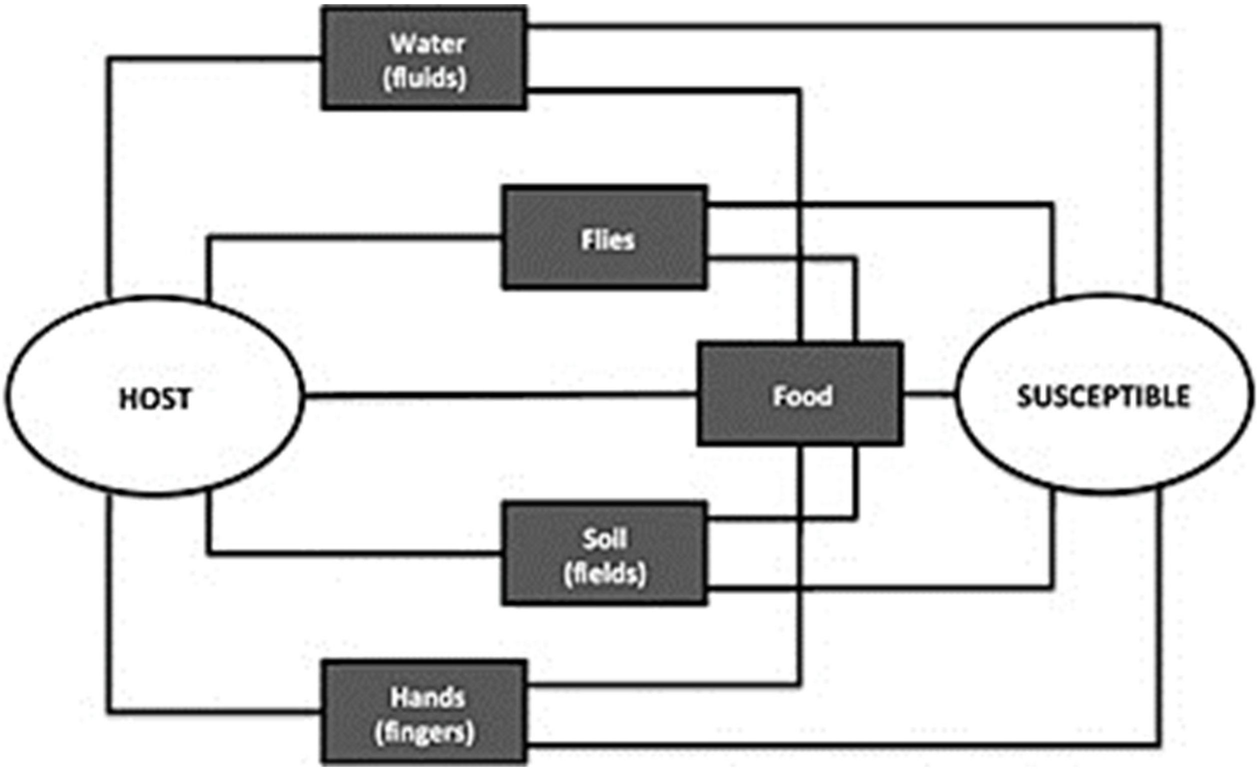

2. Wood S, Foster J, Kols A: **Understanding why women adopt and sustain home water treatment: Insights from the Malawi antenatal care program.** *Social Science & Medicine* 2012, 75(4):634-642. https://doi.org/10.1016/j.socscimed.2011.09.018

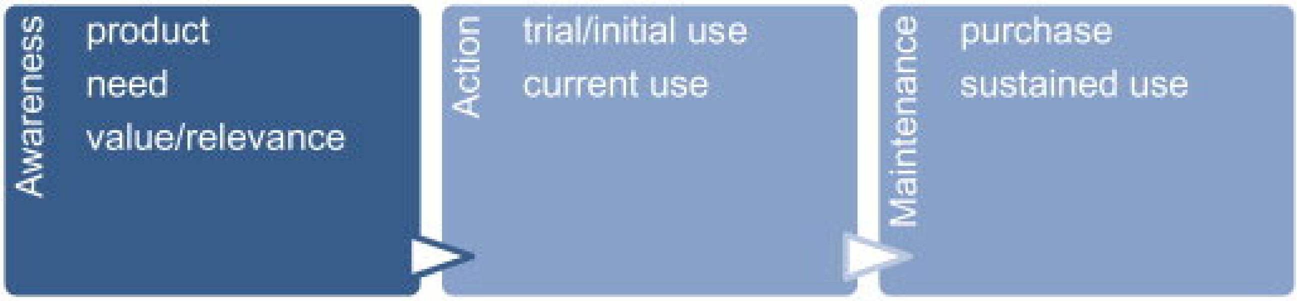

3. Dangour AD, Watson L, Cumming O, Boisson S, Che Y, Velleman Y, Cavill S, Allen E, Uauy R: **Interventions to improve water quality and supply, sanitation and hygiene practices, and their effects on the nutritional status of children.** *Cochrane Database of Systematic Reviews* 2013(8). https://doi.org/10.1002/14651858.CD009382.pub2

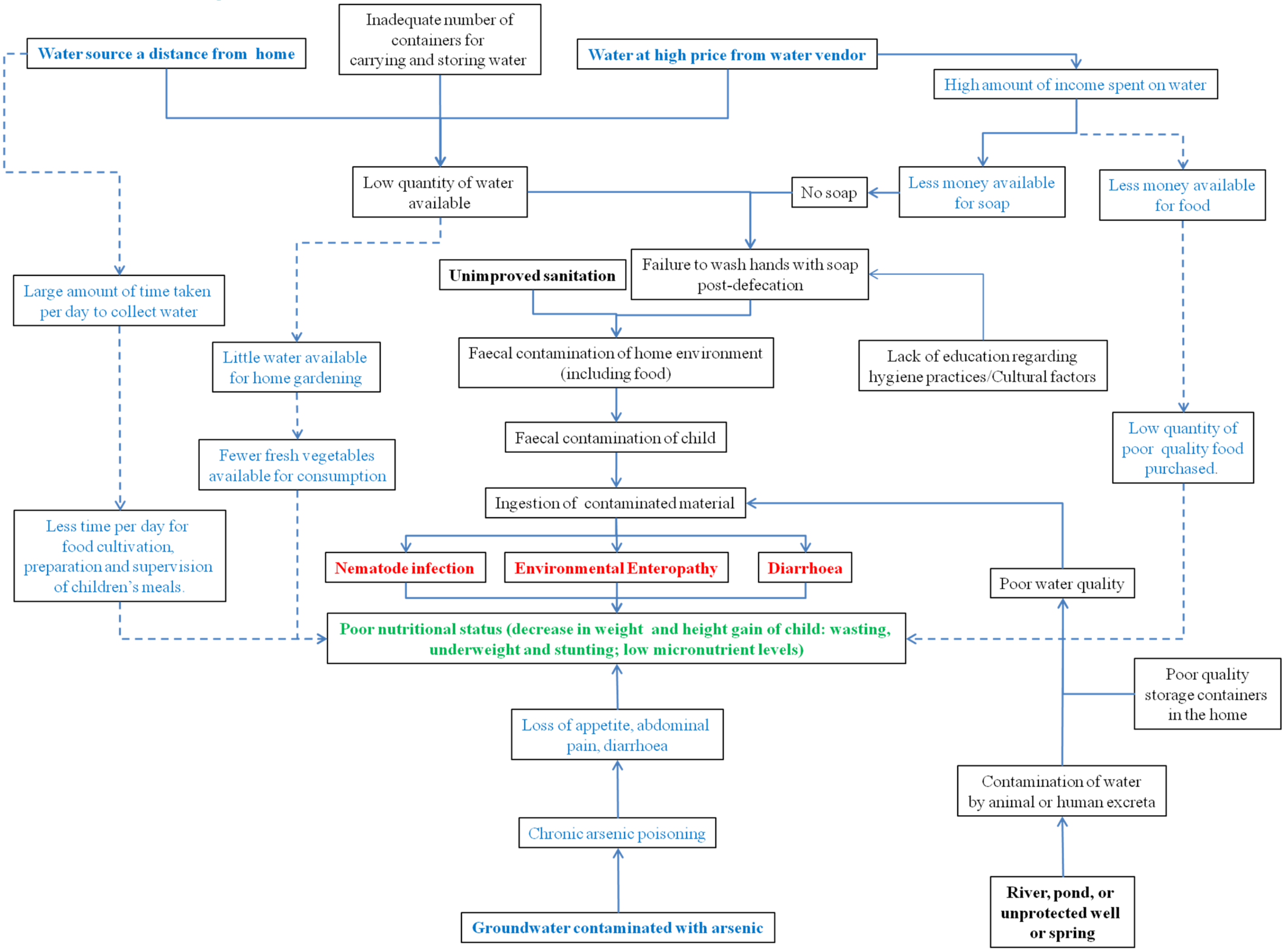

Continued: Dangour AD, Watson L, Cumming O, Boisson S, Che Y, Velleman Y, Cavill S, Allen E, Uauy R: **Interventions to improve water quality and supply, sanitation and hygiene practices, and their effects on the nutritional status of children.** Cochrane Database of Systematic Reviews 2013(8). https://doi.org/10.1002/14651858.CD009382.pub2

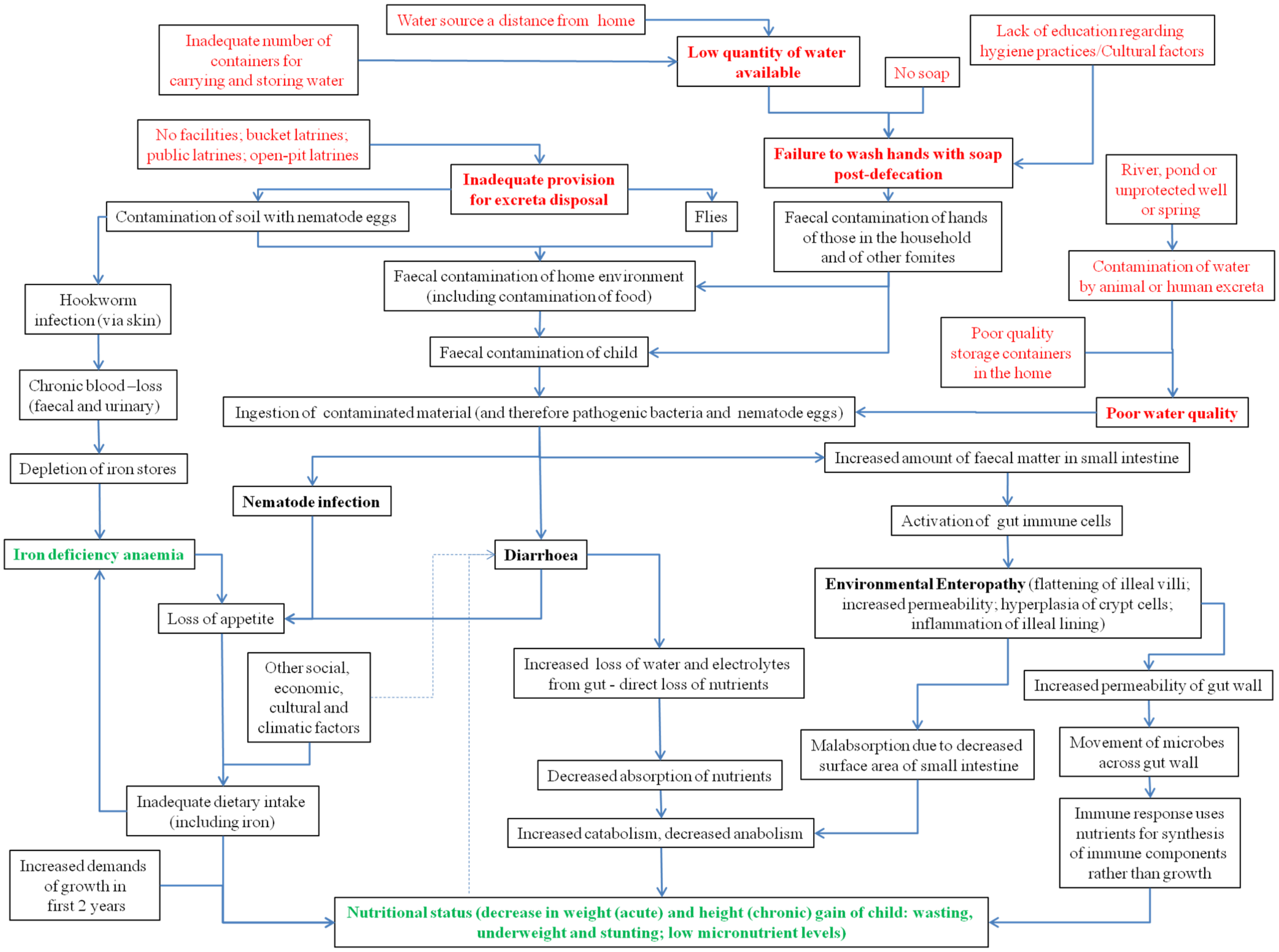

4. Dearden KA, Schott W, Crookston BT, Humphries DL, Penny ME, Behrman JR, Cueto S, Duc LT, Escobal J, Fernald L *et al*: **Children with access to improved sanitation but not improved water are at lower risk of stunting compared to children without access: a cohort study in Ethiopia, India, Peru, and Vietnam.** *BMC Public Health* 2017, 17(1):110. https://doi.org/10.1186/s12889-017-4033-1

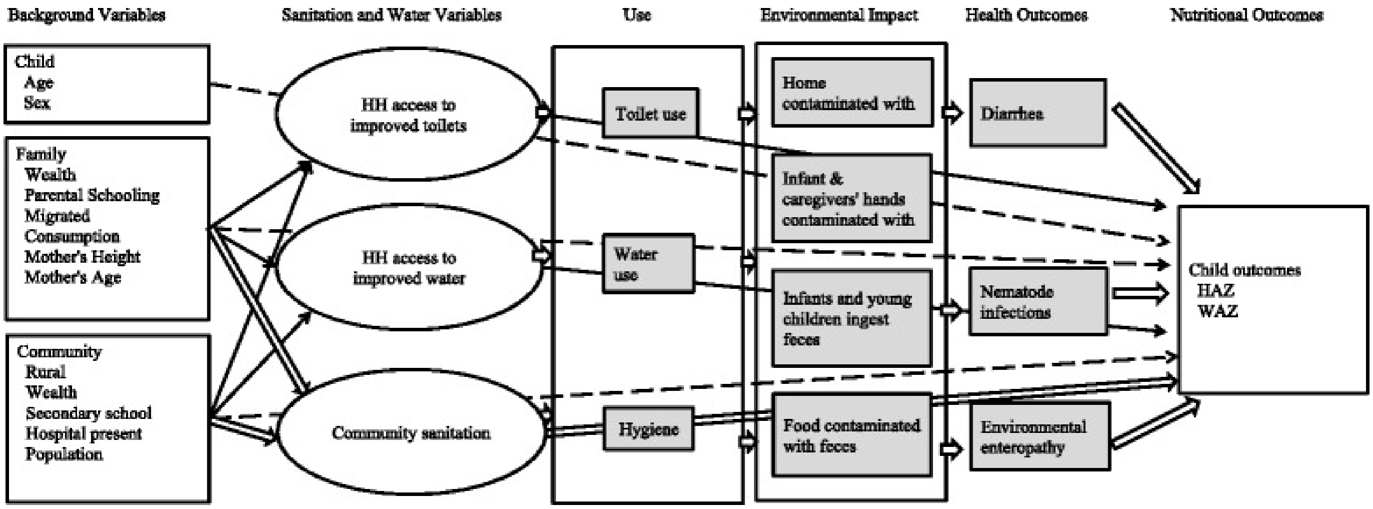

5. Curtis V, Schmidt W, Luby S, Florez R, Touré O, Biran A: **Hygiene: new hopes, new horizons.** *The Lancet Infectious Diseases* 2011, 11(4):312-321. https://doi.org/10.1016/S1473-3099(10)70224-3

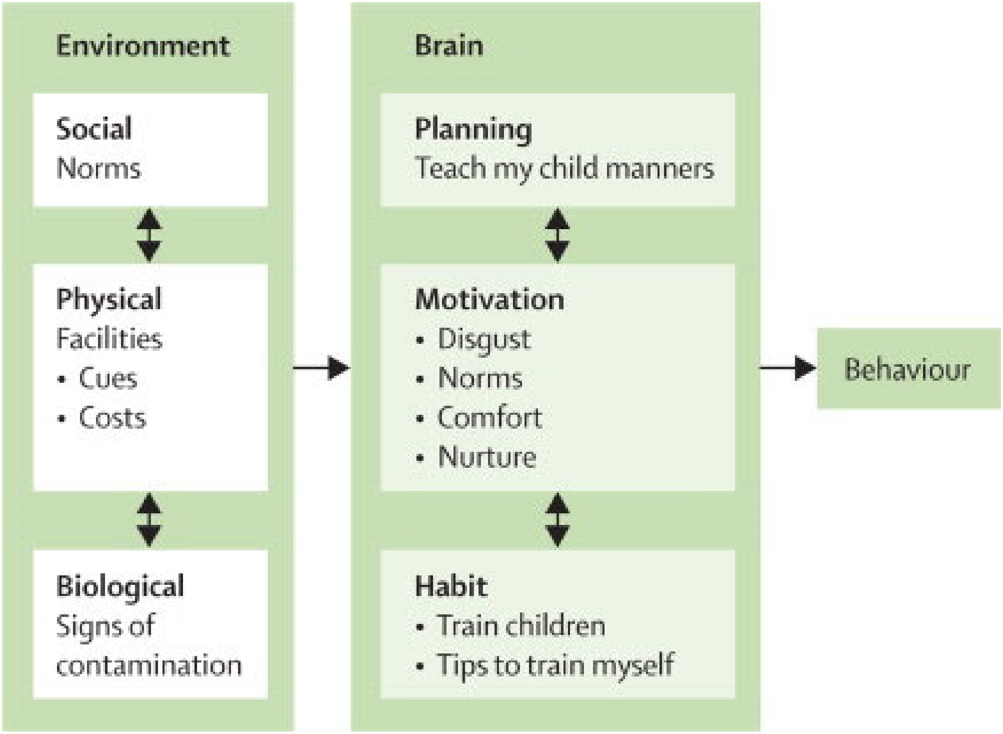

6. Jenkins MW, Scott B: Behavioral indicators of household decision-making and demand for sanitation and potential gains from social marketing in Ghana. *Social Science & Medicine* 2007, 64(12):2427-2442. https://doi.org/10.1016/j.socscimed.2007.03.010

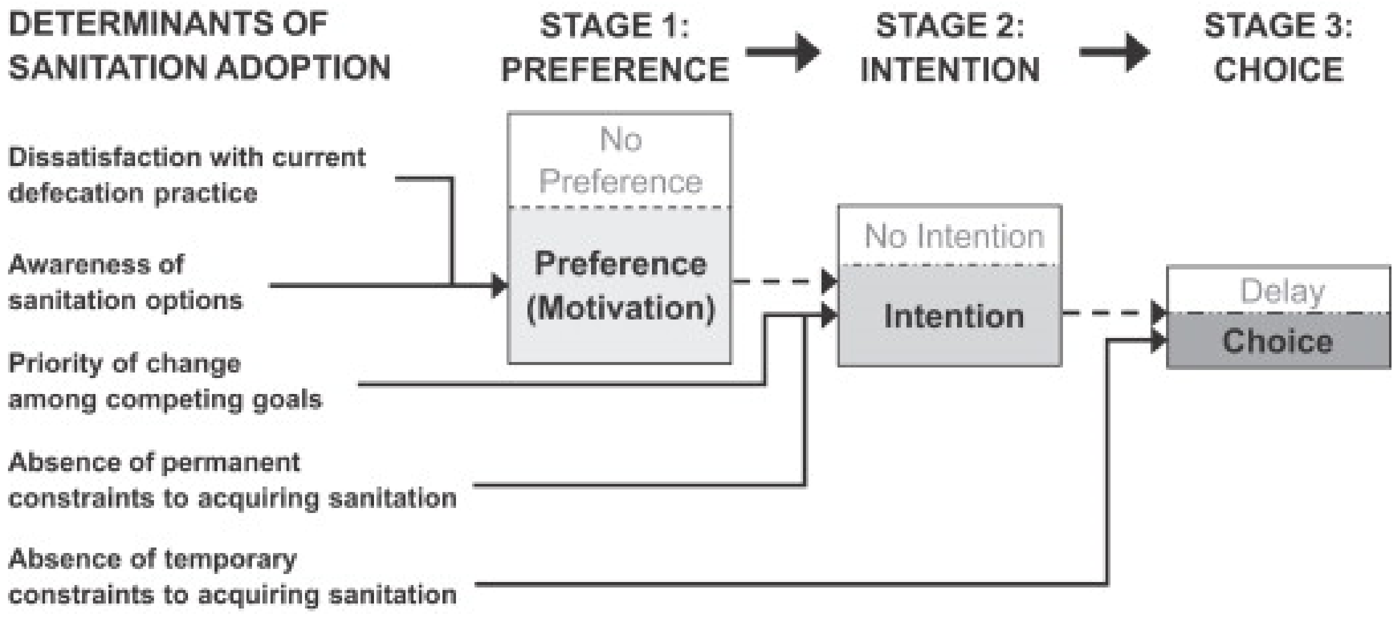

7. WHO and UNICEF Joint Monitoring Programme for Water Supply, Sanitation, and Hygiene: **Service level ladders for monitoring drinking water, sanitation, and hygiene**. 2017.

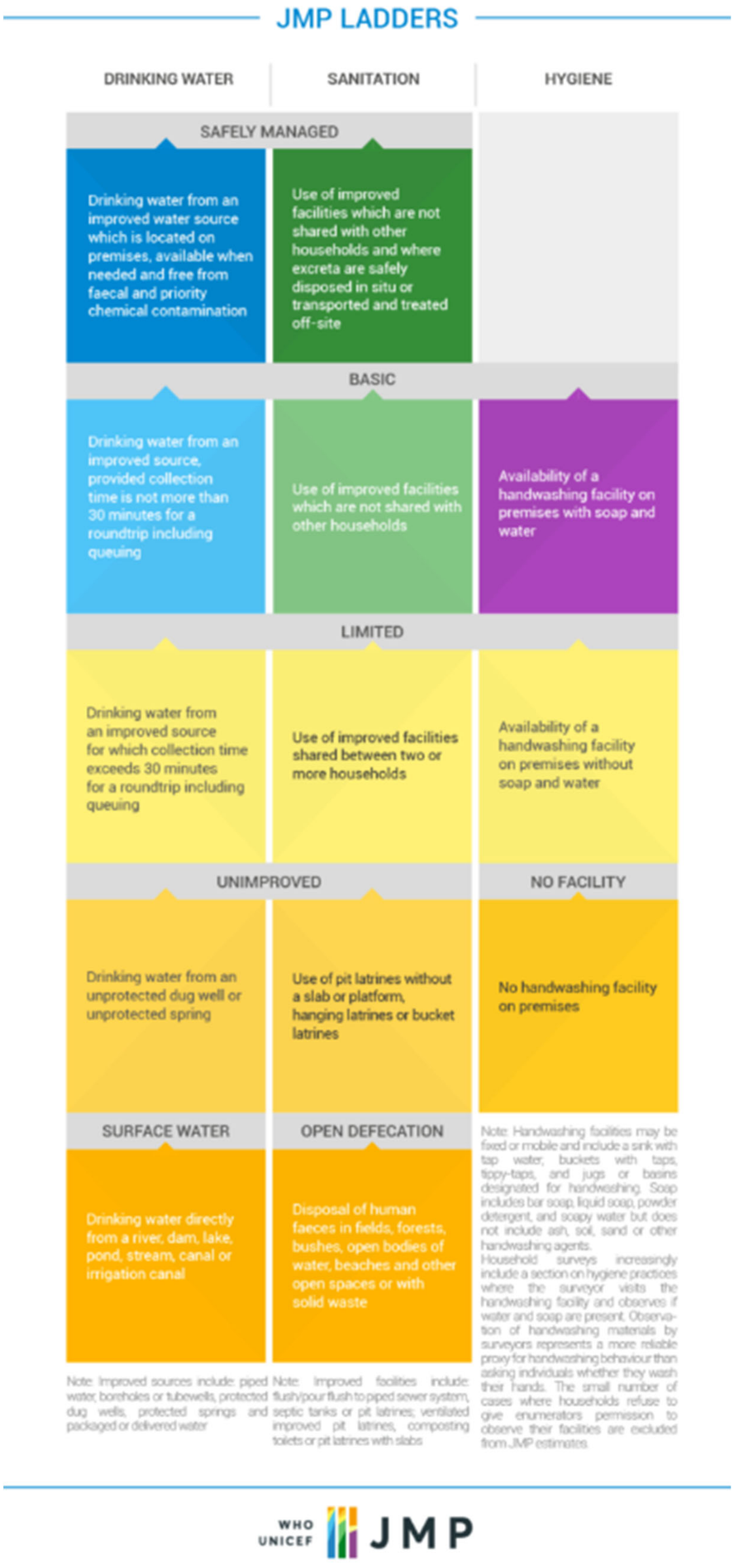

8.

9. WHO and UNICEF Joint Monitoring Programme for Water Supply, Sanitation, and Hygiene: **Classification of improved and unimproved drinking water and sanitation facility types.** 2017.

JMP classification of improved and unimproved facility types

	DRINKING WATER[2]	SANITATION
**Improved facilities**	Piped supplies<br>• Tap water in the dwelling, yard or plot<br>• Public standposts<br>Non-piped supplies<br>• Boreholes/tubewells<br>• Protected wells and springs<br>• Rainwater<br>• Packaged water, including bottled water and sachet water<br>• Delivered water, including tanker trucks and small carts	Networked sanitation<br>• Flush and pour flush toilets connected to sewers<br>On-site sanitation<br>• Flush and pour flush toilets or latrines connected to septic tanks or pits<br>• Ventilated improved pit latrines<br>• Pit latrines with slabs<br>• Composting toilets, including twin pit latrines and container-based systems
**Unimproved facilities**	Non-piped supplies<br>• Unprotected wells and springs	On-site sanitation<br>• Pit latrines without slabs<br>• Hanging latrines<br>• Bucket latrines
**No facilities**	Surface water	Open defecation

[2] The JMP recognizes that bottled water and tanker truck water can potentially deliver safe water, but has previously treated them as unimproved due to lack of data on accessibility, availability and quality. From now on, the JMP will treat them as improved and classify households as having 'limited', 'basic' or 'safely managed' services, based on the accessibility, availability and quality criteria.

10. Mbuya MNN, Humphrey JH: Preventing environmental enteric dysfunction through improved water, sanitation and hygiene: an opportunity for stunting reduction in developing countries. *Matern Child Nutr* 2016, 12 Suppl 1(Suppl Suppl 1):106-120. https://doi.org/10.1111/mcn.12220

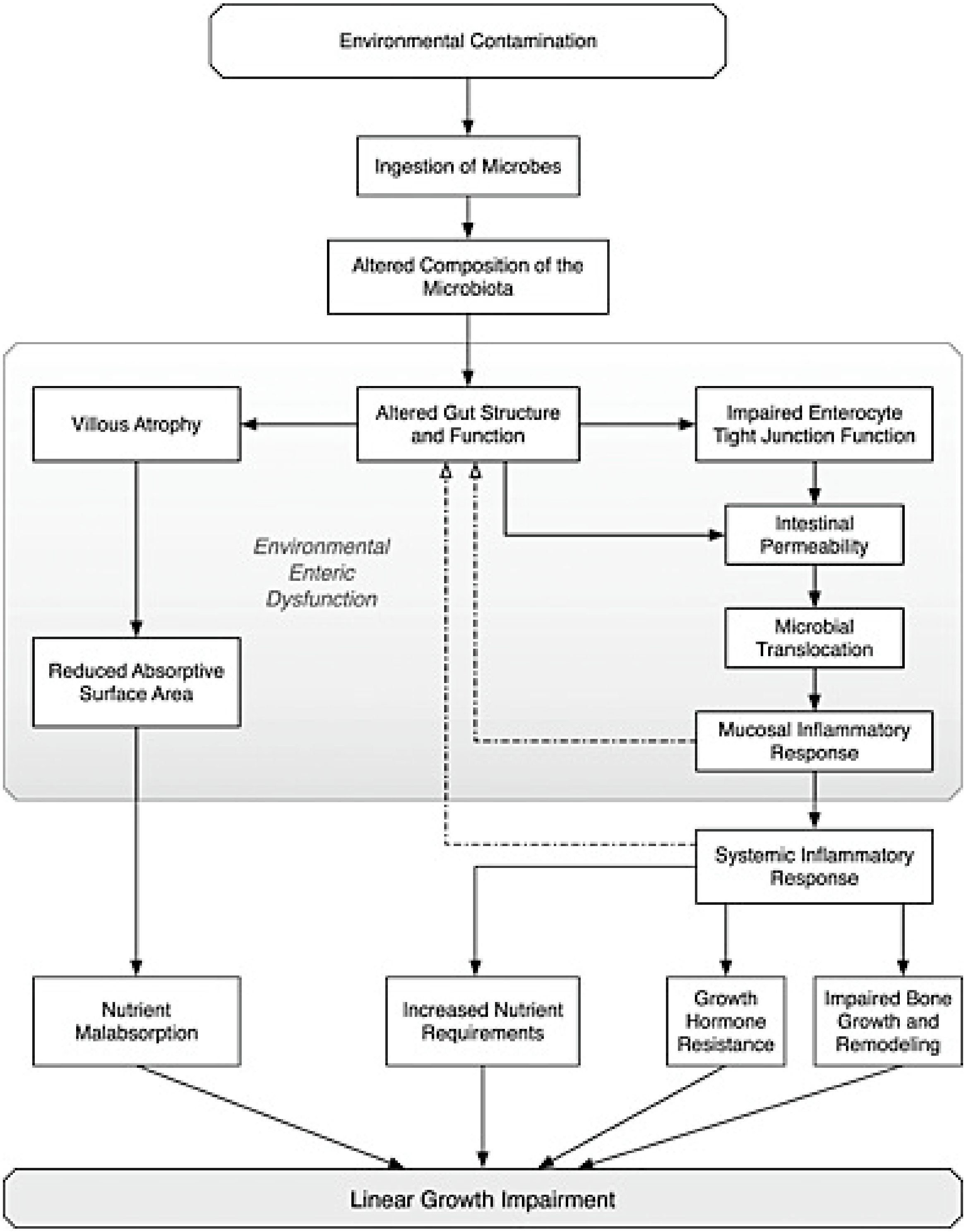

11. Prendergast AJ, Kelly P: **Interactions between intestinal pathogens, enteropathy and malnutrition in developing countries**. 2016, 29(3):229-236. doi: 10.1097/QCO.0000000000000261

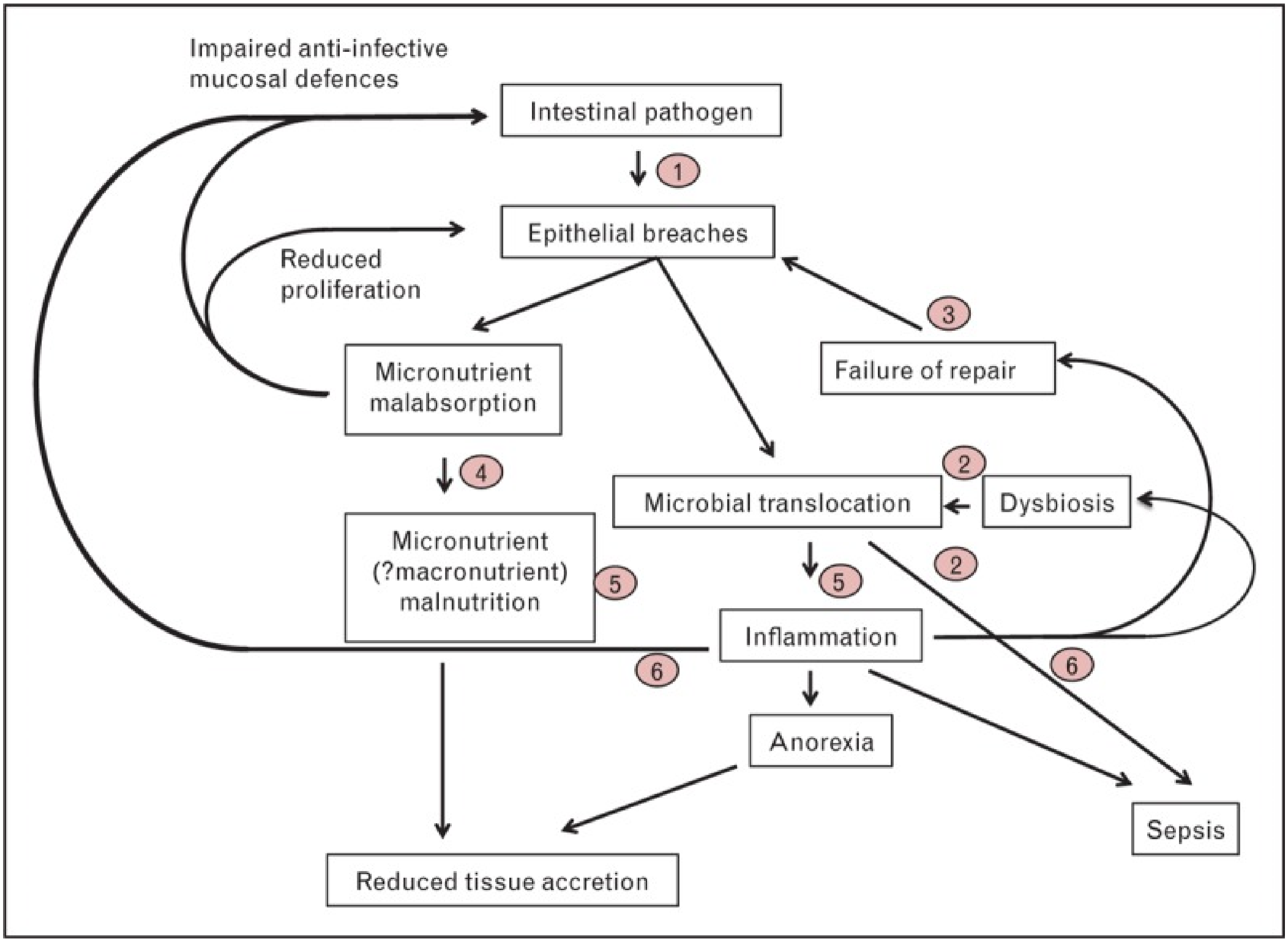

12. Raihan MJ, Farzana FD, Sultana S, Haque MA, Rahman AS, Waid JL, McCormick B, Choudhury N, Ahmed T: **Examining the relationship between socio-economic status, WASH practices and wasting.** *PloS one* 2017, 12(3):e0172134. https://doi.org/10.1371/journal.pone.0172134

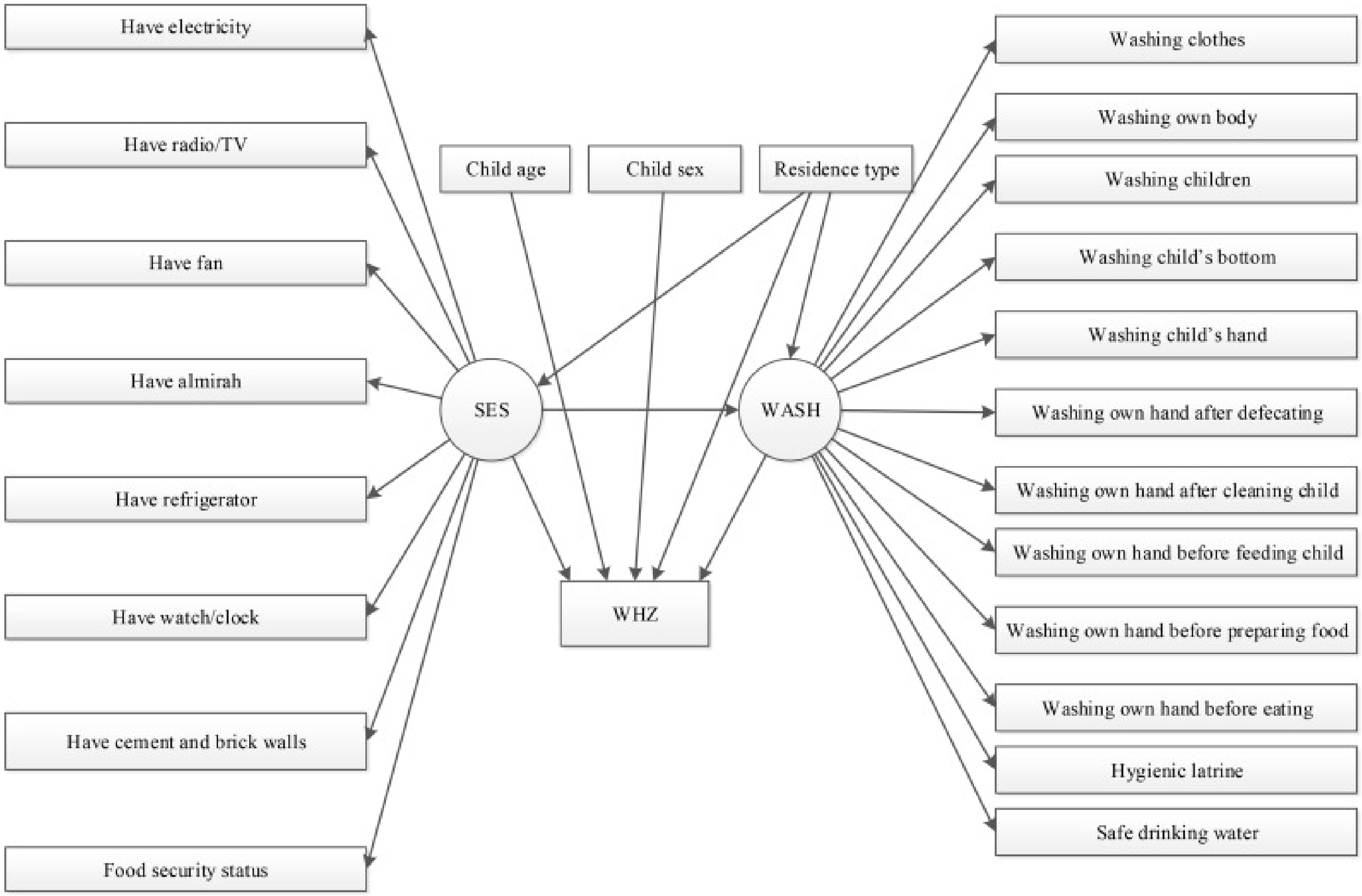

13. World Health Organization: **Guidelines for drinking-water quality: fourth edition incorporating the first addendum**. Geneva: World Health Organization; 2017.

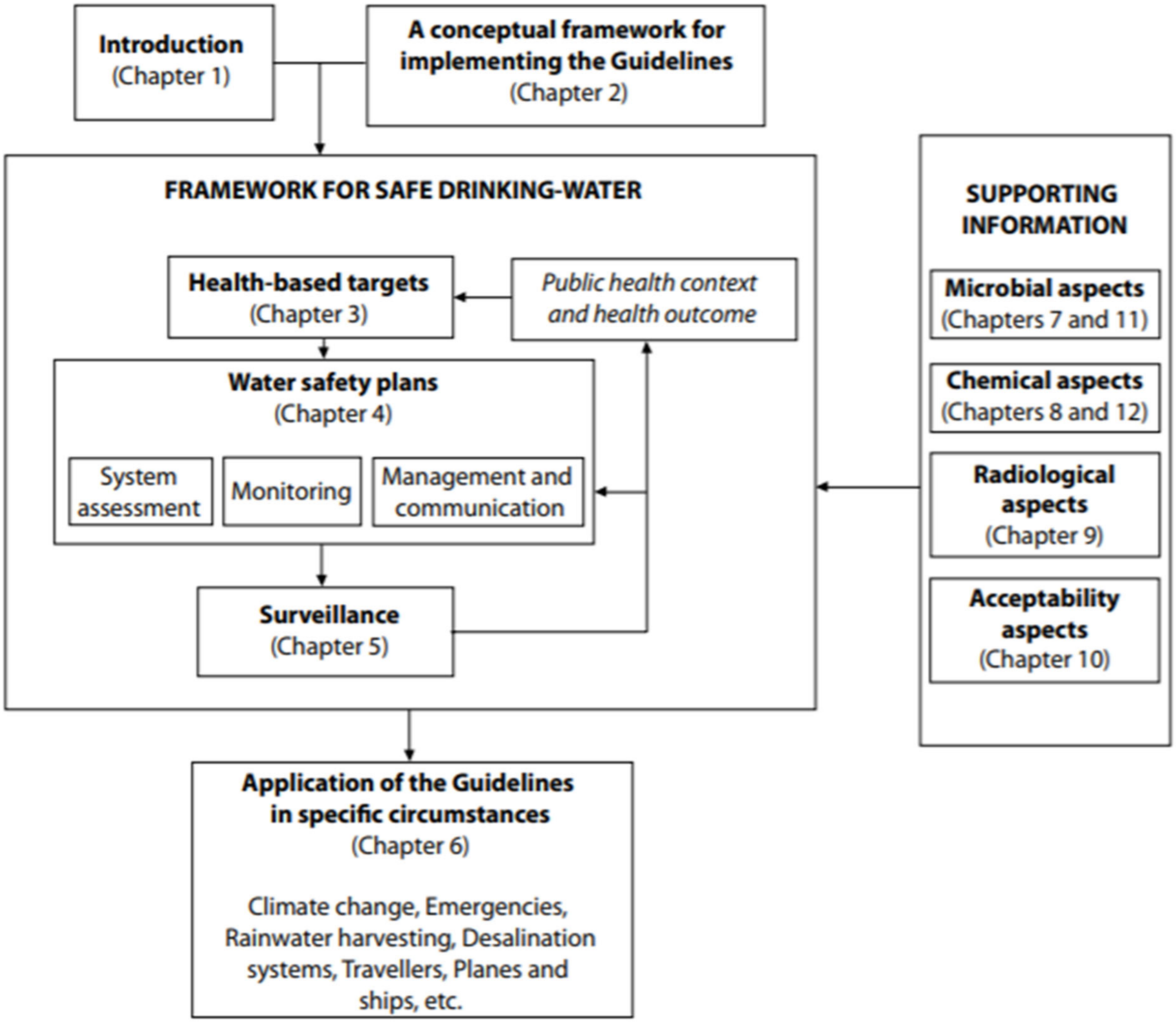

Figure 1.1 Interrelationships among the individual chapters of the *Guidelines for drinking-water quality* in ensuring drinking-water safety

14. World Health Organization. Guidelines on sanitation and health. Geneva: World Health Organization; 2018.

Figure 1.1 **The health impact of unsafe sanitation**

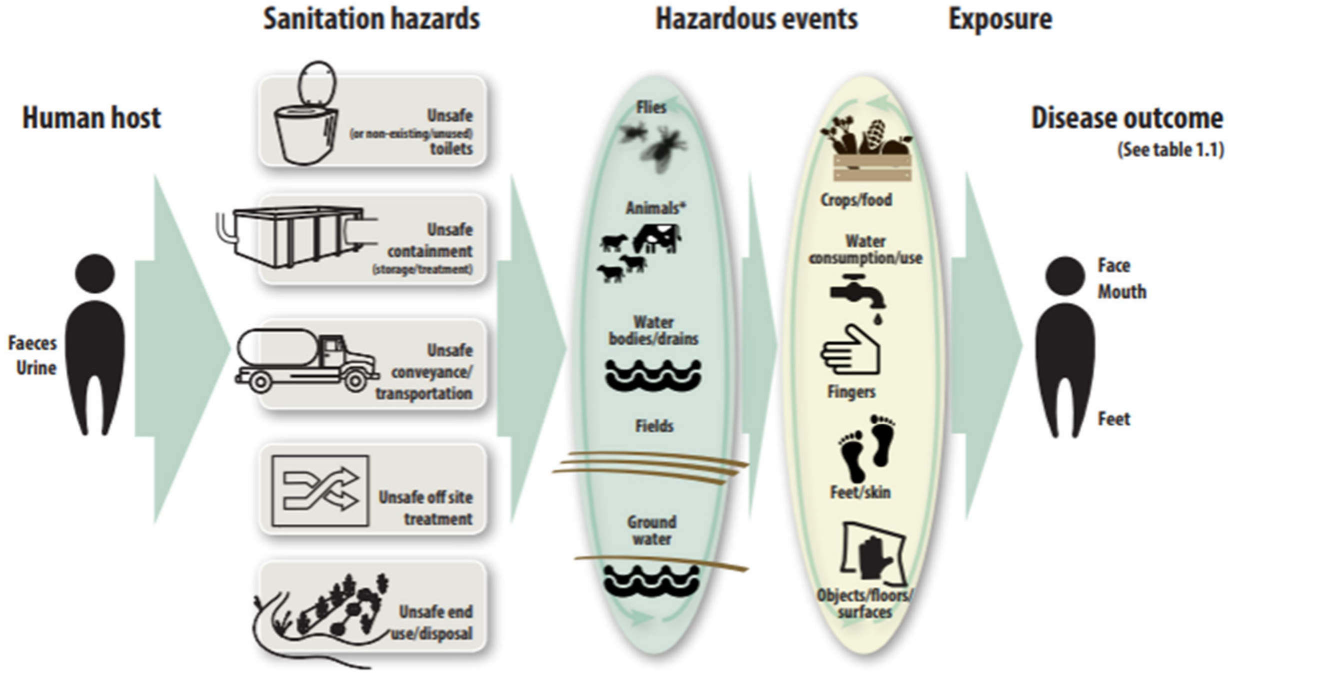

The commonly-used F-diagram on faecal-oral disease transmission (various versions adapted from Wagner and Lanoix, 1958) is not used in these guidelines, although several of its elements can be clearly discerned (human hosts, and the elements described as "hazardous events" in this diagram). The purpose of this figure is to highlight the role of safe sanitation systems as a primary barrier to transmission by showing the way in which unsafe management at each step of the sanitation chain spreads excreta in the environment; additionally, the diagram captures transmission routes that are not faecal-oral and shows the complex ways in which different hazards and hazardous events interrelate. The diagram forms a conceptual basis for risk assessment and management for sanitation systems.

Figure 1.2 **Sanitation service chain**

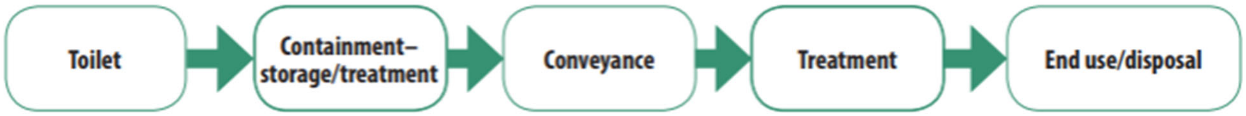

Continued: World Health Organization. **Guidelines on sanitation and health.** Geneva: World Health Organization; 2018.

Figure 3.2 Excreta flow diagram showing examples of hazardous events at each step of the sanitation service chain (adapted from Peal et al., 2014)

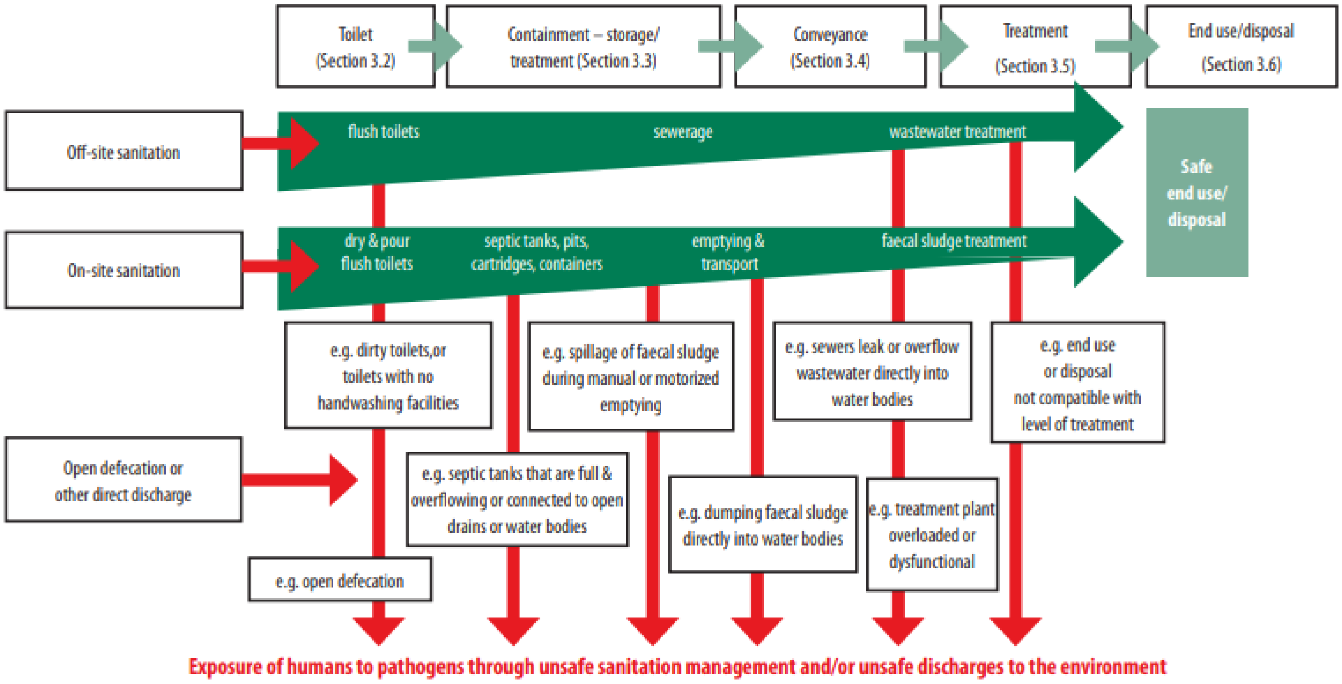

Figure 4.1 Categorization of sanitation services

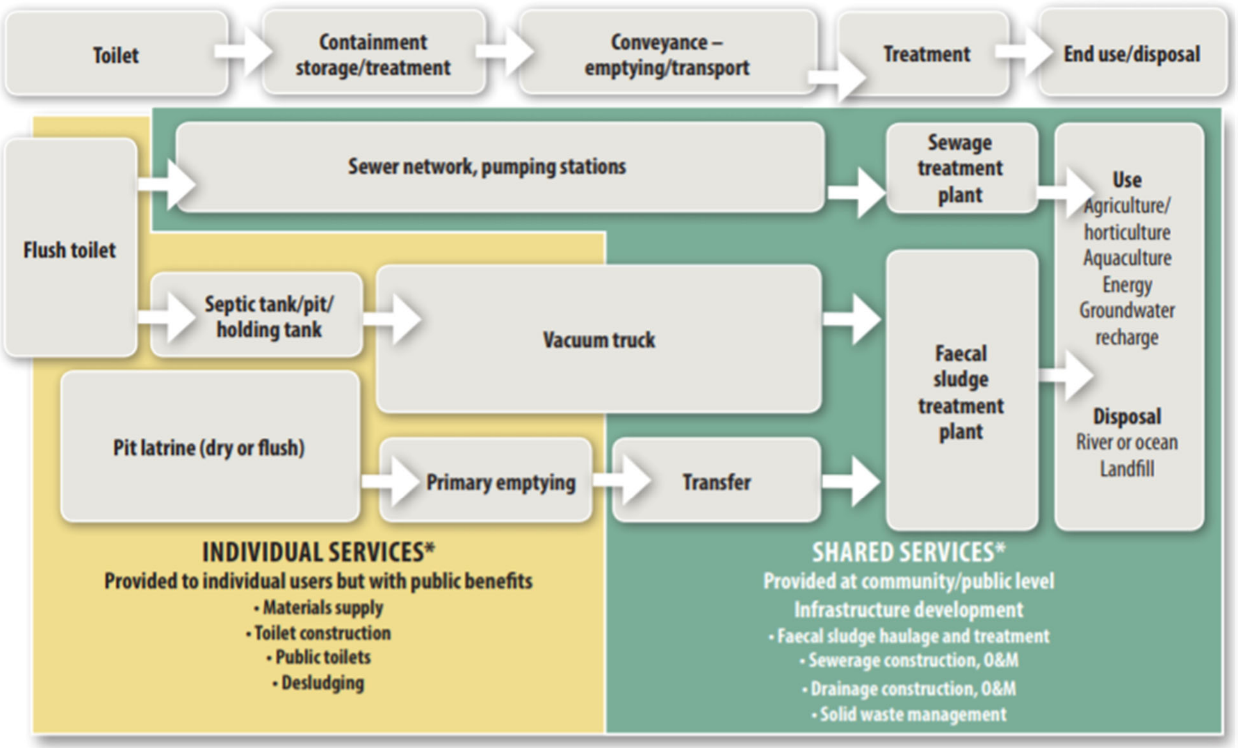

*Delineation of individual services and shared services in this diagram does not signify who should bear the full cost of services

Continued: World Health Organization. **Guidelines on sanitation and health.** Geneva: World Health Organization; 2018.

Figure 4.2 Implementation framework for sanitation

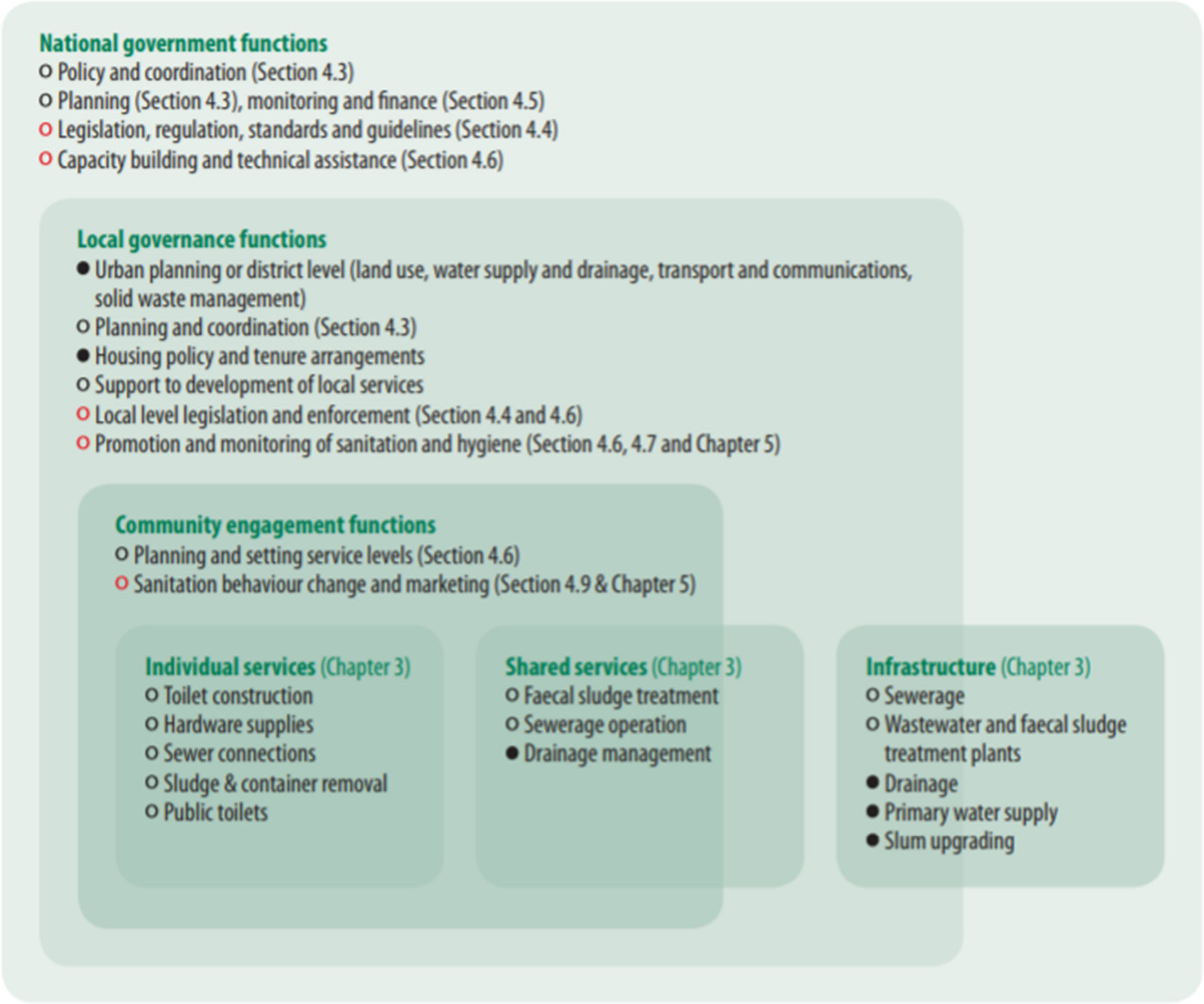

o Function addressed in these guidelines; ● Function not addressed in these guidelines; o Function with primary role for environmental health staff.
This figure indicates how the different levels of the implementation framework interact with each other, and the services and infrastructure that they should deliver.

Continued: World Health Organization. **Guidelines on sanitation and health.** Geneva: World Health Organization; 2018.

Figure 4.4 Sanitation service chain regulatory mechanism options

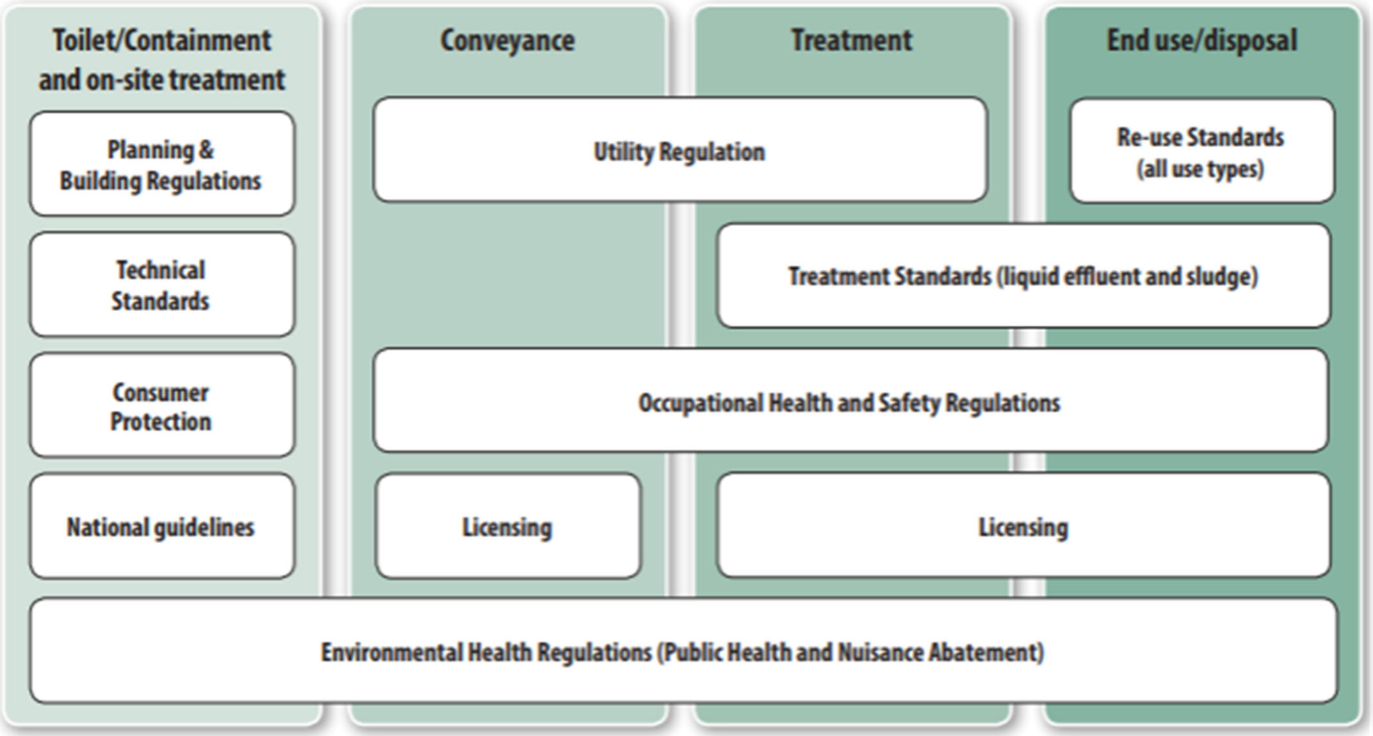

Continued: World Health Organization. **Guidelines on sanitation and health.** Geneva: World Health Organization; 2018.

Figure 7.1 Conceptual framework for guidelines development

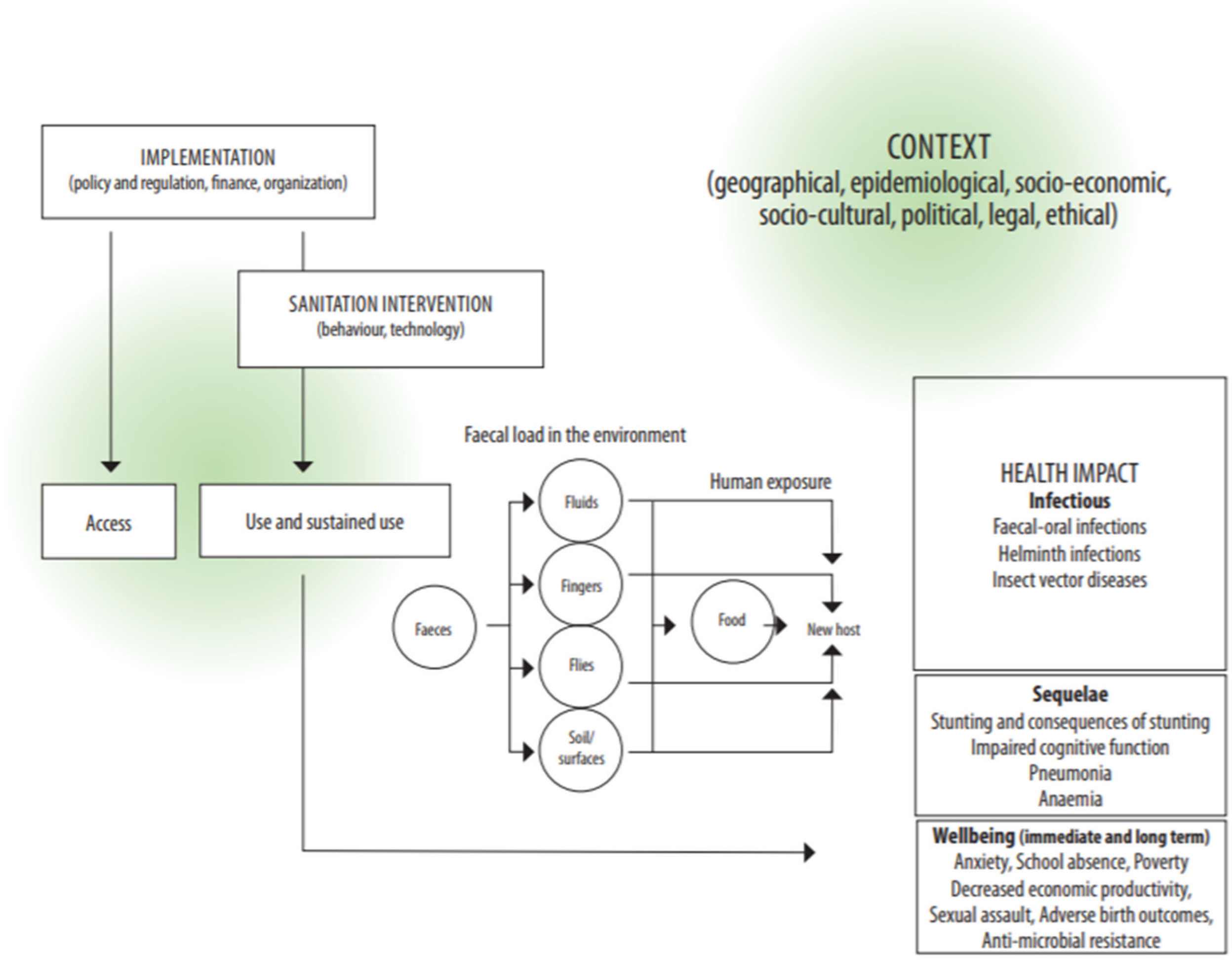

Continued: World Health Organization. **Guidelines on sanitation and health.** Geneva: World Health Organization; 2018.

Figure 8.1 Preliminary conceptual framework of the influence of inadequate sanitation on well-being

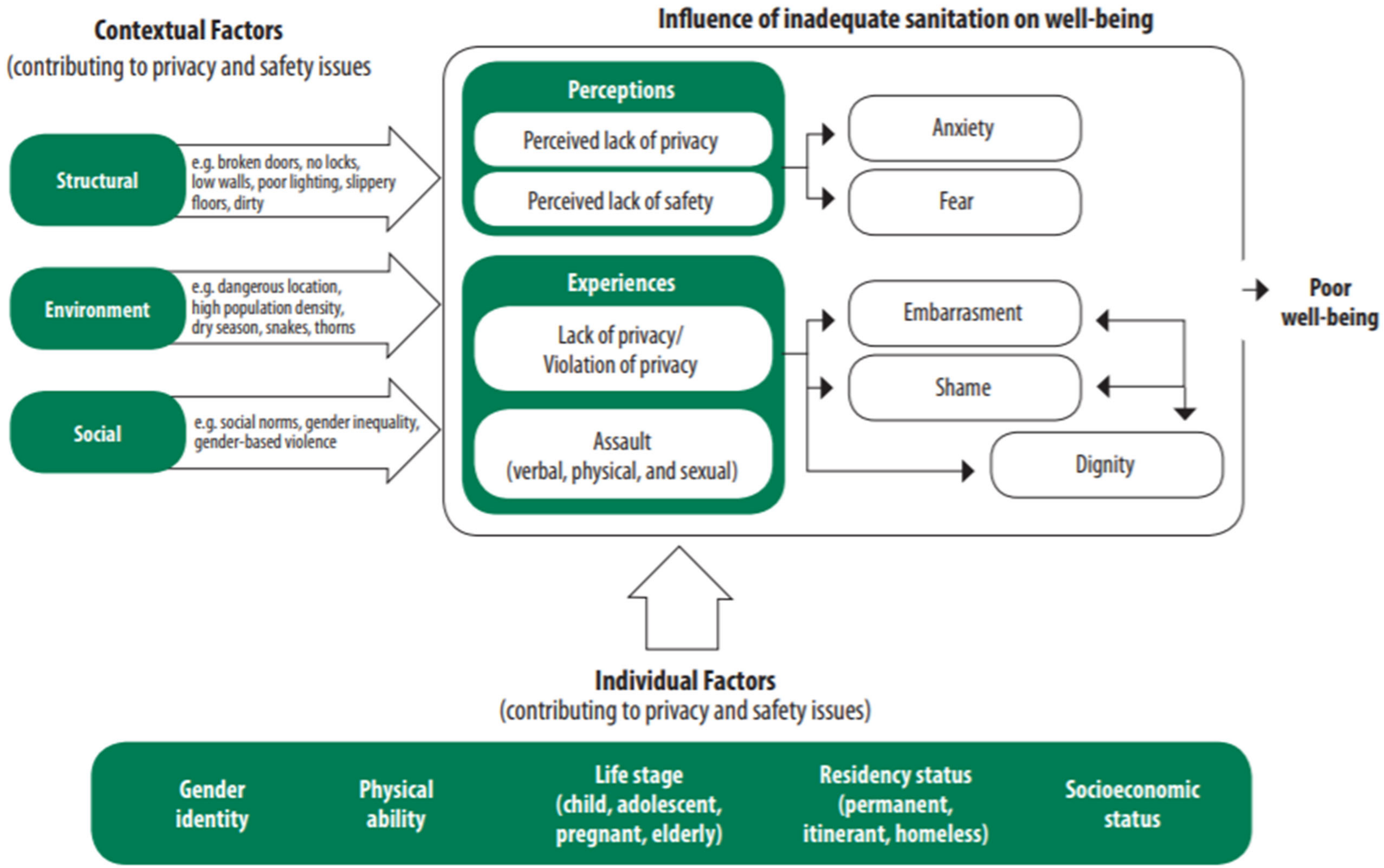

Continued: World Health Organization. **Guidelines on sanitation and health**. Geneva: World Health Organization; 2018.

Box 1.2 Human right to sanitation (UN, 2015a)

The human right to sanitation entitles everyone to sanitation services that provide privacy and ensure dignity, and that are physically accessible and affordable, safe, hygienic, secure, socially and culturally acceptable. Human rights principles must be applied in the context of realising all human rights, including the human right to sanitation:

1. **Non-discrimination and equality:** All people must be able to access adequate sanitation services, without discrimination, prioritizing the most vulnerable and disadvantaged individuals and groups.
2. **Participation:** Everyone must be able to participate in decisions relating to their access to sanitation without discrimination.
3. **The right to information:** Information relating to access to sanitation, including planned programmes and projects must be freely available to those who will be affected, in relevant languages and through appropriate media.
4. **Accountability (monitoring and access to justice):** States must be able to be held to account for any failure to ensure access to sanitation, and access (and lack of access) must be monitored.
5. **Sustainability:** Access to sanitation must be financially and physically sustainable, including in the long-term.

The normative content of the human right to sanitation is defined by:

1. **Availability:** A sufficient number of sanitation facilities must be available for all individuals.
2. **Accessibility:** Sanitation services must be accessible to everyone within, or in the immediate vicinity, of household, health and educational institution, public institutions and places and workplace. Physical security must not be threatened when accessing facilities.
3. **Quality:** Sanitation facilities must be hygienically and technically safe to use. To ensure good hygiene, access to water for cleansing and handwashing at critical times is essential.
4. **Affordability:** The price of sanitation and services must be affordable for all without compromising the ability to pay for other essential necessities guaranteed by human rights such as water, food, housing and health care.
5. **Acceptability:** Services, in particular sanitation facilities, have to be culturally acceptable. This will often require gender-specific facilities, constructed to ensure privacy and dignity.

All human rights are interlinked and mutually reinforcing, and no human right takes precedence over another.

15. Jones KD, Thitiri J, Ngari M, Berkley JA: **Childhood Malnutrition: Toward an Understanding of Infections, Inflammation, and Antimicrobials.** *Food and Nutrition Bulletin* 2014, 35(2_suppl1):S64-S70. https://doi.org/10.1177/15648265140352S110

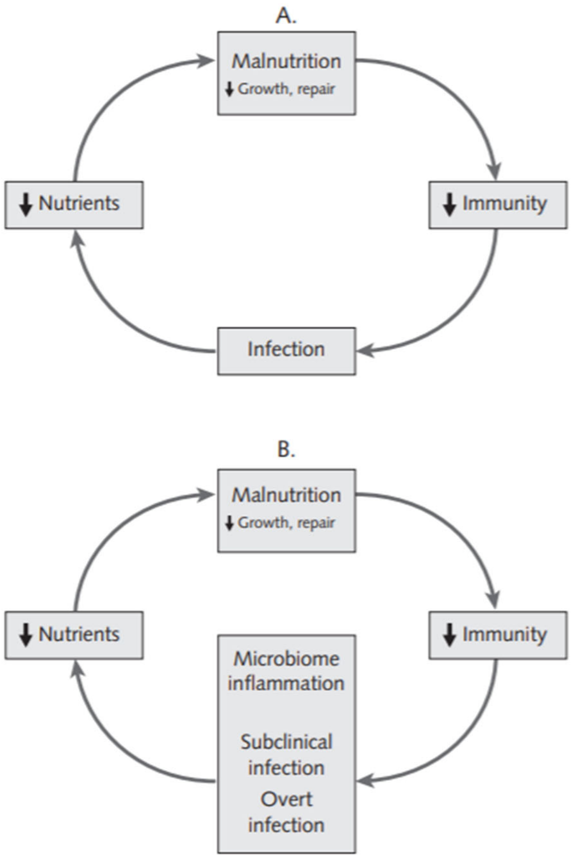

FIG. 1. A. Historical view of the vicious cycle of malnutrition and infection. B. Our current understanding also involves subclinical infection, intestinal inflammation, and altered gut microbiota

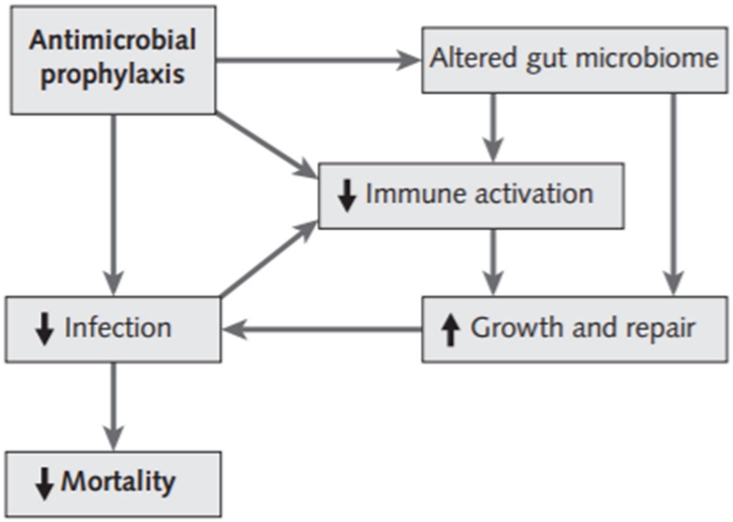

FIG. 3. Hypothesis underlying a randomized, double-blind, controlled trial of daily cotrimoxazole prophylaxis following stabilization in HIV-uninfected children with complicated severe acute malnutrition (SAM)

16. Humphrey JH: Child undernutrition, tropical enteropathy, toilets, and handwashing. *The Lancet* 2009, 374(9694):1032-1035. https://doi.org/10.1016/S0140-6736(09)60950-8

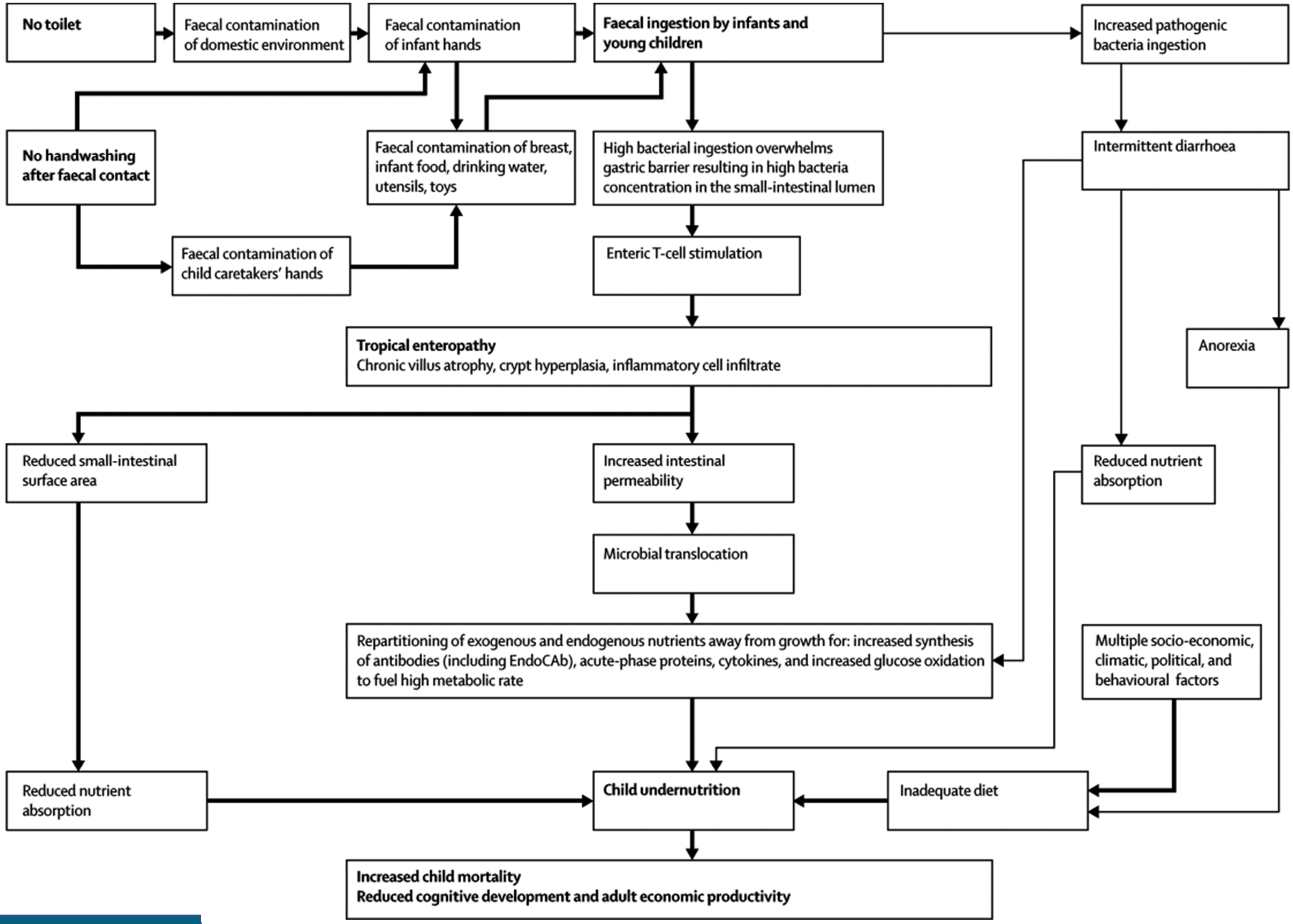

17. Dreibelbis R, Winch PJ, Leontsini E, Hulland KRS, Ram PK, Unicomb L, Luby SP: **The Integrated Behavioural Model for Water, Sanitation, and Hygiene: a systematic review of behavioural models and a framework for designing and evaluating behaviour change interventions in infrastructure-restricted settings.** *BMC Public Health* 2013, 13(1):1015. https://doi.org/10.1186/1471-2458-13-1015

Levels	Contextual factors	Psychosocial factors	Technology factors
**Societal/Structural**	Policy and regulations, climate and geography	Leadership/advocacy, cultural identity	Manufacturing, financing, and distribution of the product; current and past national policies and promotion of products
**Community**	Access to markets, access to resources, built and physical environment	Shared values, collective efficacy, social integration, stigma	Location, access, availability, individual vs. collective ownership/access, and maintenance of the product
**Interpersonal/Household**	Roles and responsibilities, household structure, division of labour, available space	Injunctive norms, descriptive norms, aspirations, shame, nurture	Sharing of access to product, modelling/demonstration of use of product
**Individual**	Wealth, age, education, gender, livelihoods/employment	Self-efficacy, knowledge, disgust, perceived threat	Perceived cost, value, convenience, and other strengths and weaknesses of the product
**Habitual**	Favourable environment for habit formation, opportunity for and barriers to repetition of behaviour	Existing water and sanitation habits, outcome expectations	Ease/Effectiveness of routine use of product

18. Mosler H-J: A systematic approach to behavior change interventions for the water and sanitation sector in developing countries: A conceptual model, a review, and a guideline. *International journal of environmental health research* 2012, 22:431-449. DOI: 10.1080/09603123.2011.650156

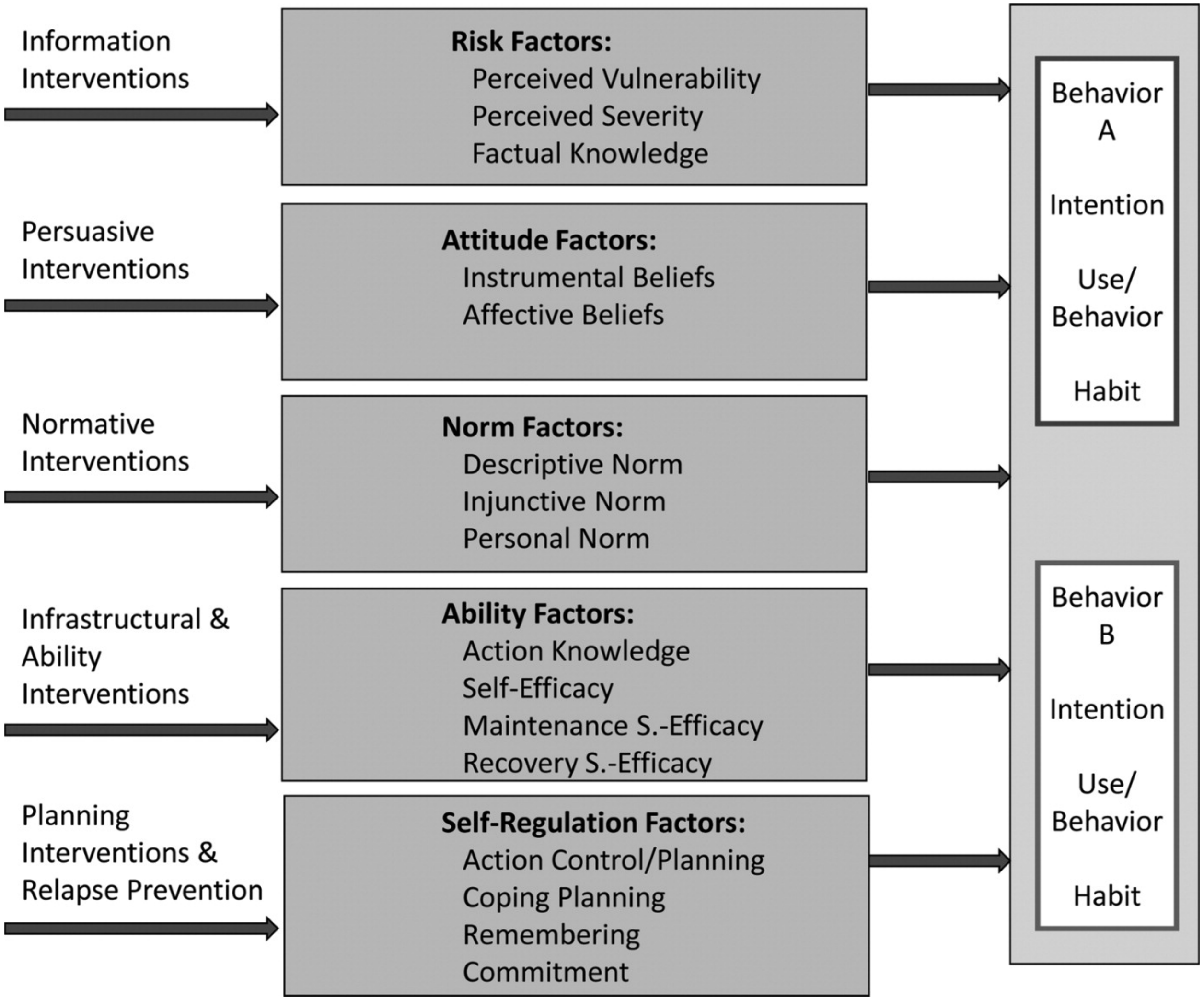

19. Nguyen-Viet H, Zinsstag J, Schertenleib R, Zurbrügg C, Obrist B, Montangero A, Surkinkul N, Koné D, Morel A, Koottatep T *et al*: Improving Environmental Sanitation, Health, and Well-Being: A Conceptual Framework for Integral Interventions. *EcoHealth* 2009, 6:180-191.

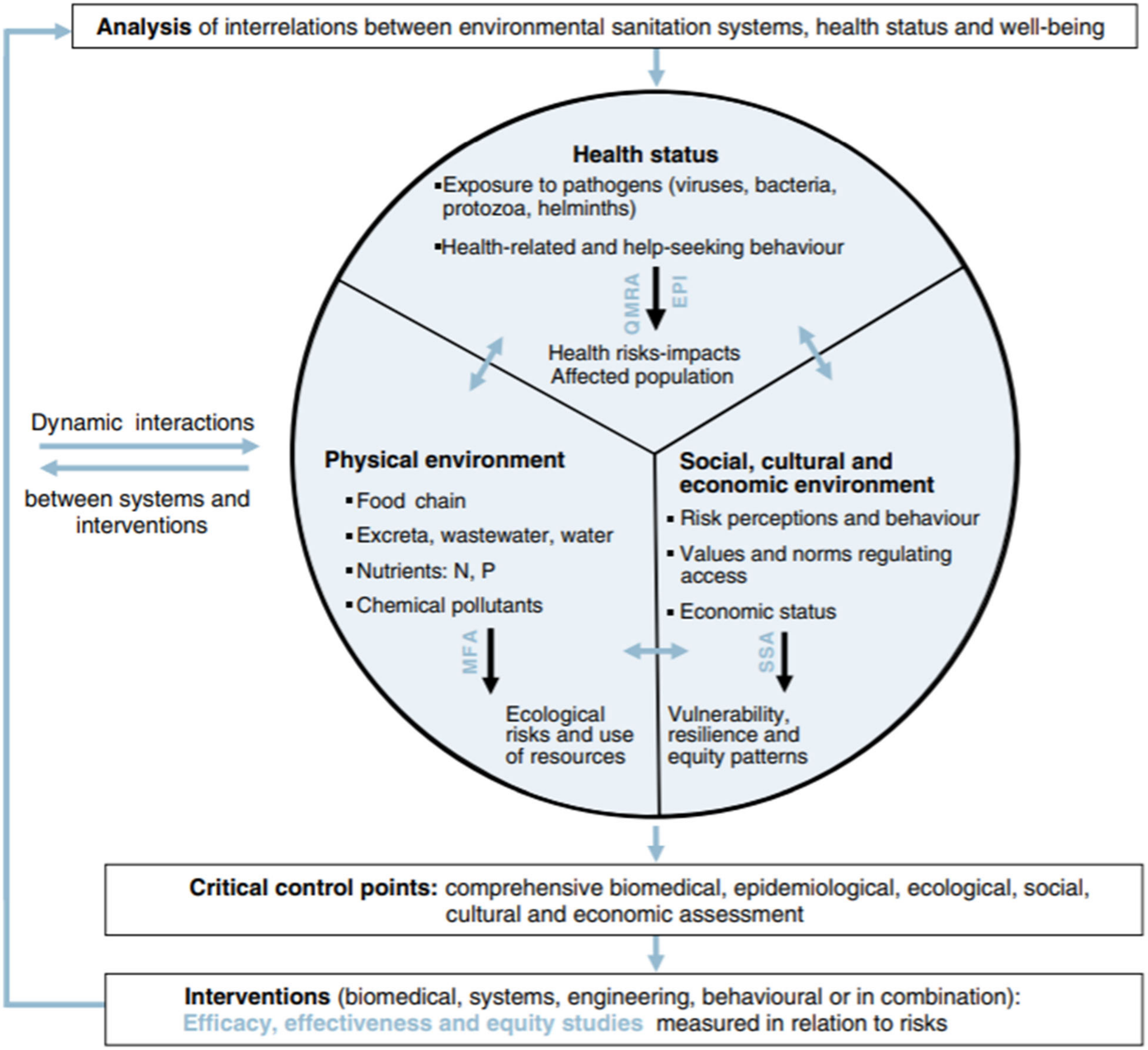

Fig. 1. Conceptual framework of the combination of health and environmental risk assessment for health and environmental sanitation planning. Green characters refer to methodologies used within the conceptual framework (see text for details). *QMRA* quantitative microbial risk assessment, *EPI* epidemiology, *MFA* material flow analysis, *SSA* social science analysis.

20. Gentry-Shields J, Bartram J: **Human health and the water environment: using the DPSEEA framework to identify the driving forces of disease.** *The Science of the total environment* 2014, 468-469:306-314.

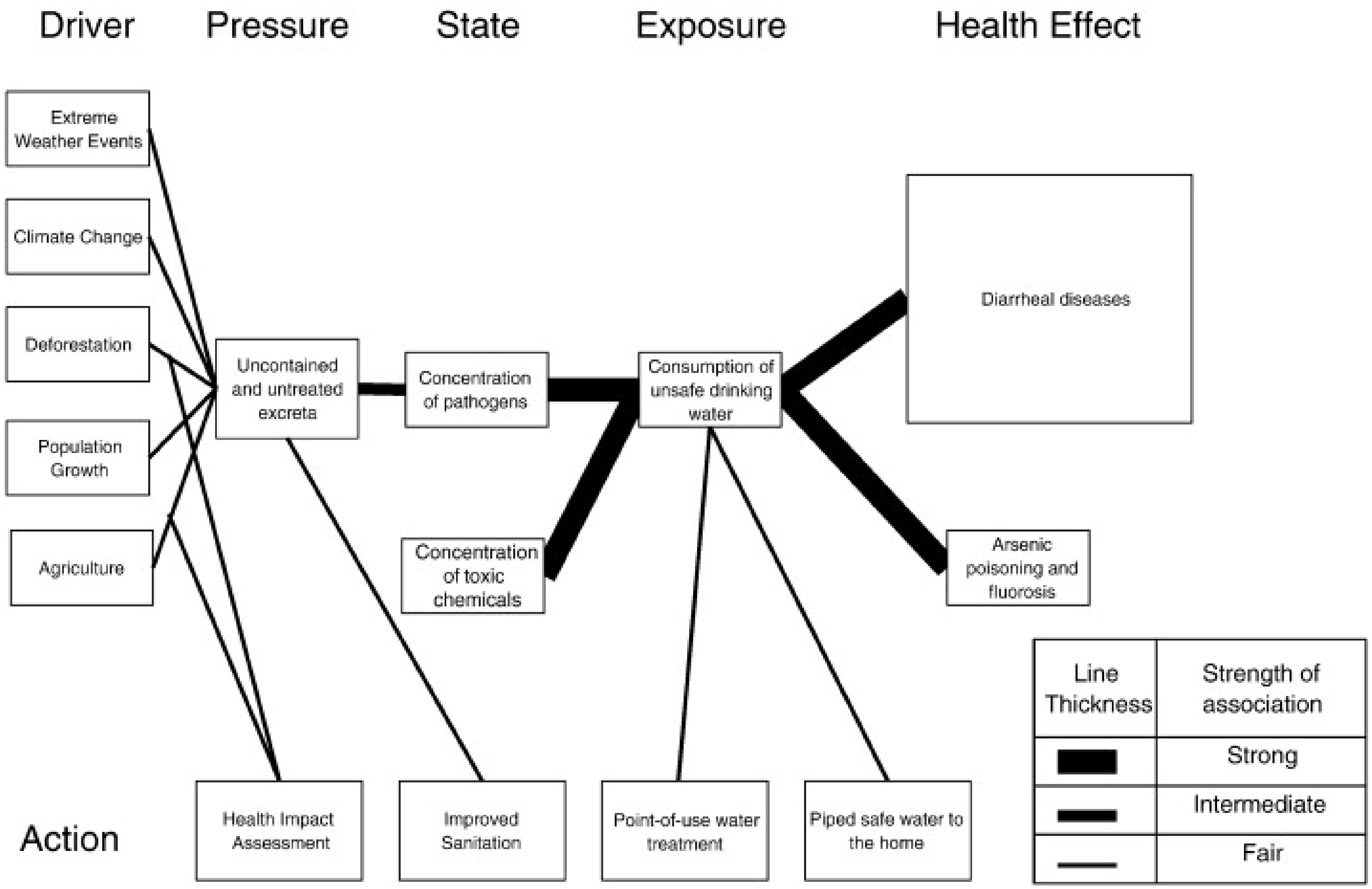

Continued: Gentry-Shields J, Bartram J: **Human health and the water environment: using the DPSEEA framework to identify the driving forces of disease.** The Science of the total environment 2014, 468-469:306-314.

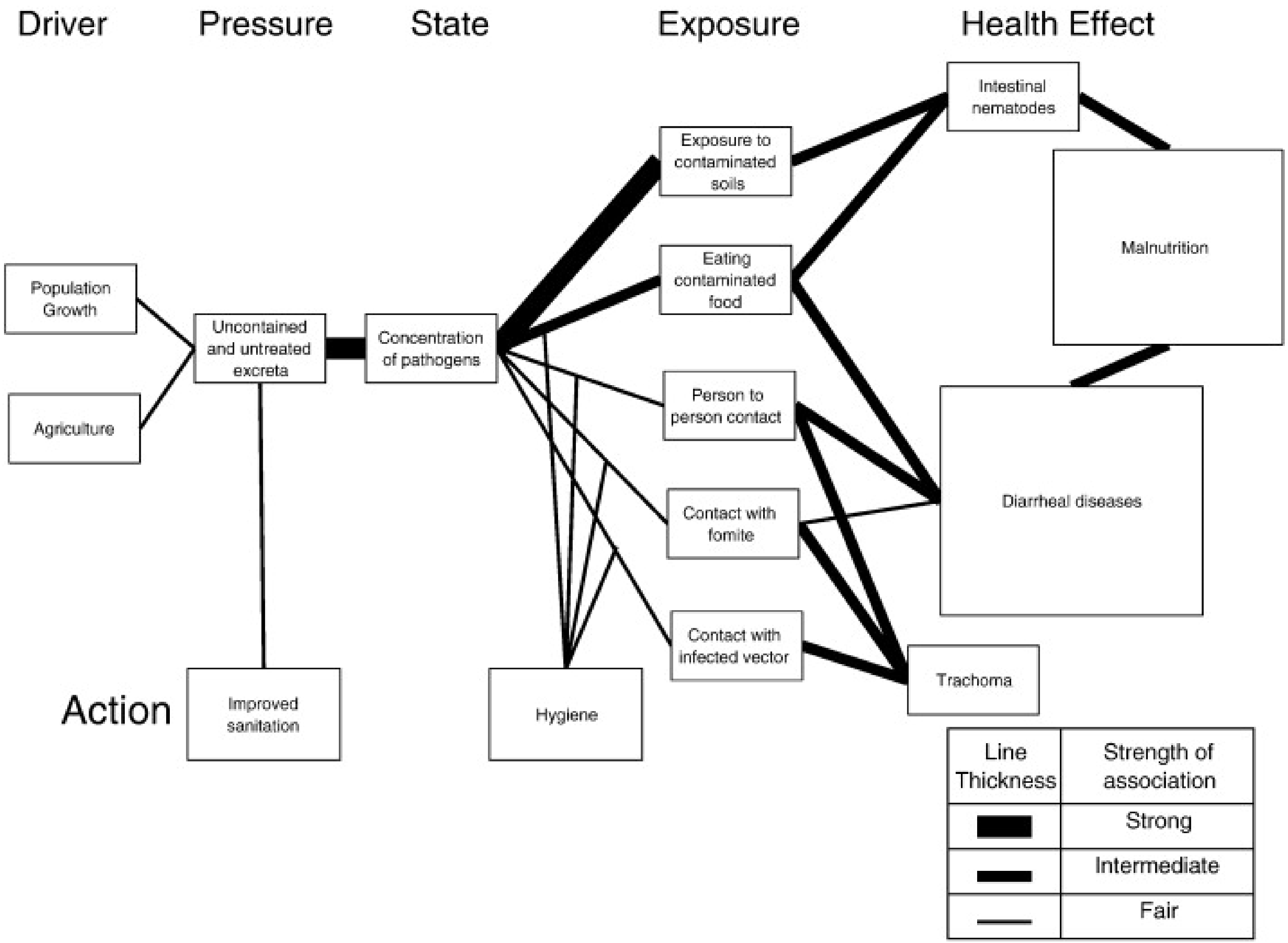

Continued: Gentry-Shields J, Bartram J: **Human health and the water environment: using the DPSEEA framework to identify the driving forces of disease.** The Science of the total environment 2014, 468-469:306-314.

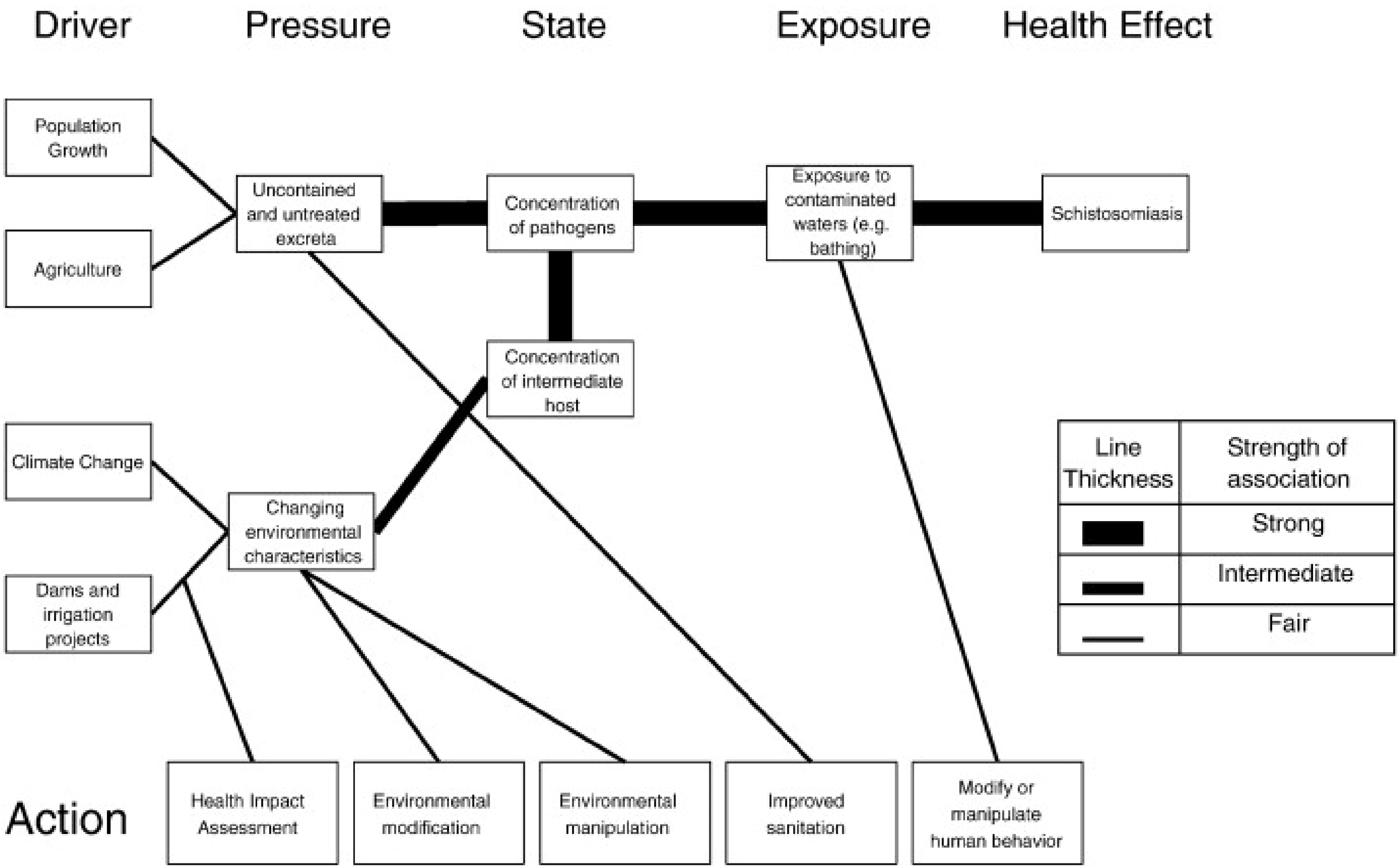

21. Campbell OMR, Benova L, Gon G, Afsana K, Cumming O: **Getting the basic rights - the role of water, sanitation and hygiene in maternal and reproductive health: a conceptual framework**. *Trop Med Int Health* 2015, 20(3):252-267.

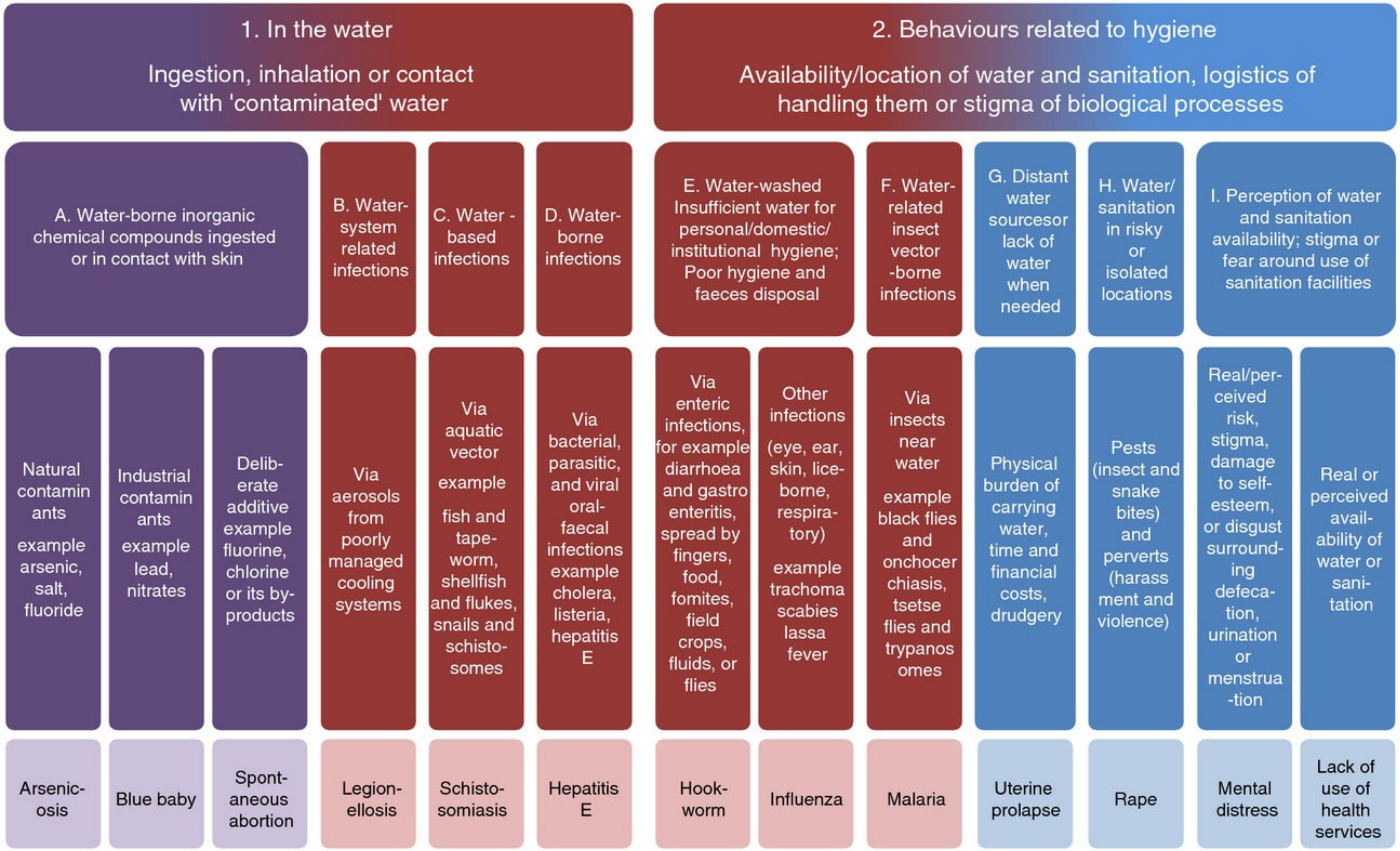

Social Protection

1. Adato M, Bassett L: **Social protection to support vulnerable children and families: The potential of cash transfers to protect education, health and nutrition.** *AIDS Care - Psychol Socio-Medical Asp AIDS/HIV.* 2009;21(SUPPL. 1):60-75. doi:10.1080/09540120903112351

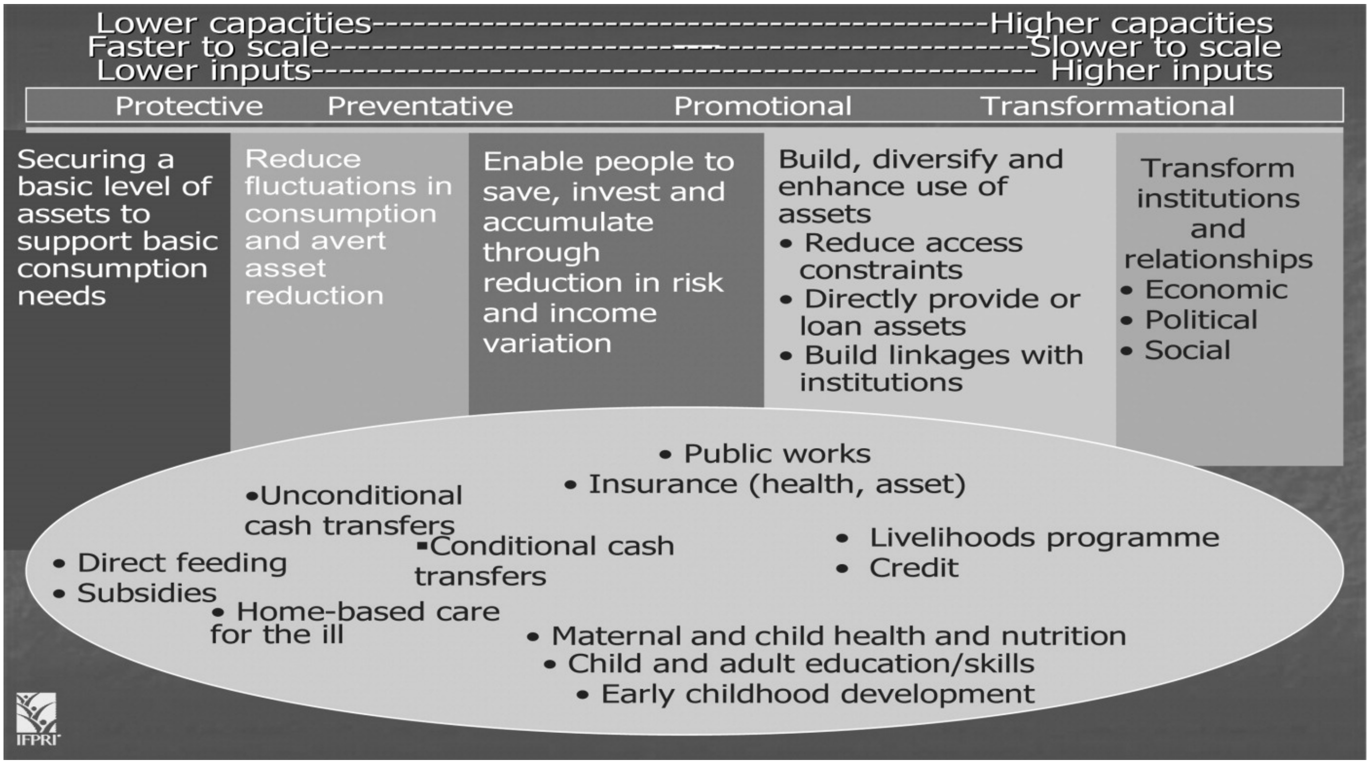

2. Floate HJ, Marks GC, Durham J: Cash transfer programmes in lower-income and middle-income countries: understanding pathways to nutritional change-a realist review protocol. *BMJ Open*. 2019;9:28314. doi:10.1136/bmjopen-2018-028314

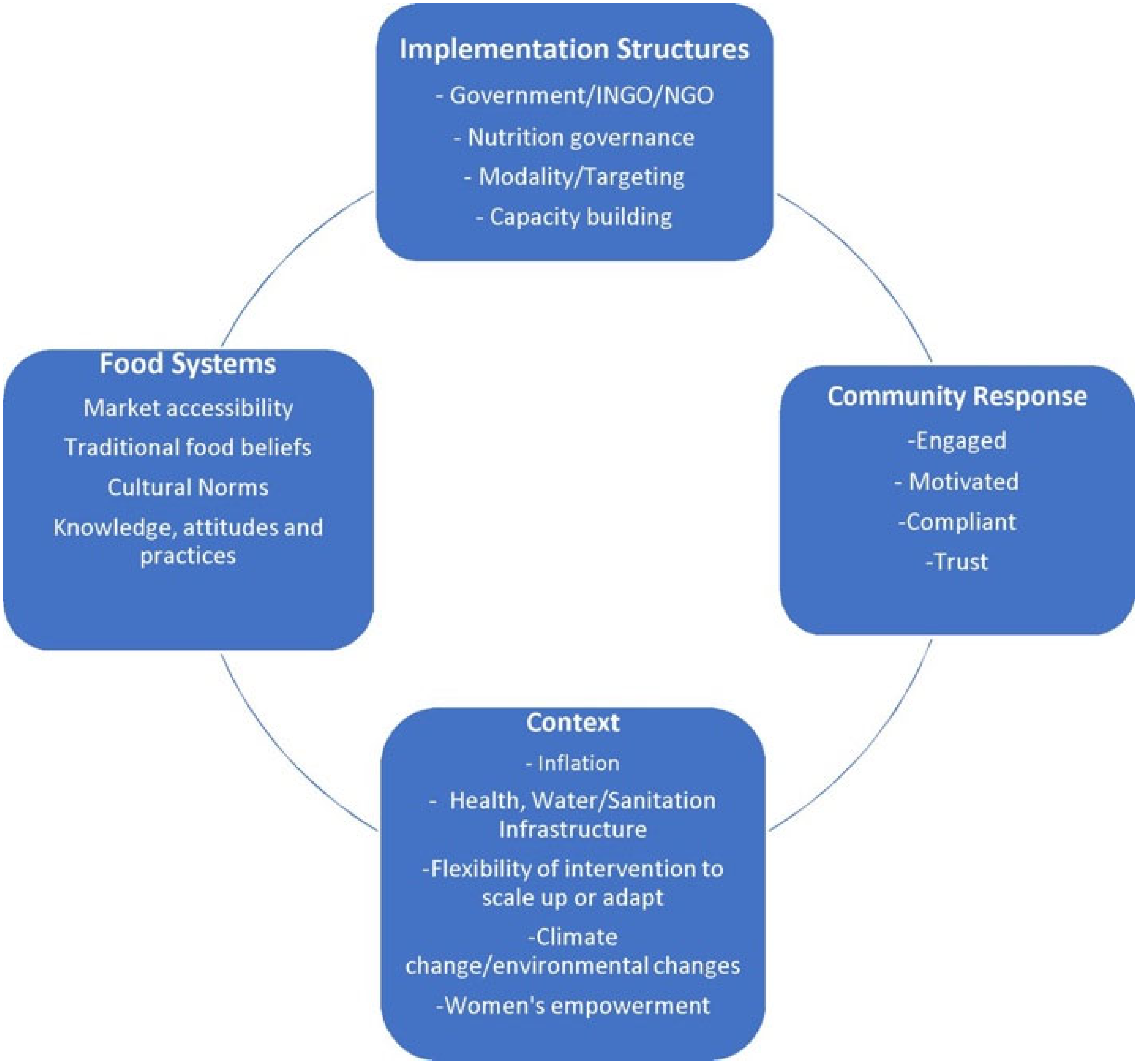

3. Souza D, Chmielewska D: **Public Support To Food Security in India, Brazil and South Africa: Elements for a Policy Dialogue.** 2011. https://ipcig.org/pub/IPCWorkingPaper80.pdf.

FIGURE 4

South Africa's National Integrated Food Security Strategy (IFSS)

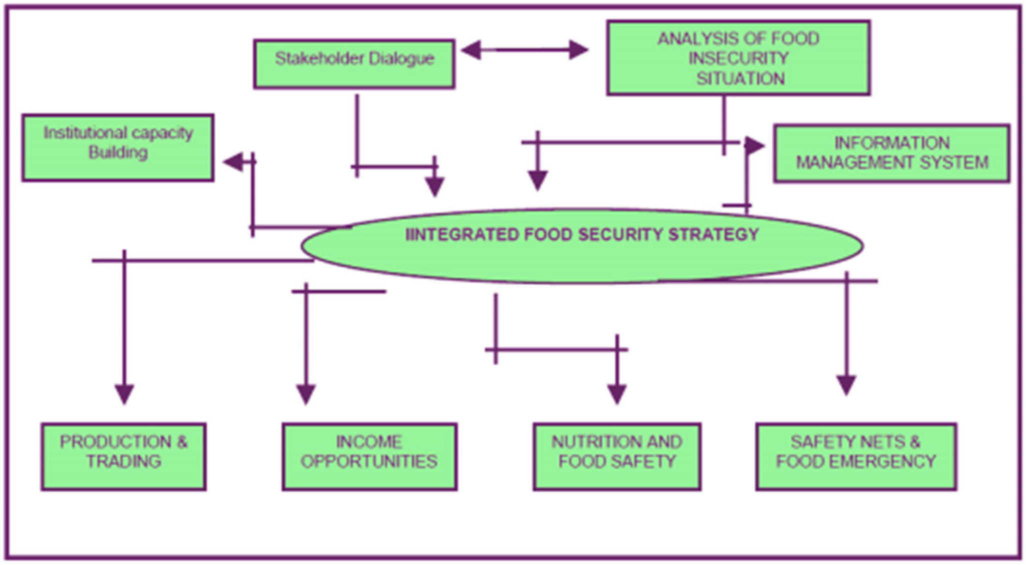

Source: Republic of South Africa (2002).

FIGURE 6

South Africa. Institutional Arrangements and Organisational Structures

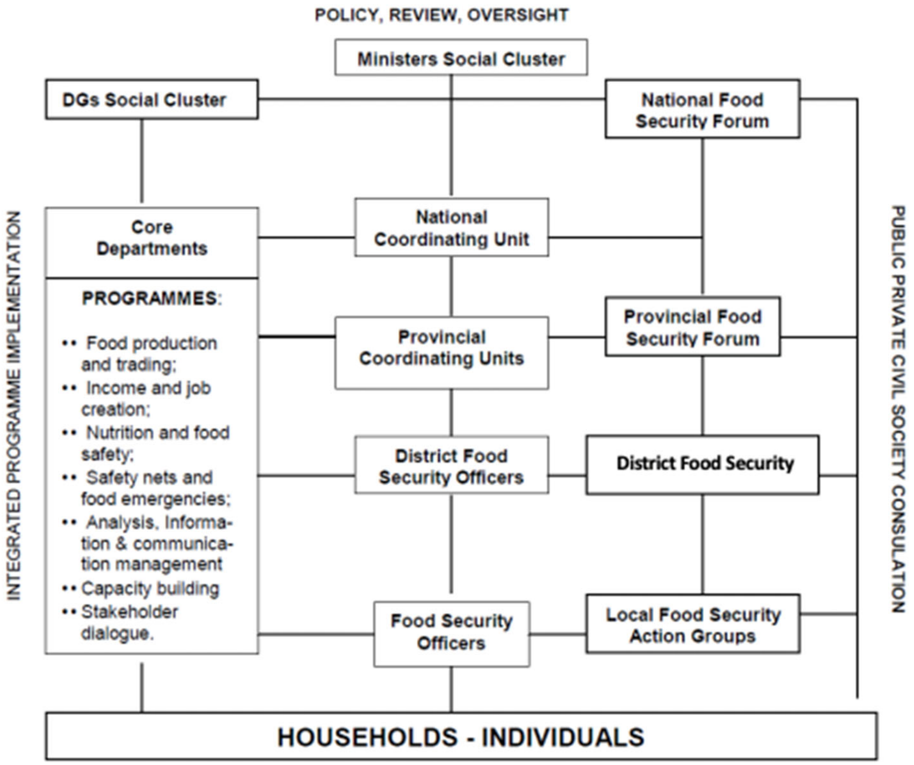

Source: Republic of South Africa (2002).

4. Neufeld: Nutrition in the Oportunidades conditional cash transfer program: Strengths and challenges, presentation at the Third International CCT Conference, Istanbul, 29 June 2006.

FIGURE D-1. POTENTIAL PATHWAYS FOR SOCIAL PROTECTION PROGRAMS TO IMPACT ON NUTRITION

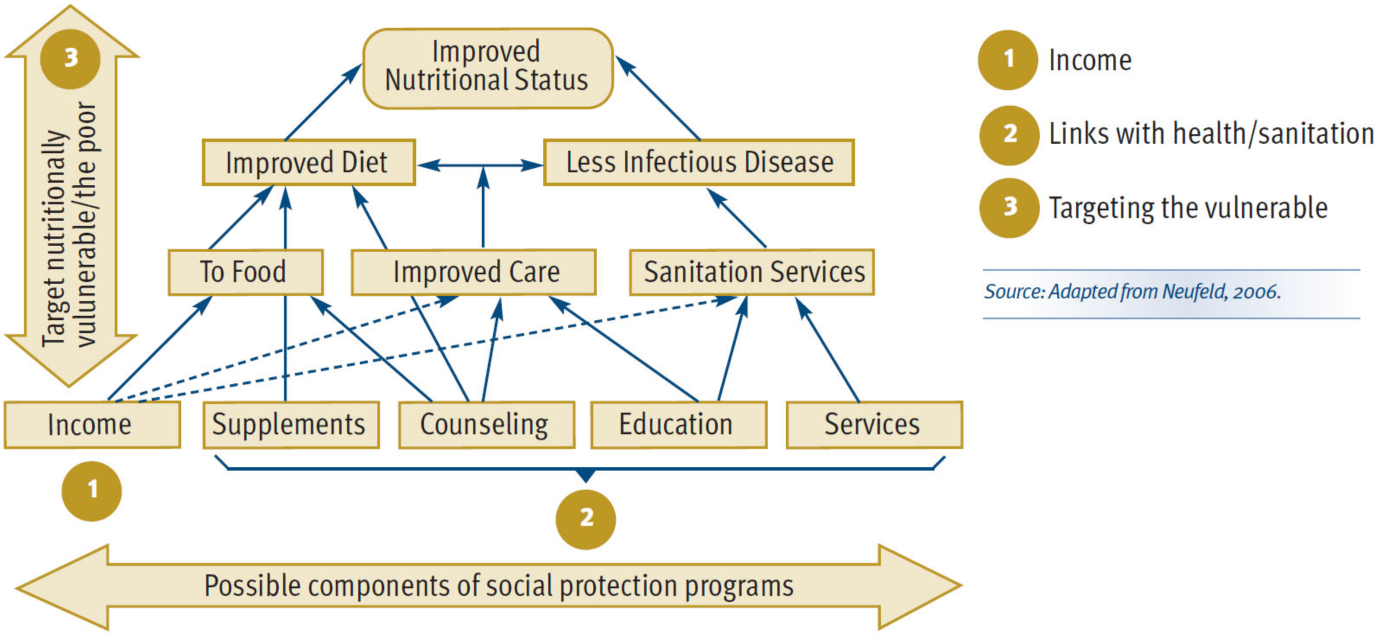

5. World Bank: "Managing Risk, Promoting Growth: Developing Systems for Social Protection in Africa—Africa Social Protection Strategy 2011–2021." Concept Note, World Bank, Washington, DC. 2011.

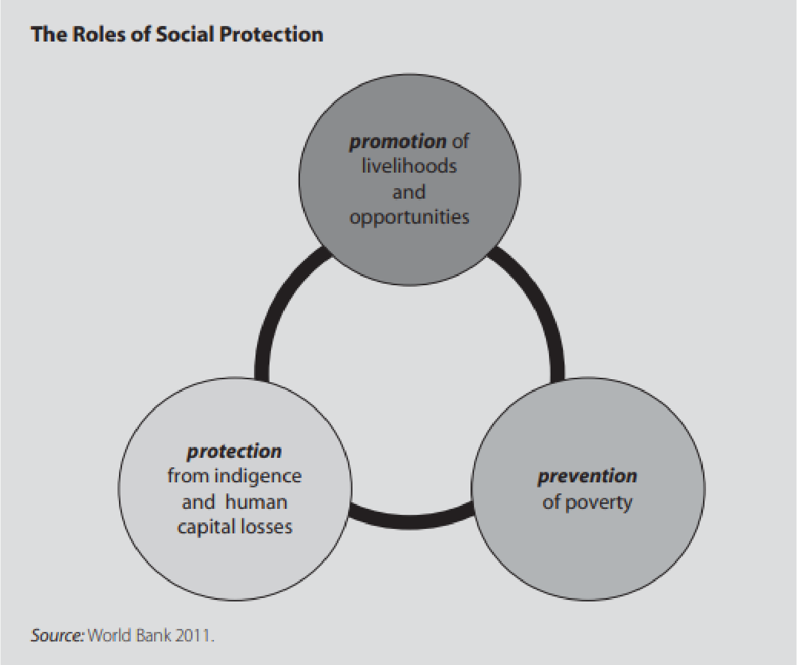

6. Narayanan S, Gerber N: **Social safety nets for food and nutrition security in India.** *Glob Food Sec*. 2017;15:65-76. doi:10.1016/j.gfs.2017.05.001

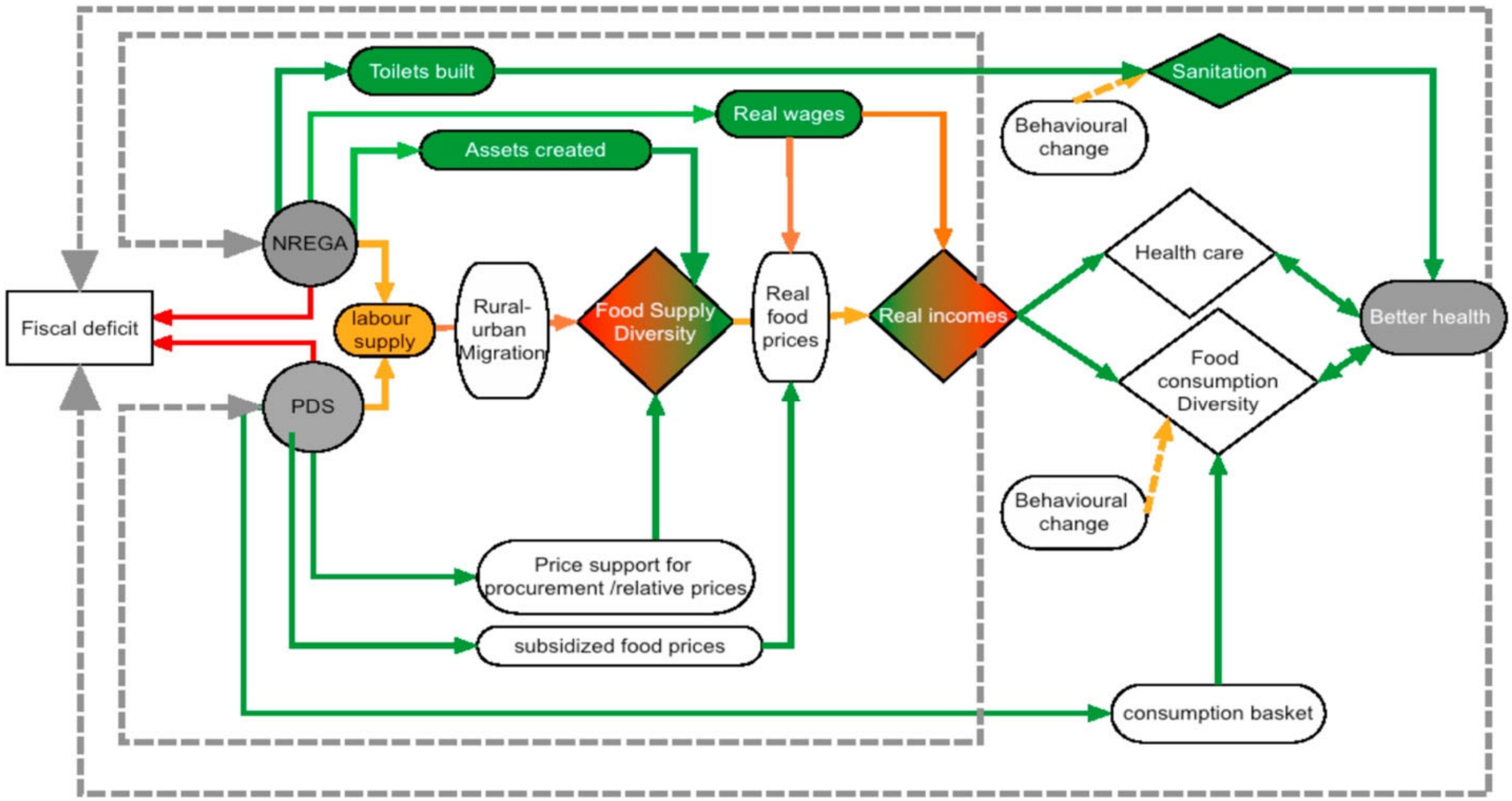

7. Leroy JL, Ruel M, Verhofstadt E: The impact of conditional cash transfer programmes on child nutrition: a review of evidence using a programme theory framework. *J Dev Eff*. 2009;1(2):103-129. doi:10.1080/19439340902924043

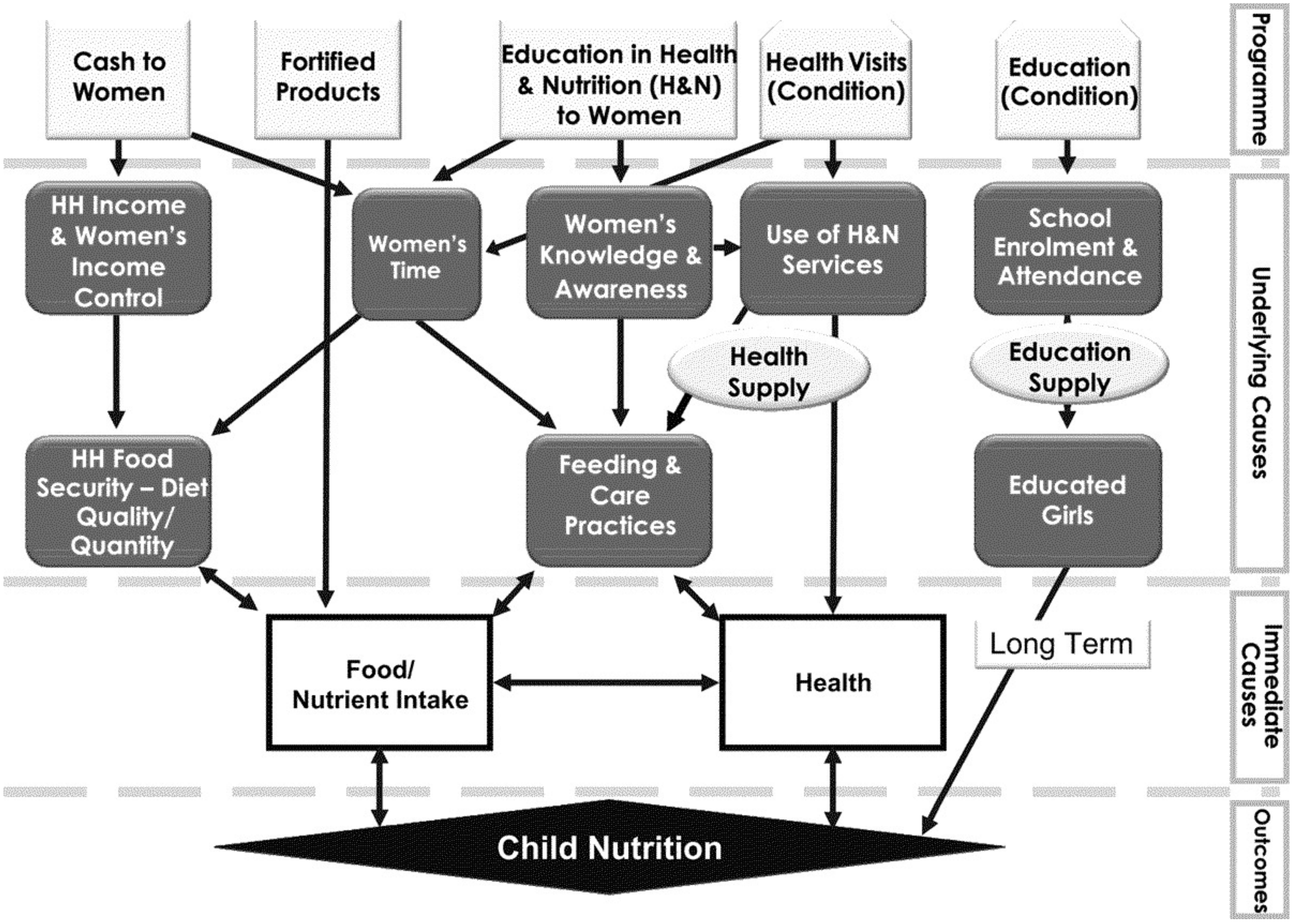

8. de Groot R, Palermo T, Handa S, Ragno LP, Peterman A: Cash Transfers and Child Nutrition: Pathways and Impacts. *Development Policy Review* 2017, 35(5):621-643. doi:10.1111/dpr.12255

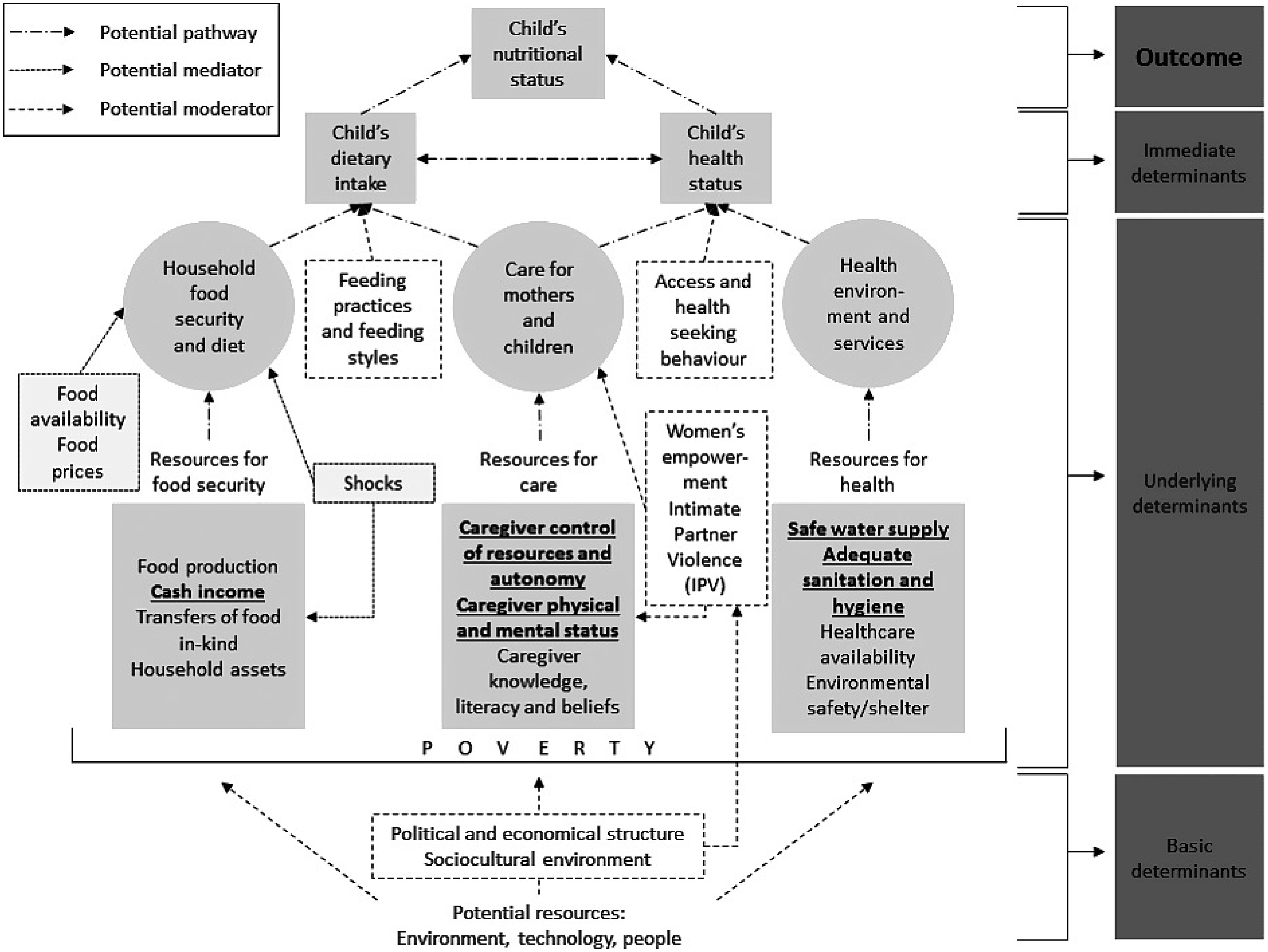

9. Alderman H: **Leveraging Social Protection Programs for Improved Nutrition: Summary of Evidence Prepared for the Global Forum on Nutrition-Sensitive Social Protection Programs,** 2015. Washington, D.C.; 2016. Available from: http://www.securenutrition.org/sites/default/files/resources/attachment/english/Alderman - Global Forum Summary of Evidence - 2016_0.pdf.

FIGURE 5 Indicative Pathways from Social Protection Programs to Nutrition

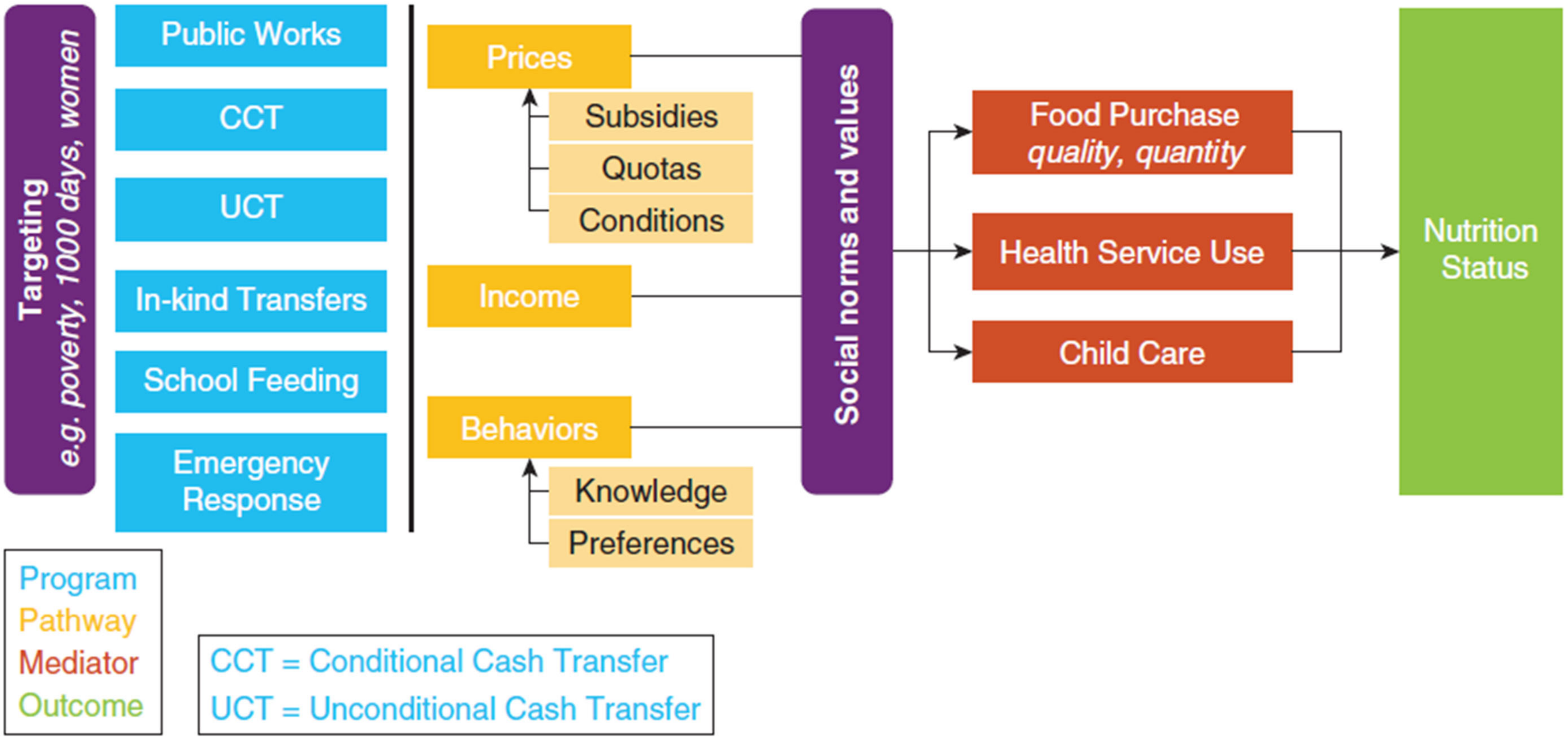

Source: Harold Alderman and SecureNutrition.

10. Olney DK, Marshall Q, Honton G, et al: **Leveraging an Implementation-Research Partnership to Improve Effectiveness of Nutrition-Sensitive Programs at the World Food Programme.** *Food Nutr Bull*. 2019. doi:10.1177/0379572119874273

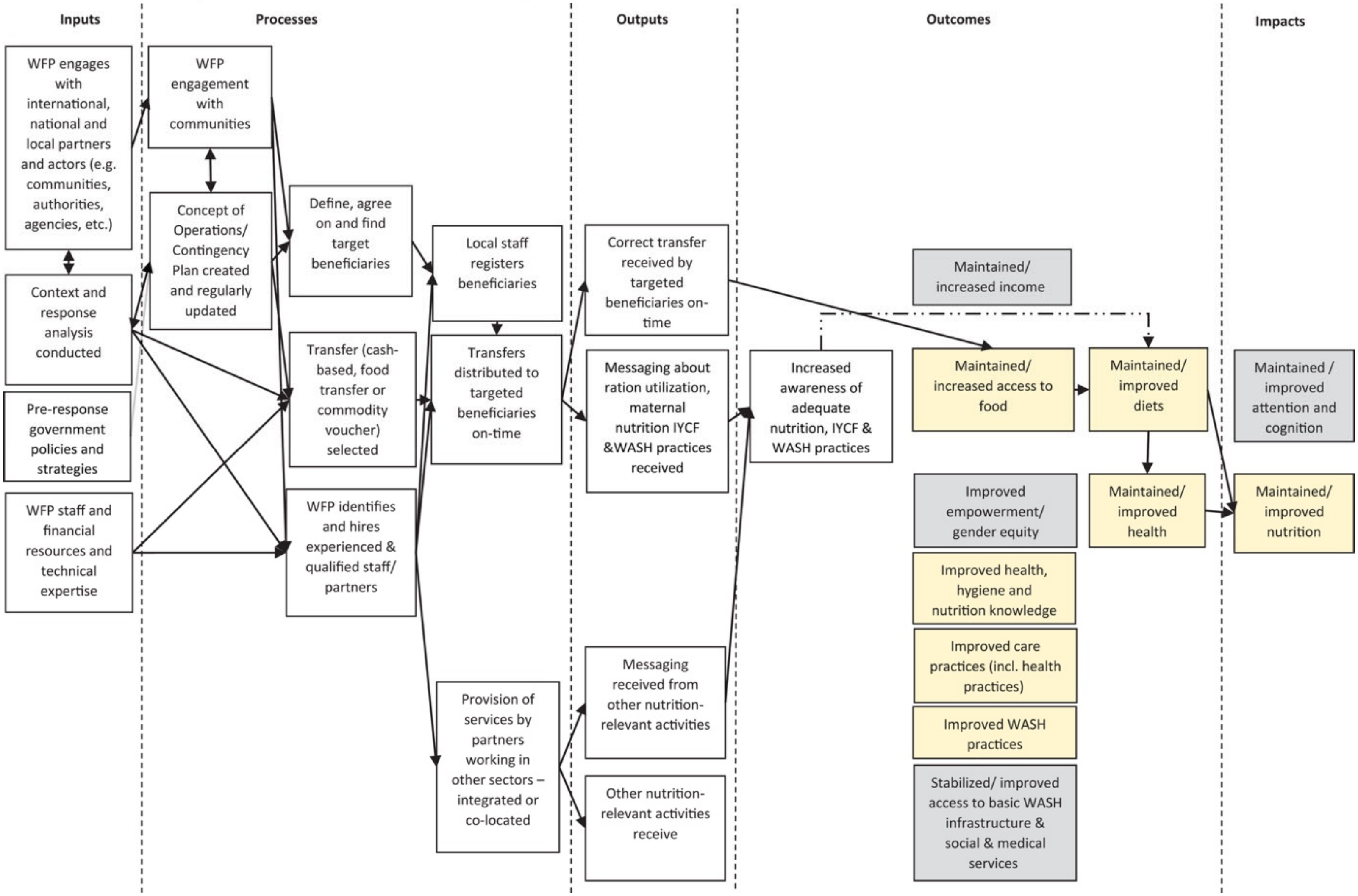

Figure 2. General food assistance (Emergency Context) program: Nutrition-sensitive theory of change. Gray boxes identify underlying determinants of nutrition that this type of program is not currently working toward achieving. These underlying determinants may be addressed through other types of World Food Program (WFP) programs. Yellow boxes identify outcomes or impacts related to reducing households' and people's vulnerability to future shocks and strengthening their resilience. Solid arrows indicate links that currently exist within the program and/or for which there is some evidence that the link works as indicated. Dashed arrows indicate links that are dependent on the specific activities chosen or for which there is currently limited evidence
